# A computational evaluation of over-representation of regulatory motifs in the promoter regions of differentially expressed genes

**DOI:** 10.1186/1471-2105-11-267

**Published:** 2010-05-20

**Authors:** Guofeng Meng, Axel Mosig, Martin Vingron

**Affiliations:** 1 CAS-MPG Partner Institute and Key Laboratory for Computational Biology, Shanghai Institutes for Biological Sciences, 320 Yue Yang Road, 200031, Shanghai, China; 2 Max Planck Institute for Mathematics in the Sciences, Inselstrasse 22, 04103 Leipzig, Germany; 3 Max Planck Institute for Molecular Genetics, 14195 Berlin, Germany

## Abstract

**Background:**

Observed co-expression of a group of genes is frequently attributed to co-regulation by shared transcription factors. This assumption has led to the hypothesis that promoters of co-expressed genes should share common regulatory motifs, which forms the basis for numerous computational tools that search for these motifs. While frequently explored for yeast, the validity of the underlying hypothesis has not been assessed systematically in mammals. This demonstrates the need for a systematic and quantitative evaluation to what degree co-expressed genes share over-represented motifs for mammals.

**Results:**

We identified 33 experiments for human and mouse in the ArrayExpress Database where transcription factors were manipulated and which exhibited a significant number of differentially expressed genes. We checked for over-representation of transcription factor binding sites in up- or down-regulated genes using the over-representation analysis tool oPOSSUM. In 25 out of 33 experiments, this procedure identified the binding matrices of the affected transcription factors. We also carried out *de novo *prediction of regulatory motifs shared by differentially expressed genes. Again, the detected motifs shared significant similarity with the matrices of the affected transcription factors.

**Conclusions:**

Our results support the claim that functional regulatory motifs are over-represented in sets of differentially expressed genes and that they can be detected with computational methods.

## Background

Patterns of differential gene expression in organisms are known to result from a complex and dynamic gene regulatory network, where the interactions between transcription factors (TFs) and their target genes take center stage. Therefore, the activation or deactivation of TFs in specific signaling pathways triggers up- or down-regulation of their direct targets. Those effects have been subject of numerous studies dealing with different signaling pathways such as development and hormone signaling [1-4]. For some of these processes, it is well understood how TFs directly transform regulatory signals into gene expression levels by binding to proximal or distal promoters of genes.

The roles of TFs in regulating gene expression have been widely observed in microarray experiments, in which TF genes were knocked out, over-expressed, or stimulated with ligands [5-21]. These studies generally investigated the change of gene expression induced by altering the activity of certain TFs and approved the roles of TFs in gene expression. Furthermore, computational studies have also demonstrated that genes with common regulatory binding sites are more likely to have similar expression profiles [22,23]. The importance of TFs in gene expression regulation naturally raises the question to what degree differential expression of genes under different conditions indicates the presence of shared regulatory motifs. If so, this provides a useful theoretical foundation for novel motif prediction and functional studies. Indeed, it has been a widely used and accepted hypothesis that co-expressed genes share common regulatory motifs. It serves as a useful working hypothesis in many scenarios, and numerous computational tools for regulatory motif discovery built with considerable success on this hypothesis [24-36]. While it has been fully explored and approved in yeast [37-39], little is known about the applicability of this working hypothesis for mammals.

Considering the rather anecdotal basis for its acceptance, the hypothesis of co-expressed genes sharing common regulatory motifs calls for a systematic evaluation. In fact, microarray experiments in public databases are now widely available, providing expression profiles of thousands of genes under numerous different conditions on a genome-wide scale. As these data are a popular basis for regulatory motif discovery, there is a big demand for a systematic evaluation of the underlying hypothesis.

In this work, we analyzed differentially expressed genes in microarray experiments from the ArrayExpress database [40] that were related to transcription factor activity modifications. We particularly analyzed such experiments, where the perturbation was aimed at a transcription factor. This setting allows us to test whether we are able to recover binding sites of the altered transcription factor from the differential genes alone. This is clearly not trivial because the set of differential genes will encompass a whole cascade of up- or down-regulated genes due to the initial perturbation. Although the microarray database contains many more experiments from which co-expressed genes could be derived, we focus on the ones where we know the identity of the causal transcription factor, such that we can evaluate the success rate of our recovery method.

**Figure 1:**
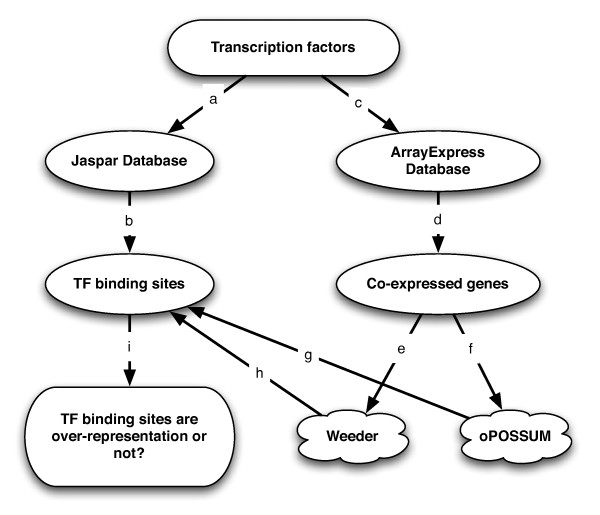
**The pipeline for over-representation evaluation of TF binding sites**. (a) and (b) TF binding sites were described with the matrices in Jaspar Database; (c) experiments with modified TF activity were selected from the ArrayExpress Database and (d) analyzed with computational methods for differentially expressed genes; (e) and (f) over-represented motifs or matrices were predicted using Weeder and oPOSSUM and those motifs (h) or matrices (g) were compared with the target matrices to check whether the binding sites of affected TFs were recovered.

We study two approaches toward checking whether the binding sites of the affected TFs are over-represented in the differentially expressed genes. In the first approach, we use oPOSSUM[25] to analyze the over-representation of Jaspar matrices [41,42], which represent profiles of binding sites derived from known TF binding sites. Among these matrices, we focus our attention on the matrices corresponding to the affected TFs, which we will hence refer to as *target matrices *throughout the rest of this paper. We apply oPOSSUM to evaluate the over-representation of target matrices in the promoter regions of differentially expressed genes according to a probabilistic scoring scheme. The second approach we investigate is based on *de novo *predictions using Weeder[43]. This motif finding tool computes *de novo *motif predictions, which allow us to compare the similarity between those predictions and matrices in the Jaspar database. High similarity suggests that affected TF binding sites were recovered in *de novo *prediction. Figure 1 shows the basic workflow of these two approaches.

## Methods

### Description of TF binding sites

Recognition of TF binding sites in promoter regions of differentially expressed genes was performed by detecting over-represented position frequency matrices (PFMs), which were taken from the publicly available Jaspar database [41,42]. This database contains a set of 138 matrices representing experiment-determined binding profiles, including 101 matrices for vertebrate TFs. We used percent similarity scores, predicted by Jaspar web-interfaced tool for similarity comparison of different Jaspar matrices [44]. Percent similarity has a maximal score of 100%, which indicates the highest similarity.

### Microarray experiment selection and analysis

To obtain a set of suitable microarray experiments, we searched the ArrayExpress database for experiments with modified TF activity. We searched the TFs against the ArrayExpress database [40]. We verified the relationship of the TFs with the associated experiments by inspecting the literature references or experiment descriptions, and selected those experiments where TFs or their genes were modified by the experimental methods. The TF activity modifications we encountered included gene knockout, transgenic over-expression, ligand stimulation or stimulation by mimicking the action of transcription factor, among others.

Most of the microarray experiments in the ArrayExpress database provide both raw and processed (or normalized) data. In this work, we preferably chose the former. Raw data were normalized by RMA [45], a popular normalization method for Affymetrix data, with default parameter setting, as implemented in the R affy package. Then, the SAM [46] method was used for differential expression analysis and p-value was assigned to each gene for its significance of differentially expression. We sorted genes with ascending p-value as a gene list. In next step, we would choose the top *n *genes for over-representation and *de novo *prediction analysis, where *n *was an parameter for input gene number, e.g. set to *n *= 100, *n *= 200 or *n *= 400. For many of the experiments SAM did not return any differentially expressed genes with certain arbitrary cutoff. In search of the reason for this we studied the quality of the experiments from the database. In principle, microarray experiments involve a number of steps that are prone to errors, which may significantly distort the outcome of subsequent analysis. We studied primarily two criteria for the quality of an experiment. The first one was based on scatter plots, in which the averaged normalized expression level of one condition was plotted against that of another condition. For a meaningful microarray experiments, most of genes lies around diagonal line while differentially expressed genes are recognized by their distance to the main diagonal [47]. Another criterion for the quality of an experiment is the distribution of the p-value computed by SAM. Informative experiments should show a distribution of p-values which is roughly uniform in general with an increase or a peak for small p-values [48]. For all experiments, we inspected both scatter plot and p-value histogram and excluded experiments that did not obey the above criteria. All these plots are available in Additional file 1.

### Over-representation of Jaspar matrices

Numerous tools for finding over-represented regulatory motifs in differentially expressed genes are available [49]. Among them, we employed oPOSSUM[24,25] for over-representation analysis. oPOSSUM is a tool that combines the phylogenetic footprinting method with statistical approaches for identifying over-represented Jaspar matrices in a set of co-expressed genes; it takes gene IDs as input and ranks matrices by two scores to describe their over-representation significance, namely the z-score and the Fisher-score.

While there is no systematic comparison between the performance of different over-representation analysis tools, we relied on the oPOSSUM tool for several reasons. First of all, oPOSSUM is relatively fast if the number and lengths of promoters are within reasonable bounds. Furthermore, oPOSSUM can handle long promoter sequences ranging from -20, 000 bp to +20, 000 bp around the transcription start site (TSS) and takes into account TF binding sites throughout this full range. As another advantage over other over-representation analysis tools, oPOSSUM uses phylogenetic footprinting to improve performance. Finally, the authors of oPOSSUM validated its performance with NF-κB microarray experiments and random sampling data in a setting that is similar to ours [24].

The oPOSSUM tool allows the user to specify a number of parameters, including species, Jaspar matrices, level of conservation (background conservation), matrix match threshold, promoter length, and display option. For most of the tested cases, top 30% conservation, 85% matrix match threshold and 200 input sequences with -2000 to +2000 bp around the TSS (+1 bp) were good choices (see Additional file 2). We set those parameters for all the experiments as default parameter setting. Whenever we did not find the target matrices to be over-represented under those settings, we manually tried different setting of promoter number and length to check whether target matrices would rank among the over-represented matrices. We followed the suggestion by the authors of oPOSSUM that motifs with a z-score exceeding 10 and a Fisher-score below 0.01 could be considered *significantly over-represented *[25]. However, when the target matrices satisfied only one of the above cutoffs, we would treat it as *weakly over-represented*. Hence, for each experiment, according to the z-scores and Fisher-scores, target matrices would be categorized as either significantly over-represented (S), weakly over-represented (W), or not over-represented (N).

### De novo prediction of motifs

To further study over-representation of target matrices in promoter regions of co-expressed genes, we predicted over-represented motifs using *de novo *motif finding methods. In choosing an appropriate *de novo *motif finding tool among the numerous available approaches, we followed the systematic evaluation by Tompa et al. [50], which found the Weeder tool [43] particularly successful in the context of binding site discovery. Using the same settings as with oPOSSUM (promoters of 200 top ranking differential genes, -2000 to +2000 bp around the TSS), we further analyzed all experiments using Weeder. Each run of Weeder predicted 10 motif profiles by default. We then compared the similarity between those motifs and the Jaspar target matrices using the Jaspar web-interface tool [44] and recorded the percent similarity score for the most similar pairs.

## Results and Discussion

### Microarray analysis

We searched the ArrayExpress database for experiments involving hybridizations that differed in loss or gain of the function of a specific TF. We retrieved 88 microarray experiments for human and mouse. Those experiments cover a whole bandwidth of methods to modify the activity of TFs; at least 59 experiments involve methods that decreased the activity of TFs such as gene knockout or RNAi. In more than 34 experiments, TF activity was increased by techniques such as ligand stimulation, or transgenic over-expression. A summary of TF activity modifications used is given in Additional file 3.

In the process of eliminating low-quality experiments, we excluded 11 experiments that either had only one replication, or where our standard analysis procedure reported errors without clear reason. For the remaining experiments, we manually assessed the microarray quality based on scatter plots and p-value frequency distribution (see Additional file 1). Whenever the scatter plot or p-value distribution was obviously unreasonable, which indicates some problems of the underlying experiment, we excluded them from further step. As a result, the differentially expressed genes in 33 out of the 77 experiments were used for over-representation and *de novo *analysis. The following TFs were perturbed in those 33 experiments: *cMyc, ESRalpha, irf1, HNF4a, nmyc, Myf, FoxQ, Myb NFkappaB2, AIbZIP, HiF1, Cepba, Evi1, Foxa2, CREB, PPARg2, p53, PPARalpha, PPARI, USF1, IRF6, HMGA2, STAT2, e2f2, HNF1a, Mef2c, Gata-1*, *KLF15, Nkx2.5 and Gata-3*. They are associated with 30 target matrices in the Jaspar database. We summarize those TFs and their target matrices in Table 1. In the next step of our work, we would evaluate the over-representation of those target matrices in promoter regions of differentially expressed genes. According to the classification of Jaspar matrices by Sandelin and Wassserman, these TFs cover nine out of the 11 TF classes identified in [41] (see Additional file 4). Besides of the matrices falling into these nine familial profiles, another eight out of 30 target matrices remain unclassified in the scheme by Sandelin and Wasserman.

**Table 1:** Transcription factors and their Jaspar target matrices.

Experiment Name	TF Name	**Jaspar****Class**	**Jaspar****target matrices**
E-GEOD-10954	cMyc	bHLH-ZIP	MYC-MAX, MAX, Mycn

E-GEOD-11039	e2f2	E2F_TDP	E2F1

E-GEOD-11352	ESRalpha	Nuclear Receptor	ESR1

E-GEOD-11809	irf1	TRP-CLUSTER	IRF1 IRF2


E-GEOD-2060	CREB	bZIP	CREB1, bZIP910, bZIP911

E-GEOD-2192	PPARg2	Nuclear Receptor	PPARG-RXRA, PPARG

E-GEOD-2527	Gata-1	ZN-FINGER, GATA	Gata1

E-GEOD-3126	HNF4a	NUCLEAR RECEPTOR	HNF4A

E-GEOD-3244	p53	TP53	P53

E-GEOD-6077	nmyc	bHLH-ZIP	Mycn, MYC-MAX, MAX

E-GEOD-6487	Myf	bHLH	Myf

E-GEOD-7137	KLF15	ZN-FINGER, C2H2	Klf4

E-GEOD-7219	NFkappaB2	REL	NF-kappaB, NFKB1

E-GEOD-7223	AIbZIP	bZIP	CREB1, bZIP910, bZIP911

E-GEOD-7835	HiF1	bHLH	Arnt, Arnt-Ahr

E-GEOD-9786	PPARalpha	Nuclear Receptor	PPARG, PPARG-RXRA

E-MEXP-1444	Cepba	bZIP	Cebpa, Ddit3-Cebpa

E-MEXP-634	Gata-3	ZN-FINGER, GATA	GATA3

E-GEOD-590	USF1	Zipper	USF1

E-GEOD-5800	Irf6	TRP-CLUSTER	IRF1 IRF2

E-GEOD-5823	c-MYC	bHLH-ZIP	Mycn, MYC-MAX, MAX

E-GEOD-2624	NF-kB	REL	NF-kappaB, NFKB1

E-GEOD-3116	HNF4	NUCLEAR RECEPTOR	HNF4A

E-GEOD-5424	Foxa2	Forkhead	FOXF2, FOXD1, FOXC1, FOXL1, Foxq1, Foxd3, Foxa2, FOXI1

E-GEOD-8943	FOXQ1	Forkhead	FOXF2, FOXD1, FOXC1, FOXL1, Foxq1, Foxd3, Foxa2, FOXI1

E-GEOD-11557	Evi-1	zinc finger	Evi1

E-TABM-43	TP53	TP53	P53

E-GEOD-2815	Myb	Helix-Turn-Helix	Myb

E-GEOD-5475	PPARI	Nuclear Receptor	PPARG, PPARG-RXRA

E-GEOD-6846	STAT2	stat	STAT1

E-GEOD-11836	Nkx3.1	HOMEO	Nkx2-5

E-MEXP-871	HMGA2	-	HMG-1, HMG-IY

E-MEXP-1413	E2F2	E2F TDP	E2F1

### A case-study for over-representation

In order to illustrate our procedure, we take an exemplary in-depth look at the *estrogen receptor α *(ER*α*). The estrogen receptor (ER) is a ligand-dependent TF that can be activated by estrogen; ER can recognize short DNA sequences, the so-called estrogen response elements (EREs) (5'-GGTCAnnnTGACC-3') in the proximal and distal promoters of genes and regulates gene expression [51]. Here we used the microarray experiment supplied by Lin et al. in the ArrayExpress database (ArrayExpress ID *E-GEOD-11352*) [8]. In their work, cells in a estrogen-receptor positive breast cancer cell line (MCF7) were either exposed to 10 nM estradiol (a sex hormone, the major estrogen of human) or control only. Then sampled cells were prepared for microarray analysis at the time-points of 12, 24 and 48 hours; each sample hybridization was repeated three times. In this way, the authors obtained 18 hybridizations. We used SAM for differential expression and all the genes were assigned with *p*-values, which indicated the significance of differentially expression. We then sorted those genes according to their p-values and formed a gene list. The top n up-or down-regulated genes were selected as input of oPOSSUM analysis.

**Figure 2:**
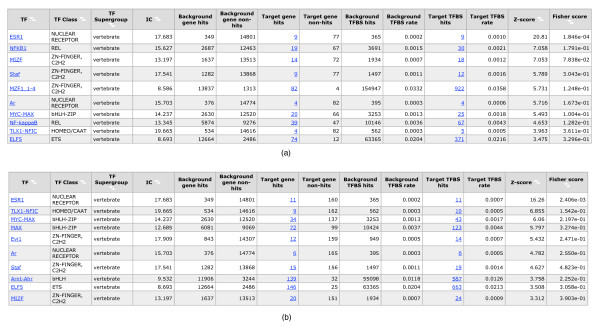
**Over-representation of ESR1 in up-regulated genes**.  (a) 100 up-regulated genes and (b) 200 up-regulated genes were input into oPOSSUM.

In the Jaspar database, we identified matrix *ESR1 *as a profile for ER*α *binding sites. Figure 2 shows the output of oPOSSUM with different gene numbers. In this example, we used the top 100 and top 200 up-regulated genes, respectively, of gene list as input to oPOSSUM, with background conservation of 30% and sequences from -2, 000 to +2, 000 bp around the TSS. Under both conditions, oPOSSUM found *ESR1 *as a top ranked matrix under both the Fisher-score and the z-score, which satisfied the thresholds for significant over-representation. This demonstrates that ER binding sites are indeed over-represented in differentially expressed gene promoters, and that this over-representation can be recovered computationally.

Beside *ESR1*, we also found other matrices, such as *Ar*, *TLX1-NFIC *and *NFKB1*. However, those matrices were not as significantly over-represented as *ESR1*. Since frequently several transcription factors are involved in regulating gene expression [52-56], it is conceivable that the additional matrices are reflections of interacting TFs rather than false positive discoveries. Without further experimental evidence, however, it is hard to tell in general, even if they are only weakly over-represented.

As it turned out, input promoter number and promoter length had a great influence on the sensitivity of oPOSSUM. Hence, we used the ESR1 matrix as a showcase to evaluate different parameter settings for oPOSSUM. The following points summarized our findings:

1. *ESR1 *can be detected as over-represented in a wide range of promoter lengths from 4000 bp to 7000 bp. One possible reason for this is that ER can bind to proximal promoters as well as distal ones [8]. However, the region stretching from -2000 bp to +2000 bp around the TSS is the preferred region.

2. *ESR1 *can be found significantly over-represented in up-regulated genes under different numbers of up-regulated genes, ranging between 40 and 800 genes. For the down-regulated genes, *ESR1 *was found to be over-represented when the gene number was greater than 400, however, at a very low level of significance.

The importance of parameter settings for the performance of oPOSSUM might indeed reflect properties how a specific TF regulates its targets. For example, the *ESR1 *matrix was recognized as significantly over-represented by oPOSSUM in promoters ranging from -2000 bp to +2000 bp around the TSS, but not in the range of -2000 to 0 bp around the TSS, which might indicate the distribution of TF binding sites in promoter regions. Indeed, this had already been addressed specifically for the *ER *transcription factor by Lin et. al. Their Chip-PET experiment showed that the largest fraction (38%) of binding regions mapped to intragenic regions of transcripts and were localized within introns, whereas 23% were within 100 kb from the 5' start sites, and 19% were within 100 kb of 3' polyadenylation sites [8]. This clearly indicated significant enrichment of ER binding sites in downstream regions of promoters. This is in line with our observation that ignoring the promoter ranging from 0 to +2000 bp makes *ESR1 *not discoverable by over-representation analysis. This allows the conclusion that over-representation conditions reflect the distribution of TF binding sites, which is an important aspect in choosing proper promoter regions in our motif finding and analysis.

### Systematic analysis of performance of over-representation analysis

As the above example demonstrates, the *ESR1 *binding site can be recovered through over-representation analysis in up-regulated genes. To see whether this carries over to other TFs, we proceeded to analyze the remaining experiments for which we had identified differential genes in the microarray experiments (see Methods). Under the default parameter settings, we repeated the above process for over-representation analysis with oPOSSUM. We summarized the result of oPOSSUM analysis in Table 2 (for detailed result, see Additional file 5). Under default parameter settings, up to 12 target matrices were found to be significantly over-represented in either of up- or down-regulated genes. In seven experiments, target matrices were over-represented at a low level of significance. Due to the great influence of parameters, for those 21 experiments whose target matrices were not significantly over-represented under default parameter settings, we subsequently altered input promoter number and length, leading to the identification of significantly over-represented target matrices in seven experiments and two new weakly over-represented experiments. The remaining eight experiments did not yield any of the target matrices to satisfy z-score above 10 or Fisher-score below 0.01. For all the experiments, we also recorded conditions which recovered the target matrices as over-represented at highest possible level of significance (see Table 2).

**Table 2:** Results for over-representation analysis in 33 experiments

Experiment	Name. TF	default parameter setting		Con. Most significantly over-representation	
		
		Up-regulated genes	Down-regulated genes	Parameter setting	status
E-GEOD-10954	cMyc	N	S	400, down-regulated, 10000 bp	S

E-GEOD-11352	ESRalpha	S	N	100, up-regulated, 4000 bp	S

E-GEOD-11809	irf1	S	W	100, up-regulated, 4000 bp	S

E-GEOD-3126	HNF4a	S	N	400, up-regulated, 4000 bp	S

E-GEOD-6077	nmyc	S	N	100, up-regulated, 7000 bp	S

E-GEOD-6487	Myf	S	N	400, up-regulated, 4000 bp	S

E-GEOD-7219	NFkappaB2	S	N	200, up-regulated, 7000 bp	S

E-GEOD-7223	AIbZIP	S	N	200, up-regulated, 4000 bp	S

E-GEOD-7835	HiF1	N	S	400, up-regulated, 7000 bp	S

E-MEXP-1444	Cepba	S	W	100, up-regulated, 7000 bp	S

E-GEOD-2624	NF-kB	N	S	200 down-regulated, 2000 bp	S

E-GEOD-11557	Evi-1	N	S	200 down-regulated, 2000 bp	S

E-GEOD-5424	Foxa2	W	N	300 up-regulated, 7000 bp	S

E-TABM-43	TP53	W	N	200 up-regulated, 2000 bp	S

E-GEOD-3116	HNF4	W	N	100 up-regulated, 2000 bp	S

E-GEOD-2060	CREB	N	N	400, up-regulated, 7000 bp	S

E-GEOD-3244	p53	N	N	100, up-regulated, 7000 bp	S

E-GEOD-9786	PPARalpha	N	N	100, down-regulated, 2000 bp	S

E-GEOD-5475	PPARI	N	N	100 down-regulated, 7000 bp	S

E-GEOD-2192	PPARg2	W	N	200, up-regulated, 4000 bp	W

E-GEOD-11039	e2f2	W	N	100, up-regulated, 4000 bp	W

E-GEOD-590	USF1	N	N	300 up-regulated, 7000 bp	W

E-GEOD-5800	Irf6	N	N	100 up-regulated, 4000 bp	W

E-GEOD-8943	FOXQ1	W	N	200 up-regulated, 4000 bp	W

E-GEOD-5823	c-MYC	W	W	300 up-regulated 4000 bp	W

E-GEOD-2527	Gata-1	N	N	-	-

E-GEOD-7137	KLF15	N	N	-	-

E-MEXP-634	Gata-3	N	N	-	-

E-GEOD-2815	Myb	N	N	-	-

E-GEOD-6846	STAT2	N	N	-	-

E-GEOD-11836	Nkx3.1	N	N	-	-

E-MEXP-871	HMGA2	N	N	-	-

E-MEXP-1413	E2F2	N	N	-	-

S: significantly over-represented; W: weakly over-represented; N: not over-represented

We proceeded to determine whether this success rate could actually be due to chance. For all the tested experiments, oPOSSUM found on average 3.5 matrices to be significantly over-represented per analysis, out of which one happened to be the target matrix. We determined the probability of this event by comparing to the overall number of candidate matrices in Jaspar. Then, the event of finding the target matrix in a certain number of cases is binomially distributed. We found target matrices to be over-represented in 25 out of 33 experiments, including significantly over-representation in 19 experiments. In fact, the significance of finding the target matrix to be significantly over-represented out of 33 cases has a binomial tail probability below 2.2*e *- 16, which makes it appear highly unlikely that this performance would be due to chance rather than the ability of the computational pipeline to pick up the right matrix. We recovered target matrices correspond to 9 out of the 11 TF classes identified in [41], with the exceptions coming from the *HMG *and *Homeobox *groups of transcription factors. The number of experiments we had available for study seems to be insufficient, however, to fully decide whether this represents a bias in over-representation analysis with respect to certain TF classes.

For all those 19 experiments where target motifs appeared significantly over-represented under default parameter setting, target matrices were found significantly over-represented in either up-regulated genes or down-regulated genes, but never in both. Although in some experiments, target matrices were also weakly over-represented, we can still conclude that TFs generally play unequal roles in activating and repressing gene expression.

In this work, eight experiments did not allow us to identify the target matrices to be over-represented. There might be a plethora of reasons for this. First of all, we evaluated the hypothesis that the information content of PFMs had great influences on the performance of oPOSSUM. PFMs with low information content would be likely to lead to more false positive binding site predictions, which results in low performance of oPOSSUM. Therefore, we carried out a Student's t-test for information content of over-represented and not over-represented matrices. The result showed a great difference in information content (one-tailed p < 0.029). Although this was in line with our hypothesis, we still could not ascribe all failures to recover matrices to low information content. Another hypothesis we investigated was that the real distribution of TF binding sites was out of the ability of oPOSSUM. As two experiments related to *Gata *factors did not yield over-represented target matrices, we investigated their properties in more detail. Although ChIP-chip experiments were available that indicated the binding sites of *Gata *factors in proximal promoters [57], many experiments suggested that *Gata *factors took important roles by binding to regions out of -2000 bp and +2000 bp of the TSS [58,59]. Together with seven other TFs, no whole-genome binding site investigation was available in public databases, making it hard to draw conclusions without further experimental data. A final reason why over-representation might fail in some cases lies in the networked nature of regulation by transcription factors. TFs do not act purely by themselves, but interact with other TFs through a cascade of signals. In microarray experiments, genes with differential expression may not be the direct target of TFs. For example, *c-myc *can be regulated by other TFs, and *c-myc *may also regulate about 15% of all other genes, including numerous other TF genes [60,61]; under such conditions, it is hard to distinguish signals directly induced by a TF from such cascaded "second-round" signals.

### De novo prediction

**Figure 3:**
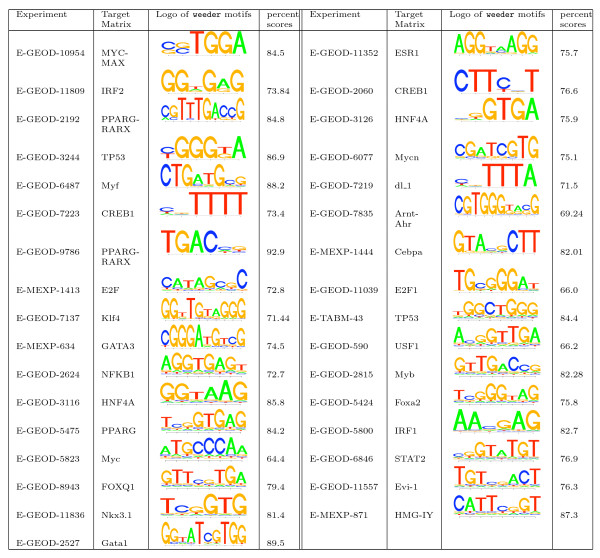
**Regulatory motifs predicted by Weeder and their similarity with target matrices**.

**Figure 4:**
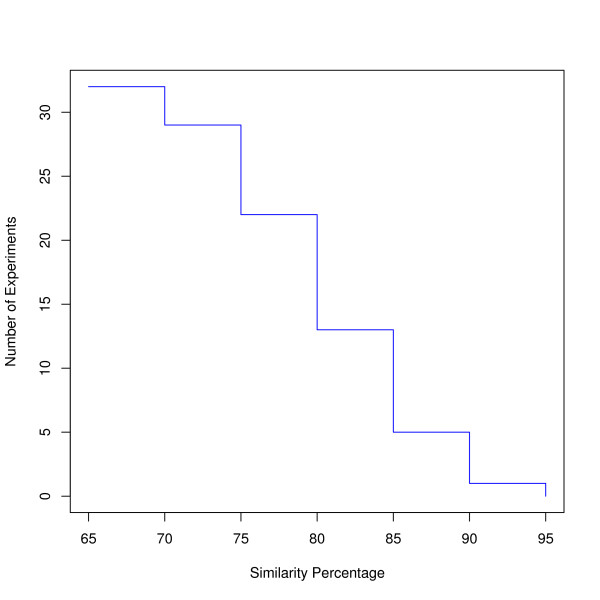
**Number of recovered experiments under different similarity percentage**.

In the previous step, oPOSSUM was applied to determine over-represented TF binding sites related Jaspar matrices in differentially expressed genes. A natural next step was to determine whether those regulatory motifs could also be recovered by *de novo *predictions. We performed *de novo *prediction in promoter regions of differentially expressed genes using the Weeder tool [43]. Figure 3 shows the logos for predicted motifs and their similarity with target matrices. In order to evaluate how well target matrices could be recovered by Weeder, we summarized the number of experiments with recovered target matrices at different similarity percentage cutoffs, as shown in Figure 4. For all the experiments, Weeder predicted at least one motif sharing ≥ 60% similarity and the number of recovered experiments decreased with stricter similarity percentage cutoff. Considering the nature of TF binding sites and the mechanism of *de novo *prediction methods [62], we could not expect the predicted motifs to share a high degree of similarity with the target matrices. If we set the similarity cutoff 75% for recovering target matrices, our predictions could recover the TF binding sites in about 73% of the 33 experiments. In general, we may conclude that the affected TF binding sites can indeed be recovered in many cases using *de novo *prediction methods.

## Conclusions

In this work, we report a computational evaluation on recovering TF binding sites from differentially expressed genes using two different methods. Our over-representation analysis with oPOSSUM proves successful in 25 out of 33 experiments exhibiting differential expression patterns as a consequence of activating or deactivating TFs, indicating that TF binding site recovery is generally possible with computational methods when dealing with one single manipulated transcription factor. Our evaluation of *de novo *prediction for all experiments succeeds in recovering motifs similar to the binding site of the affected TFs in about 73% of all cases with a cutoff of similarity percentage of 75%. This allows the conclusion that TF binding site recovery may even be achieved using a *de novo *approach, though less reliable than oPOSSUM over-representation analysis.

In general, our findings support the hypothesis that the over-representation of TF binding sites in the promoter regions of differentially expressed genes can be detected with computational tools and it confirms that TF binding sites can be predicted by utilizing information of differential expression. With the increasing availability of microarray data in public databases, it will be a useful theoretical foundation for novel TF binding site prediction and functional studies.

In this work, we could also specify the influence of input gene numbers and promoter length and their importance for the sensitivity of computational methods, which indicates the different properties of TF regulating gene expression. More specifically, we could observe very particular regulatory effects such as the critical effect of downstream *ESR1 *binding sites on gene expression.

## Authors' contributions

GM collected data, performed data analysis and drafted the manuscript; AM participated in its design, coordination and drafted the manuscript; MV conceived the study, and participated in its design; All authors approved the final manuscript.

## Supplementary Material

Additional file 1 **Assessment of microarray quality with scatter plots and p-value frequency histograms**. To eliminate those microarrays with low quality, we used two methods for quality evaluation. The first one was based on scatter plots, in which the averaged normalized expression value of manipulated hybrids and control hybrids were plotted. Another methods was histograms of q-value frequency distributions, predicted by SAM. We manually checked those distribution and selected reasonable experiments for differential expression analysis. Click here for file

Additional file 2 **The parameter preference of **oPOSSUM. In this figure, we described number of experiments with significantly over-represented TF binding sites under different promoter number and length. Click here for file

Additional file 3 **Information for 88 microarray experiments in this work**. By searching the TFs in ArrayExpress Database, we got 88 TF-related experiments. In this file, we listed the detailed information of those experiments, including organism, experimental methods, tissues, TF names, references, experiment titles in ArrayExpress Database. Those experiments were used for differential expression analysis. Click here for file

Additional file 4 **Target matrices mapped to familial binding profiles of **Jaspar**matrices**. Sandelin and Wasserman had classified the Jaspar matrices into the 11 *familial binding profiles*, which was based on TF structural information as well as binding matrix similarity of Jaspar matrices [41]. We highlighted the 30 target matrices associated with our work to the each of these 11 familial classes. Click here for file

Additional file 5 **The output of **oPOSSUM**for 33 experiments**. The differentially expressed genes from microarray analysis were input into oPOSSUM for over-representation analysis. In this file, we gave outputs of oPOSSUM for each experiment and supplied 33 tables for over-representation status under different promoter number and length. Click here for file

## References

[B1] McCourt P GENETIC ANALYSIS OF HORMONE SIGNALING Annu Rev Plant Physiol Plant Mol Biol 1999 50 219 243 10.1146/annurev.arplant.50.1.219 15012209

[B2] Stathopoulos A Levine M Genomic regulatory networks and animal development Dev Cell 2005 9 4 449 462 10.1016/j.devcel.2005.09.005 16198288

[B3] Freeman M Gurdon JB Regulatory principles of developmental signaling Annu Rev Cell Dev Biol 2002 18 515 539 10.1146/annurev.cellbio.18.012502.083458 12142269

[B4] Sancho E Batlle E Clevers H Signaling pathways in intestinal development and cancer Annu Rev Cell Dev Biol 2004 20 695 723 10.1146/annurev.cellbio.20.010403.092805 15473857

[B5] Akpinar P Kuwajima S Krutzfeldt J Stoffel M Tmem27: a cleaved and shed plasma membrane protein that stimulates pancreatic beta cell proliferation Cell Metab 2005 2 6 385 397 10.1016/j.cmet.2005.11.001 16330324

[B6] Reymann S Borlak J Transcription profiling of lung adenocarcinomas of c-myc-transgenic mice: identification of the c-myc regulatory gene network BMC Syst Biol 2008 2 46 10.1186/1752-0509-2-46 18498649 PMC2430022

[B7] Andrechek ER Mori S Rempel RE Chang JT Nevins JR Patterns of cell signaling pathway activation that characterize mammary development Development 2008 135 14 2403 2413 10.1242/dev.019018 18550711 PMC3615553

[B8] Lin CY Vega VB Thomsen JS Zhang T Kong SL Xie M Chiu KP Lipovich L Barnett DH Stossi F Yeo A George J Kuznetsov VA Lee YK Charn TH Palanisamy N Miller LD Cheung E Katzenellenbogen BS Ruan Y Bourque G Wei CL Liu ET Whole-genome cartography of estrogen receptor alpha binding sites PLoS Genet 2007 3 6 e87 10.1371/journal.pgen.0030087 17542648 PMC1885282

[B9] Aly S Mages J Reiling N Kalinke U Decker T Lang R Ehlers S Mycobacteria-induced granuloma necrosis depends on IRF-1 J Cell Mol Med 2008 18705699 10.1111/j.1582-4934.2008.00470.x PMC6512360

[B10] Zhang X Odom DT Koo SH Conkright MD Canettieri G Best J Chen H Jenner R Herbolsheimer E Jacobsen E Kadam S Ecker JR Emerson B Hogenesch JB Unterman T Young RA Montminy M Genome-wide analysis of cAMP-response element binding protein occupancy, phosphorylation, and target gene activation in human tissues Proc Natl Acad Sci USA 2005 102 12 4459 4464 10.1073/pnas.0501076102 15753290 PMC555478

[B11] Akerblad P Mansson R Lagergren A Westerlund S Basta B Lind U Thelin A Gisler R Liberg D Nelander S Bamberg K Sigvardsson M Gene expression analysis suggests that EBF-1 and PPARgamma2 induce adipogenesis of NIH-3T3 cells with similar efficiency and kinetics Physiol Genomics 2005 23 2 206 216 10.1152/physiolgenomics.00015.2005 16106032

[B12] Muntean AG Crispino JD Differential requirements for the activation domain and FOG-interaction surface of GATA-1 in megakaryocyte gene expression and development Blood 2005 106 4 1223 1231 10.1182/blood-2005-02-0551 15860665 PMC1895209

[B13] Battle MA Konopka G Parviz F Gaggl AL Yang C Sladek FM Duncan SA Hepatocyte nuclear factor 4alpha orchestrates expression of cell adhesion proteins during the epithelial transformation of the developing liver Proc Natl Acad Sci USA 2006 103 22 8419 8424 10.1073/pnas.0600246103 16714383 PMC1482507

[B14] Ishibashi J Perry RL Asakura A Rudnicki MA MyoD induces myogenic differentiation through cooperation of its NH2- and COOH-terminal regions J Cell Biol 2005 171 3 471 482 10.1083/jcb.200502101 16275751 PMC2171269

[B15] Cox B Kislinger T Wigle DA Kannan A Brown K Okubo T Hogan B Jurisica I Frey B Rossant J Emili A Integrated proteomic and transcriptomic profiling of mouse lung development and Nmyc target genes Mol Syst Biol 2007 171 3 109 10.1038/msb4100151 PMC2673710 17486137

[B16] Gray S Wang B Orihuela Y Hong EG Fisch S Haldar S Cline GW Kim JK Peroni OD Kahn BB Jain MK Regulation of gluconeogenesis by Kruppel-like factor 15 Cell Metab 2007 5 4 305 312 10.1016/j.cmet.2007.03.002 17403374 PMC1892530

[B17] Lind EF Ahonen CL Wasiuk A Kosaka Y Becher B Bennett KA Noelle RJ Dendritic cells require the NF-kappaB2 pathway for cross-presentation of soluble antigens J Immunol 2008 181 354 363 18566401 10.4049/jimmunol.181.1.354

[B18] Ben Aicha S Lessard J Pelletier M Fournier A Calvo E Labrie C Transcriptional profiling of genes that are regulated by the endoplasmic reticulum-bound transcription factor AIbZIP/CREB3L4 in prostate cells Physiol Genomics 2007 31 2 295 305 10.1152/physiolgenomics.00097.2007 17712038

[B19] Rosen MB Lee JS Ren H Vallanat B Liu J Waalkes MP Abbott BD Lau C Corton JC Toxicogenomic dissection of the perfluorooctanoic acid transcript profile in mouse liver: evidence for the involvement of nuclear receptors PPAR alpha and CAR Toxicol Sci 2008 103 46 56 10.1093/toxsci/kfn025 18281256

[B20] Kirstetter P Schuster MB Bereshchenko O Moore S Dvinge H Kurz E Theilgaard-Monch K Mansson R Pedersen TA Pabst T Schrock E Porse BT Jacobsen SEW Bertone P Tenen DG Nerlov C Modeling of C/EBPalpha mutant acute myeloid leukemia reveals a common expression signature of committed myeloid leukemia-initiating cells Cancer Cell 2008 13 4 299 310 10.1016/j.ccr.2008.02.008 18394553

[B21] Kurek D Garinis GA van Doorninck JH Wees J van der Grosveld FG Transcriptome and phenotypic analysis reveals Gata3-dependent signalling pathways in murine hair follicles Development 2007 134 2 261 272 10.1242/dev.02721 17151017

[B22] Sinha S Adler AS Field Y Chang HY Segal E Systematic functional characterization of cis-regulatory motifs in human core promoters Genome Res 2008 18 3 477 488 10.1101/gr.6828808 18256240 PMC2259112

[B23] Warner JB Philippakis AA Jaeger SA He FS Lin J Bulyk ML Systematic identification of mammalian regulatory motifs' target genes and functions Nat Methods 2008 5 4 347 353 18311145 10.1038/nmeth.1188 PMC2708972

[B24] Ho Sui SJ Mortimer JR Arenillas DJ Brumm J Walsh CJ Kennedy BP Wasserman WW oPOSSUM: identification of over-represented transcription factor binding sites in co-expressed genes Nucleic Acids Res 2005 33 10 3154 3164 10.1093/nar/gki624 15933209 PMC1142402

[B25] Sui SJH Fulton DL Arenillas DJ Kwon AT Wasserman WW oPOSSUM: integrated tools for analysis of regulatory motif over-representation Nucleic Acids Res 2007 35 Web Server W245 W252 10.1093/nar/gkm427 17576675 PMC1933229

[B26] Marstrand TT Frellsen J Moltke I Thiim M Valen E Retelska D Krogh A Asap: a framework for over-representation statistics for transcription factor binding sites PLoS ONE 2008 3 2 e1623 10.1371/journal.pone.0001623 18286180 PMC2229843

[B27] Roider HG Kanhere A Manke T Vingron M Predicting transcription factor affnities to DNA from a biophysical model Bioinformatics 2007 23 2 134 141 10.1093/bioinformatics/btl565 17098775

[B28] Hestand M van Galen M Villerius M van Ommen G den Dunnen J 't Hoen P CORE_TF: a user-friendly interface to identify evolutionary conserved transcription factor binding sites in sets of co-regulated genes BMC Bioinformatics 2008 9 495 10.1186/1471-2105-9-495 19036135 PMC2613159

[B29] Portales-Casamar E Arenillas D Lim J Swanson MI Jiang S McCallum A Kirov S Wasserman WW The PAZAR database of gene regulatory information coupled to the ORCA toolkit for the study of regulatory sequences Nucleic Acids Res 2009 37 Database D54 60 10.1093/nar/gkn783 18971253 PMC2686574

[B30] Karanam S Moreno CS CONFAC: automated application of comparative genomic promoter analysis to DNA microarray datasets Nucleic Acids Res 2004 32 Web Server W475 84 10.1093/nar/gkh353 15215433 PMC441491

[B31] Gotea V Ovcharenko I DiRE: identifying distant regulatory elements of co-expressed genes Nucleic Acids Res 2008 36 Web Server W133 9 10.1093/nar/gkn300 18487623 PMC2447744

[B32] Kim SY Kim Y Genome-wide prediction of transcriptional regulatory elements of human promoters using gene expression and promoter analysis data BMC Bioinformatics 2006 7 330 10.1186/1471-2105-7-330 16817975 PMC1586028

[B33] Kankainen M Holm L POCO: discovery of regulatory patterns from promoters of oppositely expressed gene sets Nucleic Acids Res 2005 33 Web Server W427 31 10.1093/nar/gki467 15980504 PMC1160228

[B34] Mrowka R Bluthgen N Fahling M Seed-based systematic discovery of specific transcription factor target genes FEBS J 2008 275 12 3178 3192 10.1111/j.1742-4658.2008.06471.x 18485006

[B35] Aerts S Thijs G Coessens B Staes M Moreau Y De Moor B Toucan: deciphering the cis-regulatory logic of coregulated genes Nucleic Acids Res 2003 31 6 1753 1764 10.1093/nar/gkg268 12626717 PMC152870

[B36] Reddy TE DeLisi C Shakhnovich BE Binding site graphs: a new graph theoretical framework for prediction of transcription factor binding sites PLoS Comput Biol 2007 3 5 e90 10.1371/journal.pcbi.0030090 17500587 PMC1866359

[B37] Gertz J Siggia E Cohen B Analysis of combinatorial cis-regulation in synthetic and genomic promoters Nature 2008 19029883 10.1038/nature07521 PMC2677908

[B38] Lee TI Rinaldi NJ Robert F Odom DT Bar-Joseph Z Gerber GK Hannett NM Harbison CT Thompson CM Simon I Zeitlinger J Jennings EG Murray HL Gordon DB Ren B Wyrick JJ Tagne JB Volkert TL Fraenkel E Gifford DK Young RA Transcriptional regulatory networks in Saccharomyces cerevisiae Science 2002 298 5594 799 804 10.1126/science.1075090 12399584

[B39] Gertz J Cohen BA Environment-specific combinatorial cis-regulation in synthetic promoters Mol Syst Biol 2009 5 244 10.1038/msb.2009.1 19225457 PMC2657533

[B40] Parkinson H Kapushesky M Kolesnikov N Rustici G Shojatalab M Abeygunawardena N Berube H Dylag M Emam I Farne A Holloway E Lukk M Malone J Mani R Pilicheva E Rayner TF Rezwan F Sharma A Williams E Bradley XZ Adamusiak T Brandizi M Burdett T Coulson R Krestyaninova M Kurnosov P Maguire E Neogi SG Rocca-Serra P Sansone SA Sklyar N Zhao M Sarkans U Brazma A ArrayExpress update-from an archive of functional genomics experiments to the atlas of gene expression Nucleic Acids Res 2009 37 Database D868 72 10.1093/nar/gkn889 19015125 PMC2686529

[B41] Sandelin A Wasserman WW Constrained binding site diversity within families of transcription factors enhances pattern discovery bioinformatics J Mol Biol 2004 338 2 207 15 10.1016/j.jmb.2004.02.048 15066426

[B42] Vlieghe D Sandelin A De Bleser PJ Vleminckx K Wasserman WW van Roy F Lenhard B A new generation of JASPAR, the open-access repository for transcription factor binding site profiles Nucleic Acids Res 2006 34 Database D95 7 10.1093/nar/gkj115 16381983 PMC1347477

[B43] Pavesi G Mereghetti P Mauri G Pesole G Weeder Web: discovery of transcription factor binding sites in a set of sequences from co-regulated genes Nucleic Acids Res 2004 32 Web Server W199 203 10.1093/nar/gkh465 15215380 PMC441603

[B44] Sandelin A Höglund A Lenhard B Wasserman WW Integrated analysis of yeast regulatory sequences for biologically linked clusters of genes Funct Integr Genomics 2003 3 3 125 134 10.1007/s10142-003-0086-6 12827523

[B45] Irizarry RA Hobbs B Collin F Beazer-Barclay YD Antonellis KJ Scherf U Speed TP Exploration, normalization, and summaries of high density oligonucleotide array probe level data Biostatistics 2003 4 2 249 264 10.1093/biostatistics/4.2.249 12925520

[B46] Tusher VG Tibshirani R Chu G Significance analysis of microarrays applied to the ionizing radiation response Proc Natl Acad Sci USA 2001 98 9 5116 5121 10.1073/pnas.091062498 11309499 PMC33173

[B47] Beissbarth T Fellenberg K Brors B Arribas-Prat R Boer J Hauser NC Scheideler M Hoheisel JD Schütz G Poustka A Vingron M Processing and quality control of DNA array hybridization data Bioinformatics 2000 16 11 1014 22 10.1093/bioinformatics/16.11.1014 11159313

[B48] Storey JD Tibshirani R Statistical significance for genomewide studies Proc Natl Acad Sci USA 2000 100 16 1014 22 10.1073/pnas.1530509100 PMC170937 12883005

[B49] Vingron M Brazma A Coulson R van Helden J Manke T Palin K Sand O Ukkonen E Integrating sequence, evolution and functional genomics in regulatory genomics Genome Biol 2009 10 202 10.1186/gb-2009-10-1-202 19226437 PMC2687781

[B50] Tompa M Li N Bailey TL Church GM Moor BD Eskin E Favorov AV Frith MC Fu Y Kent WJ Makeev VJ Mironov AA Noble WS Pavesi G Pesole G Régnier M Simonis N Sinha S Thijs G van Helden J Vandenbogaert M Weng Z Workman C Ye C Zhu Z Assessing computational tools for the discovery of transcription factor binding sites Nat Biotechnol 2005 23 137 144 10.1038/nbt1053 15637633

[B51] Klinge CM Estrogen receptor interaction with estrogen response elements Nucleic Acids Res 2001 29 14 2905 2919 10.1093/nar/29.14.2905 11452016 PMC55815

[B52] Morgan XC Ni S Miranker DP Iyer VR Predicting combinatorial binding of transcription factors to regulatory elements in the human genome by association rule mining BMC Bioinformatics 2007 8 445 10.1186/1471-2105-8-445 18005433 PMC2211755

[B53] Wang RS Zhang XS Chen L Inferring transcriptional interactions and regulator activities from experimental data Mol Cells 2007 24 3 307 315 18182844

[B54] Saunthararajah Y Boccuni P Nucifora G Combinatorial action of RUNX1 and PU.1 in the regulation of hematopoiesis Crit Rev Eukaryot Gene Expr 2006 16 2 183 192 16749897 10.1615/critreveukargeneexpr.v16.i2.60

[B55] Glass CK Ogawa S Combinatorial roles of nuclear receptors in inflammation and immunity Nat Rev Immunol 2006 6 44 55 10.1038/nri1748 16493426

[B56] Messenguy F Dubois E Role of MADS box proteins and their cofactors in combinatorial control of gene expression and cell development Gene 2003 316 1 21 10.1016/S0378-1119(03)00747-9 14563547

[B57] Landry JR Bonadies N Kinston S Knezevic K Wilson NK Oram SH Janes M Piltz S Hammett M Carter J Hamilton T Donaldson IJ Lacaud G Frampton J Follows G Kouskoff V Göttgens B Expression of the leukemia oncogene Lmo2 is controlled by an array of tissue-specific elements dispersed over 100 kb and bound by Tal1/Lmo2, Ets, and Gata factors Blood 2009 113 23 5783 92 10.1182/blood-2008-11-187757 19171877

[B58] Wall L deBoer E Grosveld F The human beta-globin gene 3' enhancer contains multiple binding sites for an erythroid-specific protein Genes Dev 1988 2 9 1089 100 10.1101/gad.2.9.1089 2461328

[B59] Grass JA Boyer ME Pal S Wu J Weiss MJ Bresnick EH GATA-1-dependent transcriptional repression of GATA-2 via disruption of positive autoregulation and domain-wide chromatin remodeling Proc Natl Acad Sci USA 2003 100 15 8811 6 10.1073/pnas.1432147100 12857954 PMC166395

[B60] Ruf IK Rhyne PW Yang H Borza CM Hutt-Fletcher LM Cleveland JL Sample JT EBV regulates c-MYC, apoptosis, and tumorigenicity in Burkitt's lymphoma Curr Top Microbiol Immunol 2001 258 153 160 11443860 10.1007/978-3-642-56515-1_10

[B61] Lüscher B Function and regulation of the transcription factors of the Myc/Max/Mad network Gene 2001 277 1-2 1 14 10.1016/S0378-1119(01)00697-7 11602341

[B62] D'haeseleer P How does DNA sequence motif discovery work? Nat Biotechnol 2006 24 8 959 961 10.1038/nbt0806-959 16900144

