# Supplementary material for: A computational evaluation of over-representation of regulatory motifs in the promoter regions of differentially expressed genes
Source: BMC Bioinformatics. 2010 May 20;11:267. doi: 10.1186/1471-2105-11-267 (PMC3098066; doi:10.1186/1471-2105-11-267)
Supplement: Additional file 1 — Assessment of microarray quality with scatter plots and p-value frequency histograms. To eliminate those microarrays with low quality, we used two methods for quality evaluation. The first one was based on scatter plots, in which the averaged normalized expression value of manipulated hybrids and control hybrids were plotted. Another methods was histograms of q-value frequency distributions, predicted by SAM. We manually checked those distribution and selected reasonable experiments for differential expression analysis. [file 1471-2105-11-267-S1.PDF]

<table>
<tr><th>Experiments</th><th>TF</th><th>scatter plots</th><th>p_value histogram</th><th>Used in next step?</th><th>Remark</th></tr>
<tr><td>E-GEOD-10954</td><td>cmyc</td><td>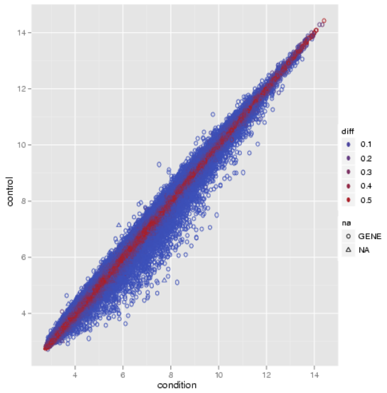
</td><td>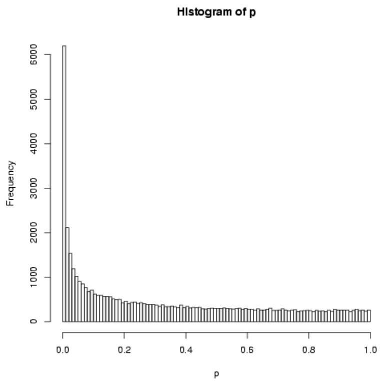
</td><td>yes</td><td>Reasonable distribution</td></tr>
</table>

| | | | | | |
|---|---|---|---|---|---|
| E-GEOD-11039 | e2f2 | 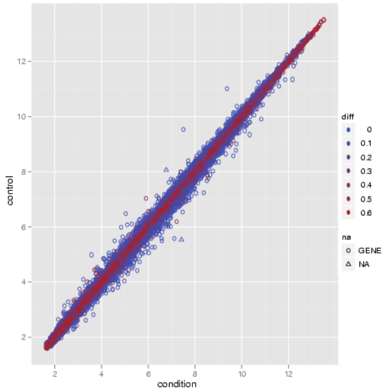 | 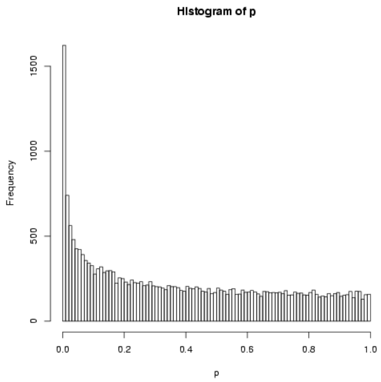 | yes | Reasonable distribution |
| E-GEOD-11352 | estrogen | 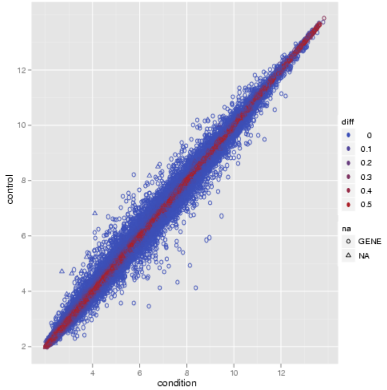 | 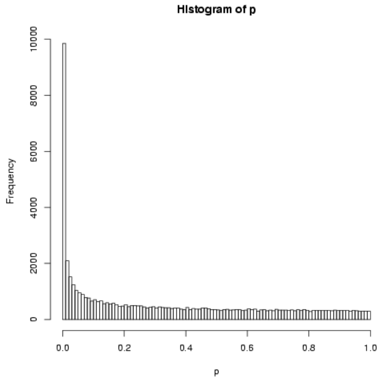 | yes | Reasonable distribution |

<table>
<tr>
<td>E-GEOD-11557</td>
<td>evi1</td>
<td>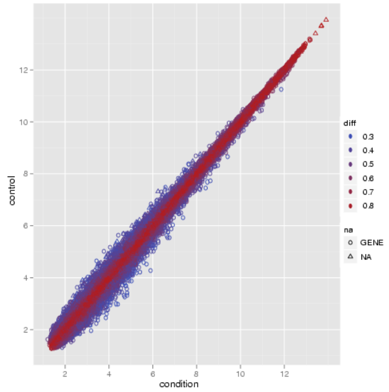
</td>
<td>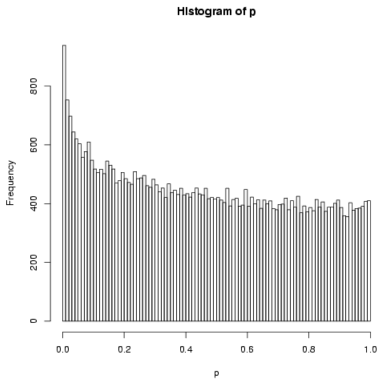
</td>
<td>yes</td>
<td>Reasonable distribution</td>
</tr>
<tr>
<td>E-GEOD-11809</td>
<td>irf1</td>
<td>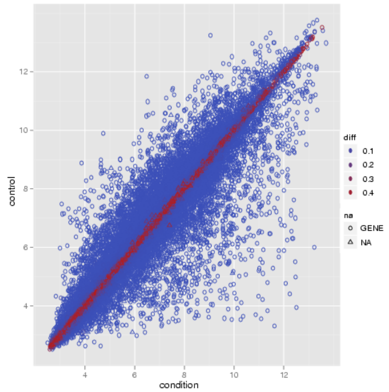
</td>
<td>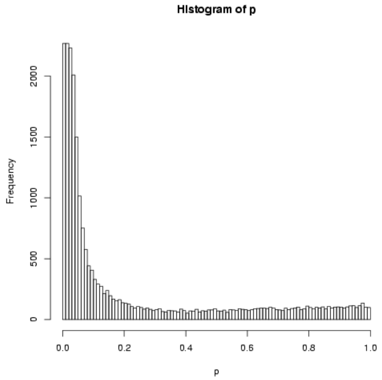
</td>
<td>yes</td>
<td>Reasonable distribution</td>
</tr>
</table>

<table>
<tr><td></td><td></td><td></td><td></td><td></td><td></td></tr>
<tr>
<td>E-GEOD-11836</td>
<td>nkx31</td>
<td>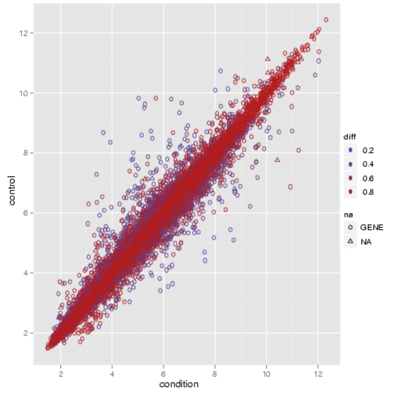
</td>
<td>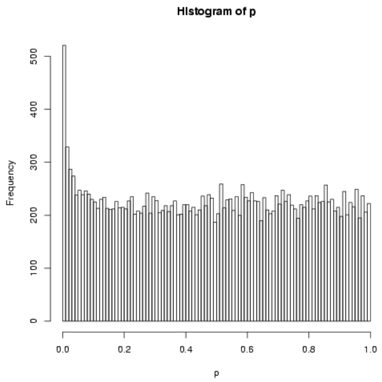
</td>
<td>yes</td>
<td>Reasonable distribution</td>
</tr>
<tr>
<td>E-GEOD-2060</td>
<td>creb</td>
<td>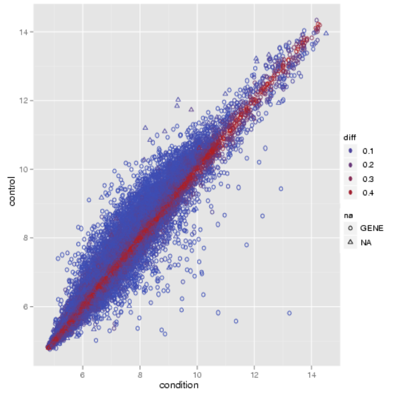
</td>
<td>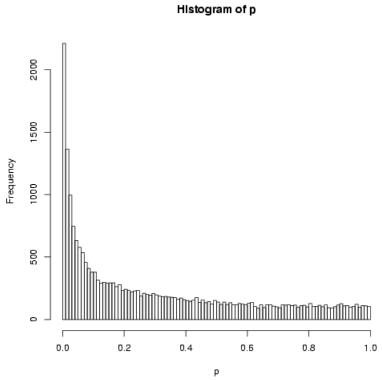
</td>
<td>yes</td>
<td>Reasonable distribution</td>
</tr>
</table>

| | | | | | |
|---|---|---|---|---|---|
| E-GEOD-2192 | pparg | 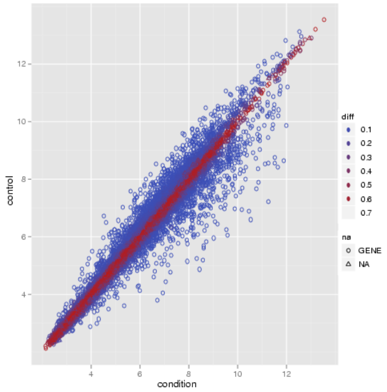  | 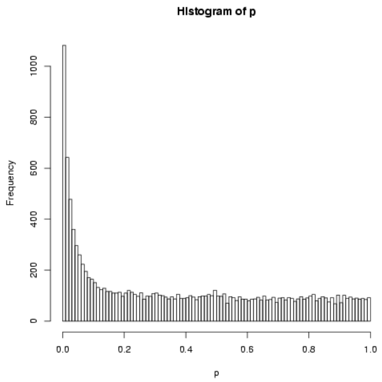  | yes | Reasonable distribution |
| E-GEOD-2527 | gata1 | 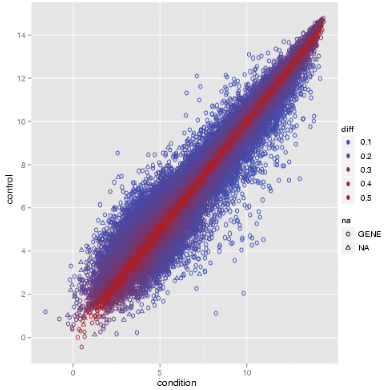  | 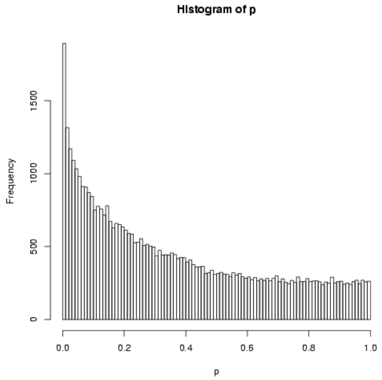  | yes | Reasonable distribution |

| | | | | | |
|---|---|---|---|---|---|
| E-GEOD-2624 | nfkb | 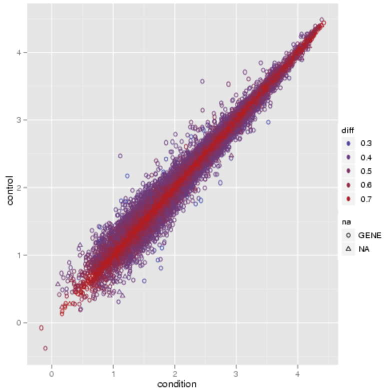 | 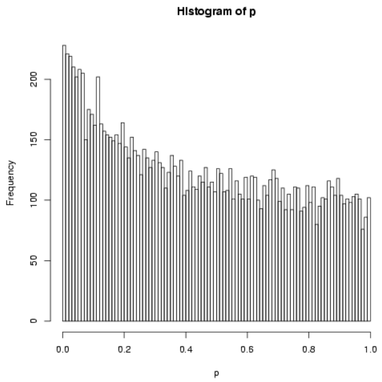 | yes | Reasonable distribution |
| E-GEOD-2815 | cmyb | 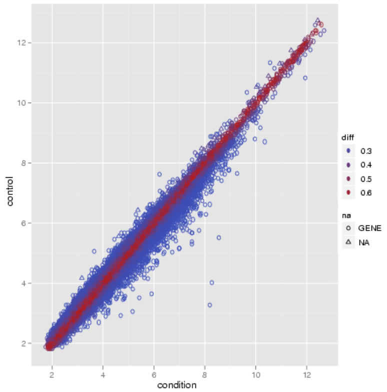 | 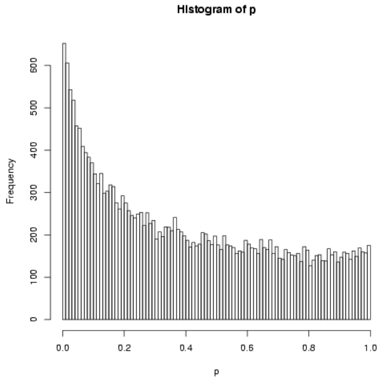 | yes | Reasonable distribution |

| | | | | | |
|---|---|---|---|---|---|
| E-GEOD-3116 | hnf4 | 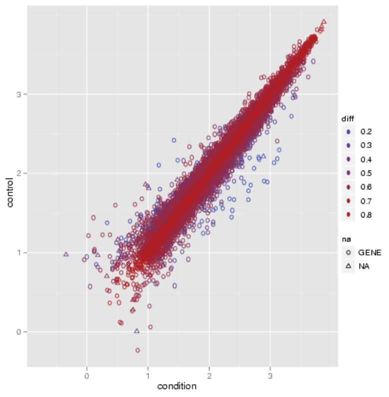 | 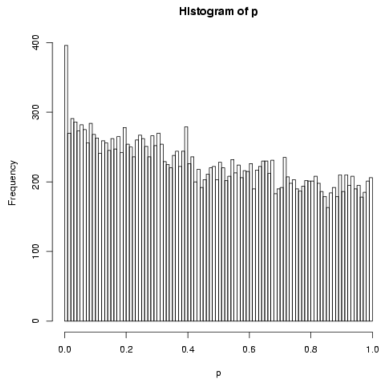 | yes | Reasonable distribution |
| E-GEOD-3126 | hnf4a | 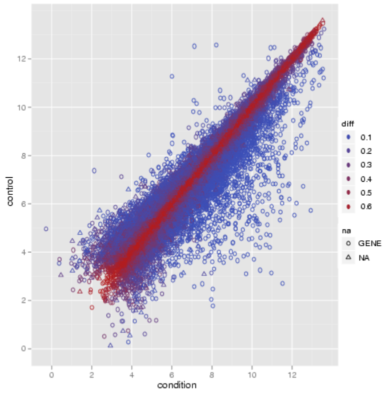 | 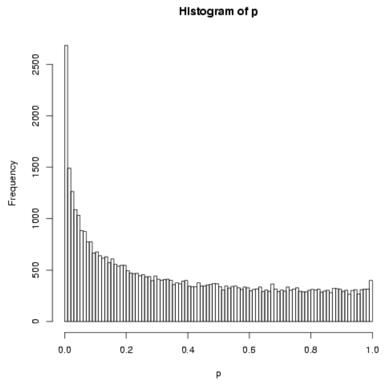 | yes | Reasonable distribution |

| | | | | | |
|---|---|---|---|---|---|
| E-GEOD-3244 | myod-p53 | 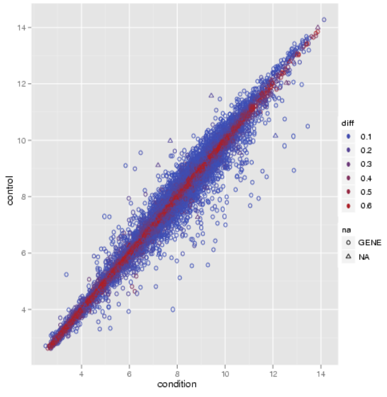 | 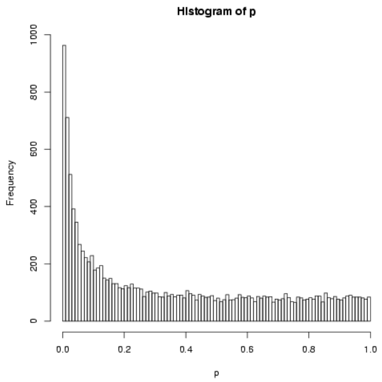 | yes | Reasonable distribution |
| E-GEOD-5424 | fox | 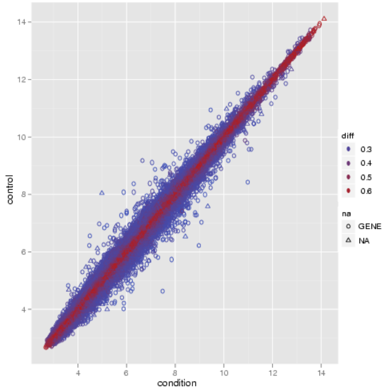 | 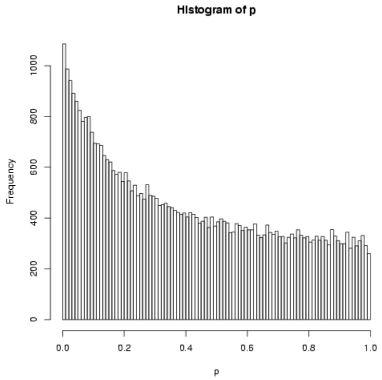 | yes | Reasonable distribution |

| | | | | | |
|---|---|---|---|---|---|
| E-GEOD-5475 | PPARI | 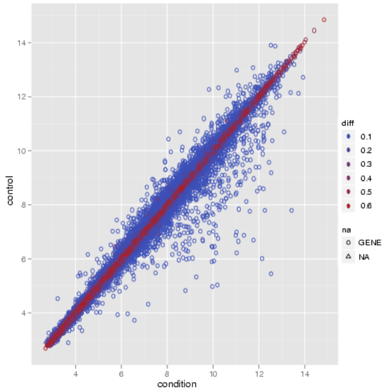 | 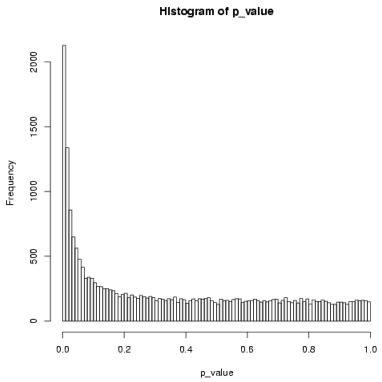 | yes | Reasonable distribution |
| E-GEOD-5800 | IRF6 | 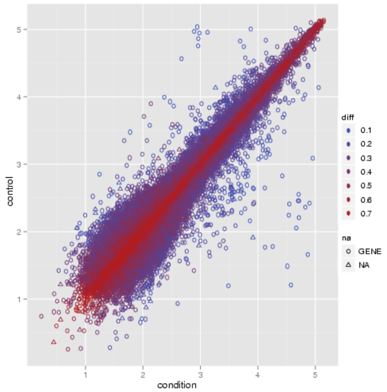 | 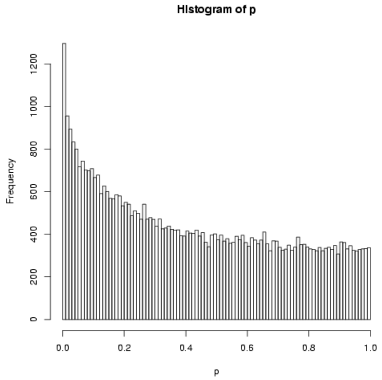 | yes | Reasonable distribution |

| | | | | | |
|---|---|---|---|---|---|
| E-GEOD-5823 | myc | 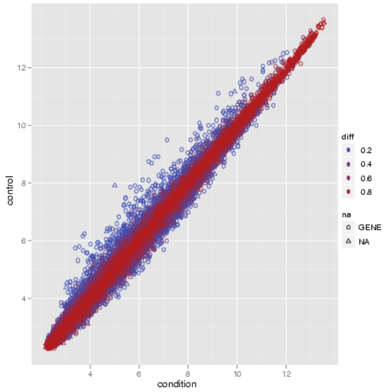  | 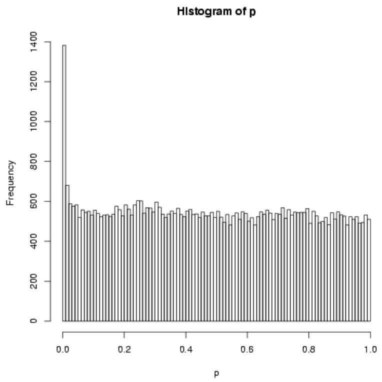  | yes | Reasonable distribution |
| E-GEOD-590 | usf1 | 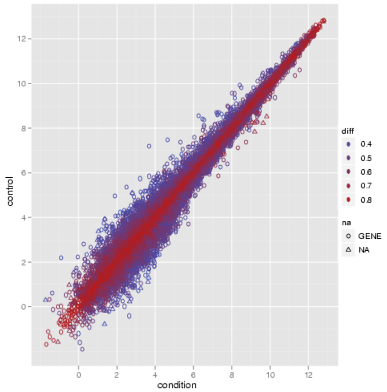  | 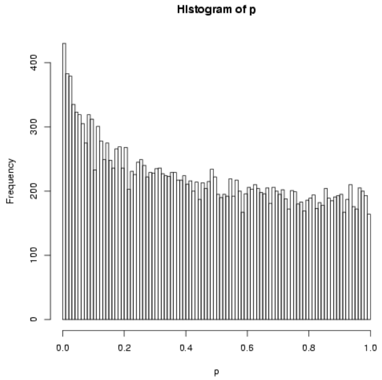  | yes | Reasonable distribution |

| | | | | | |
|---|---|---|---|---|---|
| E-GEOD-6077 | mycn | 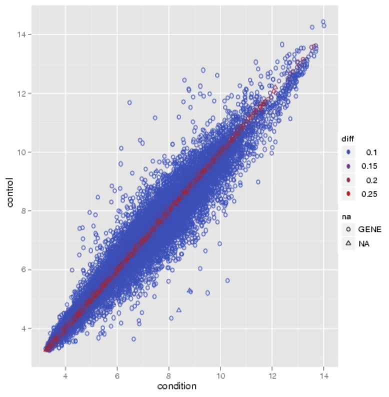 | 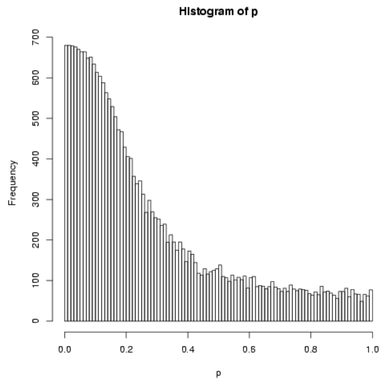 | yes | Reasonable distribution |
| E-GEOD-6487 | myod | 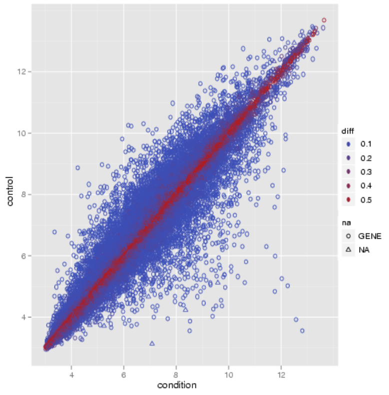 | 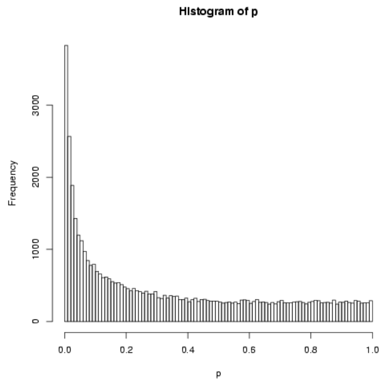 | yes | Reasonable distribution |

| | | | | | |
|---|---|---|---|---|---|
| E-GEOD-6846 | stat | 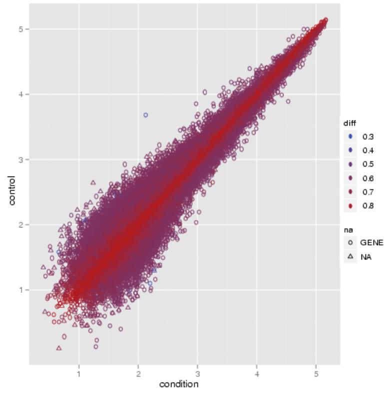 | 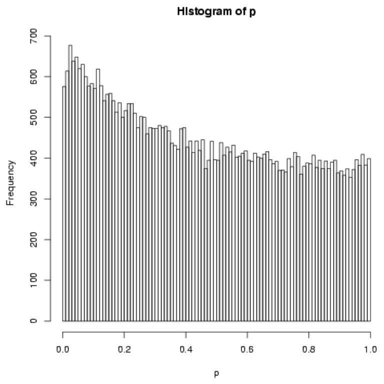 | yes | Reasonable distribution |
| E-GEOD-7137 | KLF | 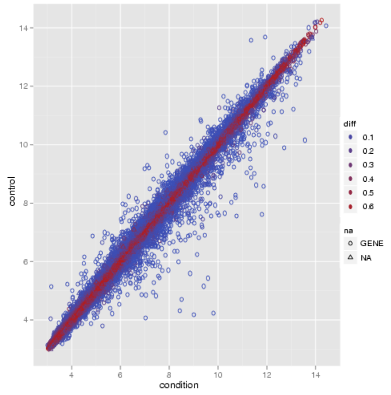 | 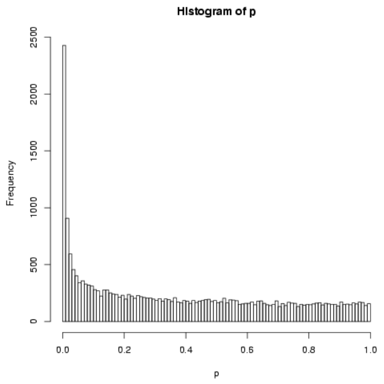 | yes | Reasonable distribution |

| | | | | | |
|---|---|---|---|---|---|
| E-GEOD-7219 | NFkappa[illegible] | 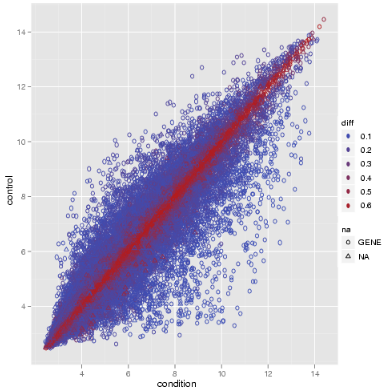 | 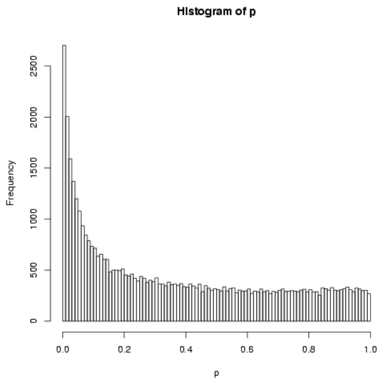 | yes | Reasonable distribution |
| E-GEOD-7223 | bzip | 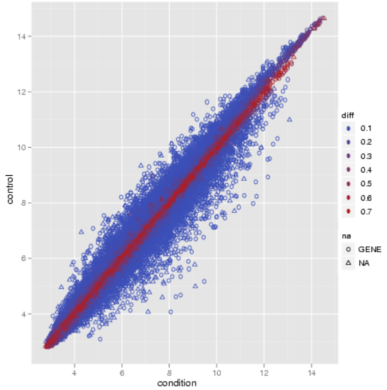 | 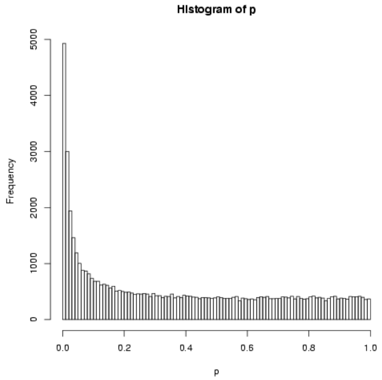 | yes | Reasonable distribution |

| | | | | | |
|---|---|---|---|---|---|
| E-GEOD-7835 | hif1 | 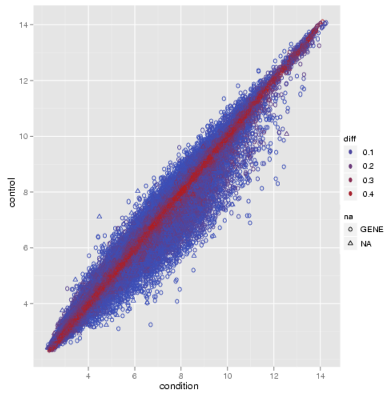 | 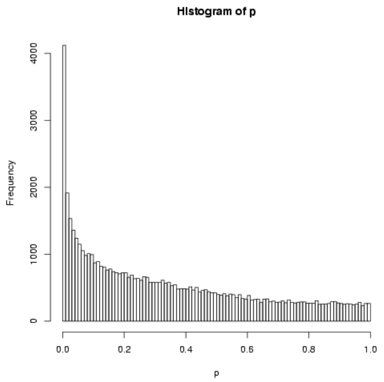 | yes | Reasonable distribution |
| E-GEOD-8943 | foxq | 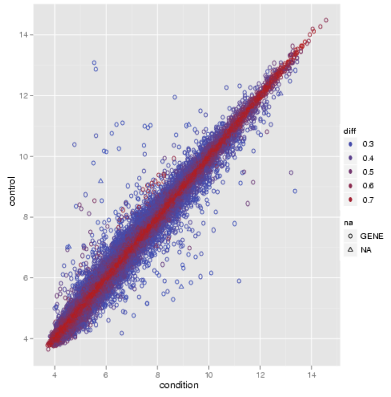 | 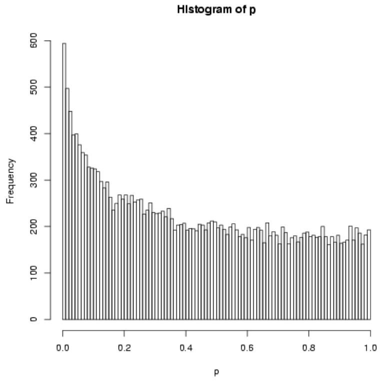 | yes | Reasonable distribution |

| | | | | | |
|---|---|---|---|---|---|
| E-GEOD-9786 | PPARa | 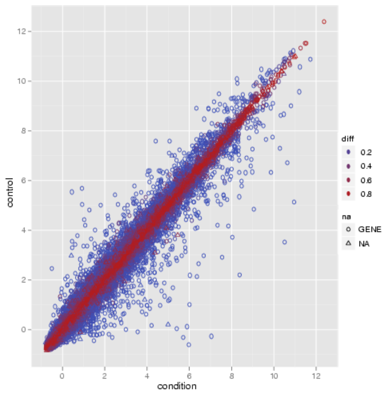 | 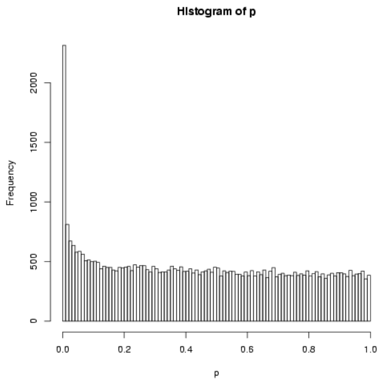 | yes | Reasonable distribution |
| E-MEXP-1413 | e2f2 | 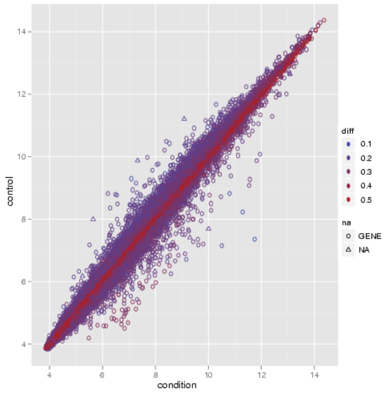 | 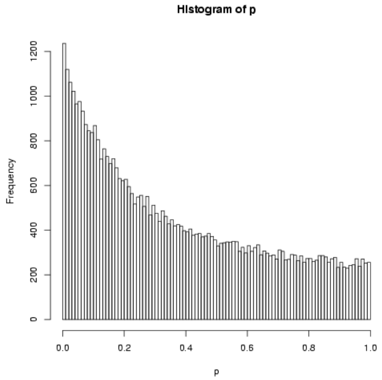 | yes | Reasonable distribution |

| | | | | | |
|---|---|---|---|---|---|
| E-MEXP-1444 | cebpa | 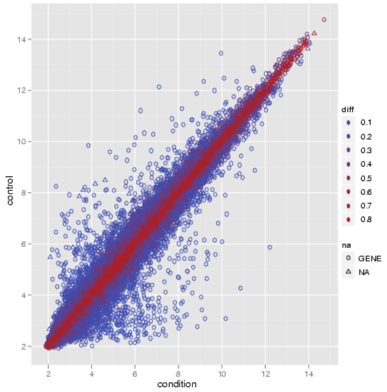  | 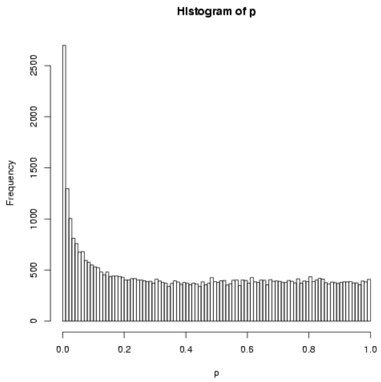  | yes | Reasonable distribution |
| E-MEXP-634 | gata | 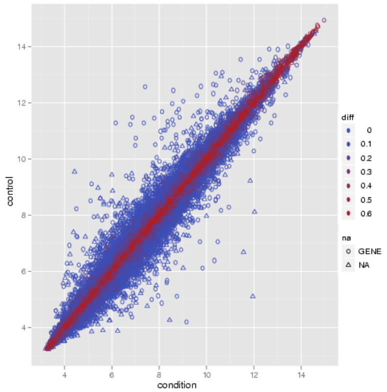  | 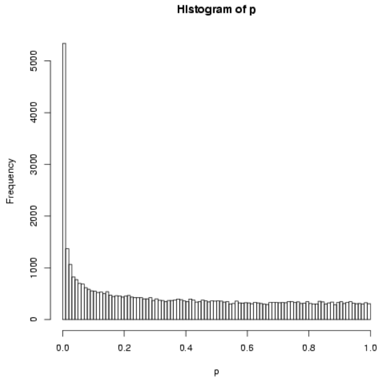  | yes | Reasonable distribution |

| | | | | | |
|---|---|---|---|---|---|
| E-MEXP-871 | hmga2 | 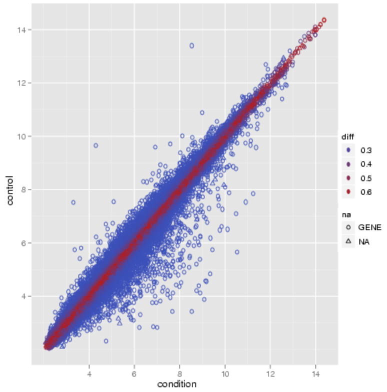 | 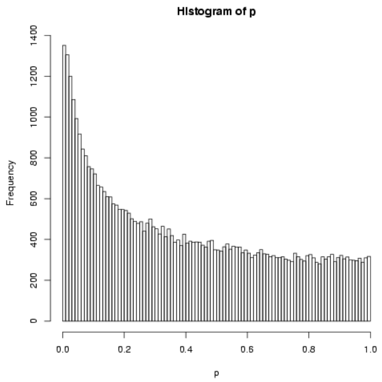 | yes | Reasonable distribution |
| E-TABM-43 | tp53 | 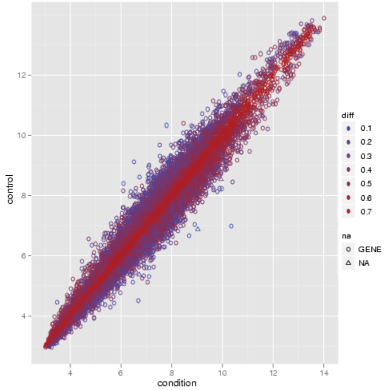 | 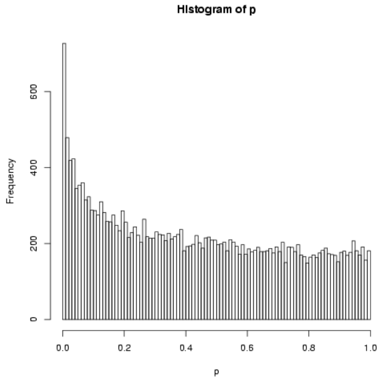 | yes | Reasonable distribution |

| | | | | | |
|---|---|---|---|---|---|
| E-CBIL-21 | hnf1a | 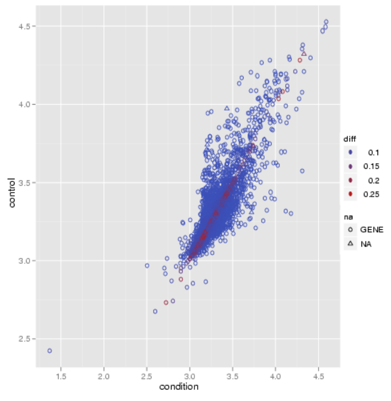 | 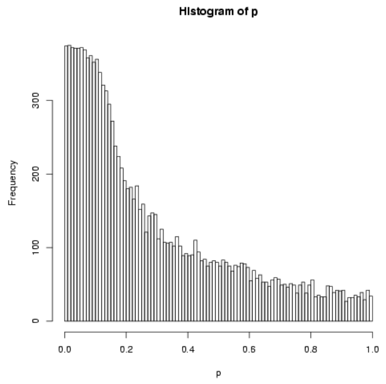 | no | unreasonable scatter plot |
| E-GEOD-109 | hoxa | 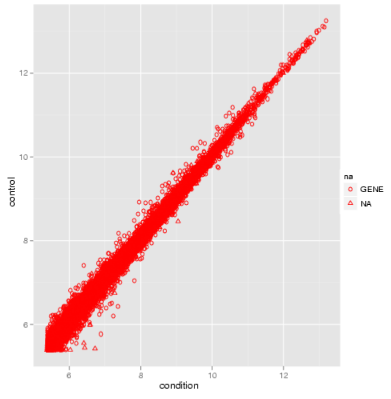 | 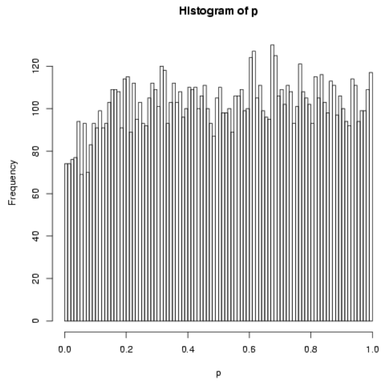 | no | both unreasonable |

| | | | | | |
|---|---|---|---|---|---|
| E-GEOD-11165 | gata6 | 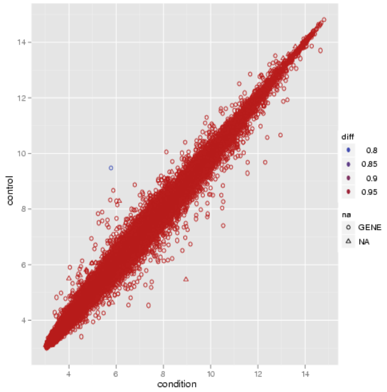  | 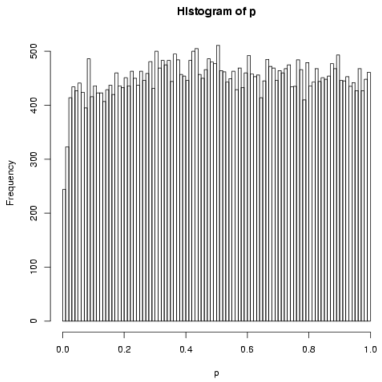  | no | unreasonable p-value distribution |
| E-GEOD-11914 | sox4 | 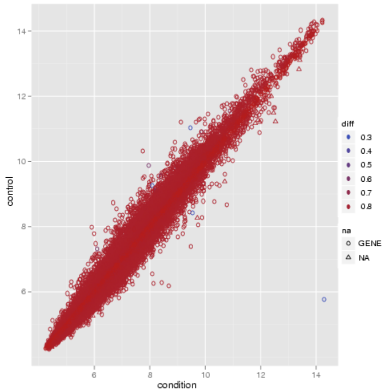  | 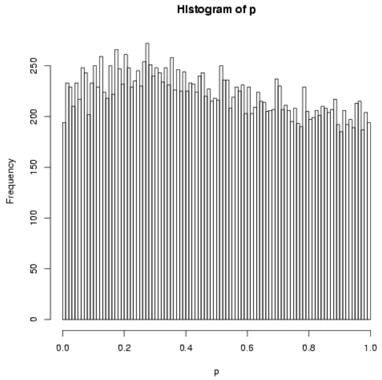  | no | unreasonable p-value distribution |

| | | | | | |
|---|---|---|---|---|---|
| E-GEOD-1949 | srf | 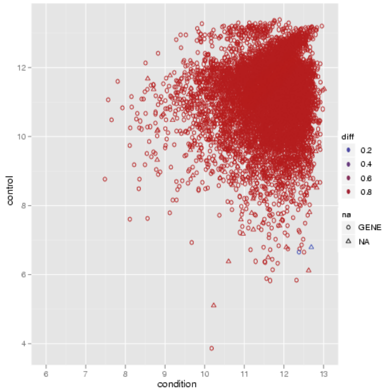 | 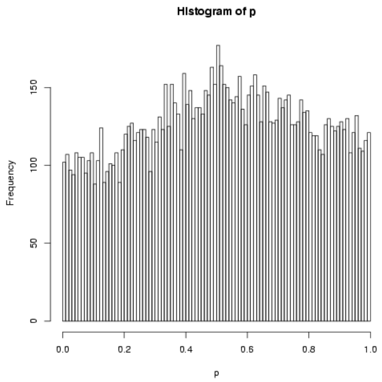 | no | both unreasonable |
| E-GEOD-2222 | fox | 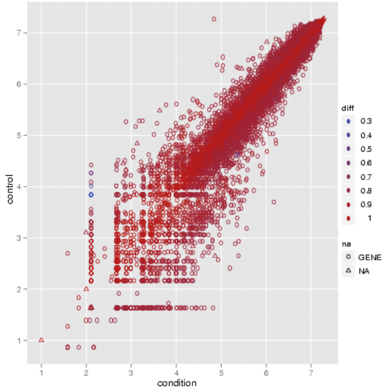 | 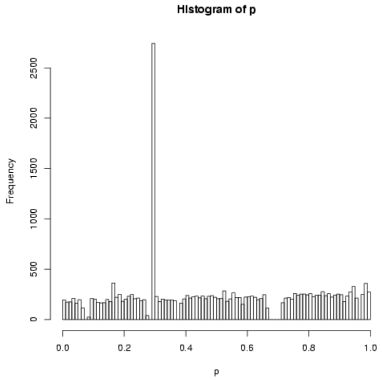 | no | both unreasonable |

| | | | | | |
|---|---|---|---|---|---|
| E-GEOD-2241 | hoxa5 | 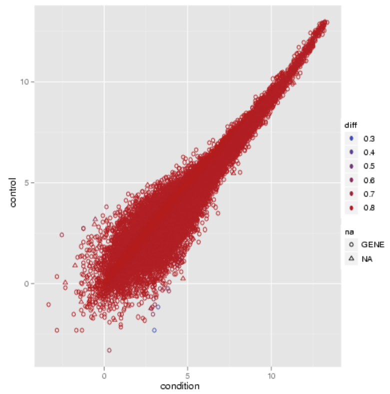 | 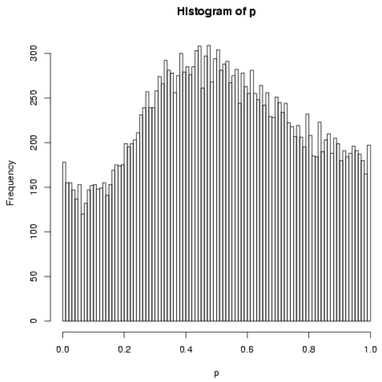 | no | both unreasonable |
| E-GEOD-2259 | ar | 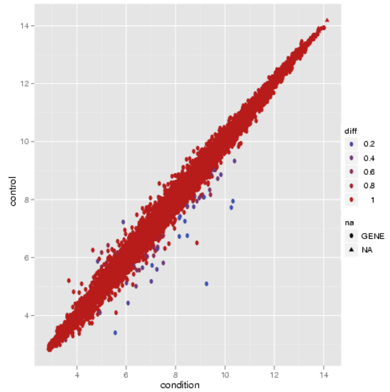 | 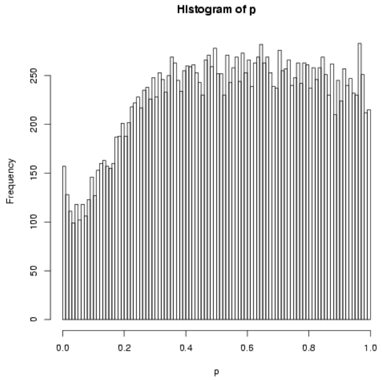 | no | unreasonable p-value distribution |

| | | | | | |
|---|---|---|---|---|---|
| E-GEOD-2421 | vdr | 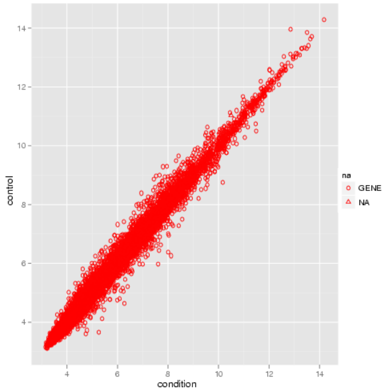 | 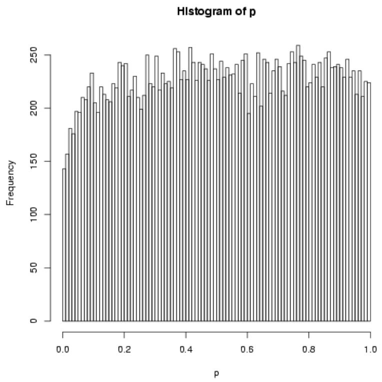 | no | unreasonable p-value distribution |
| E-GEOD-2567 | smad4 | 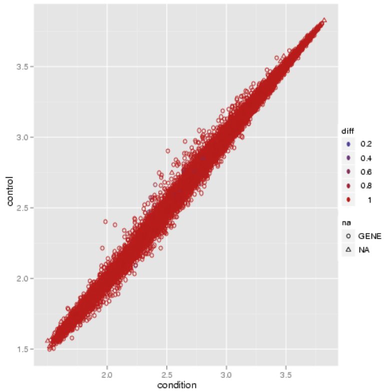 | 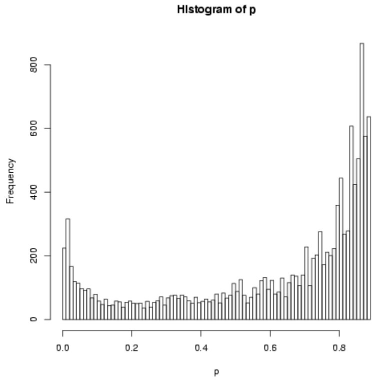 | no | unreasonable p-value distribution |

| | | | | | |
|---|---|---|---|---|---|
| E-GEOD-2592 | Runx | 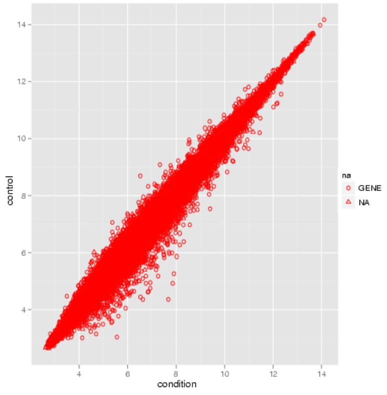  | 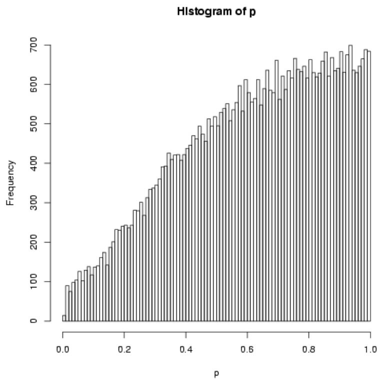  | no | both unreasonable |
| E-GEOD-2699 | ppar | 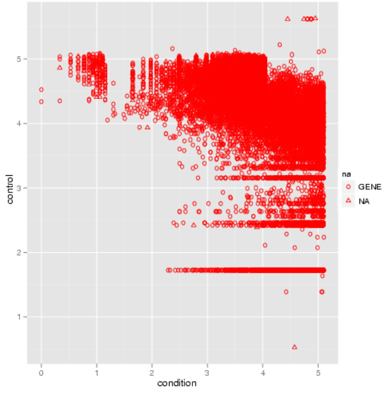  | 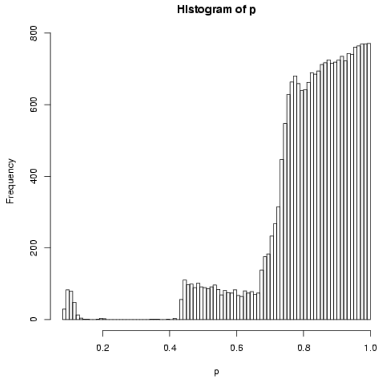  | no | both unreasonable |

| | | | | | |
|---|---|---|---|---|---|
| E-GEOD-2700 | hnf4a | 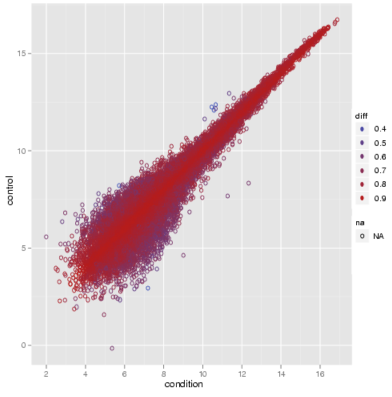  | 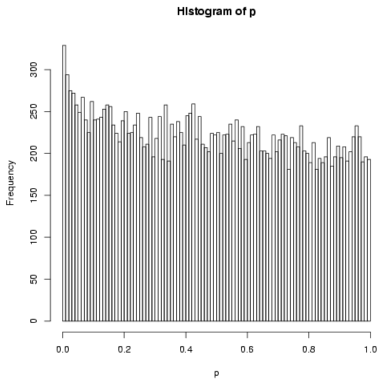  | no | both unreasonable |
| E-GEOD-2816 | cmyb | 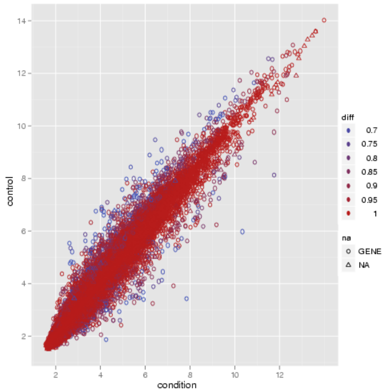  | 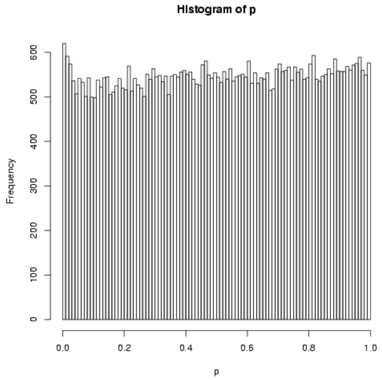  | no | unreasonable p-value distribution |

| | | | | | |
|---|---|---|---|---|---|
| E-GEOD-3124 | hnf4 | 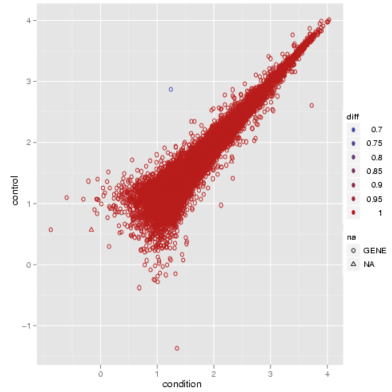 | 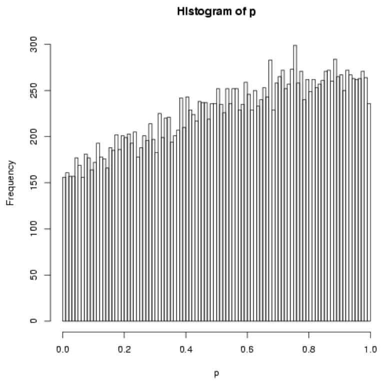 | no | both unreasonable |
| E-GEOD-3181 | srf | 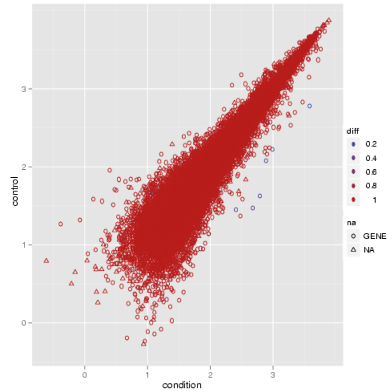 | 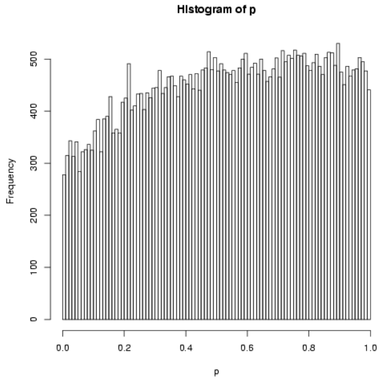 | no | both unreasonable |

| | | | | | |
|---|---|---|---|---|---|
| E-GEOD-3245 | myod-myf5 | 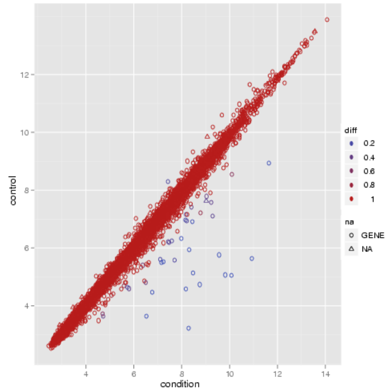 | 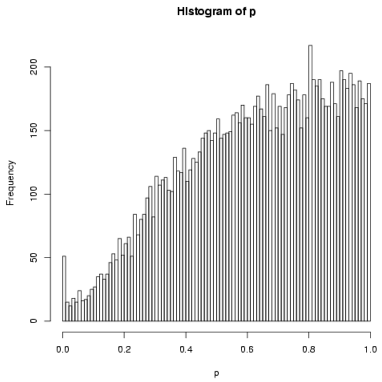 | no | unreasonable p-value distribution |
| E-GEOD-3308 | hnf1b | 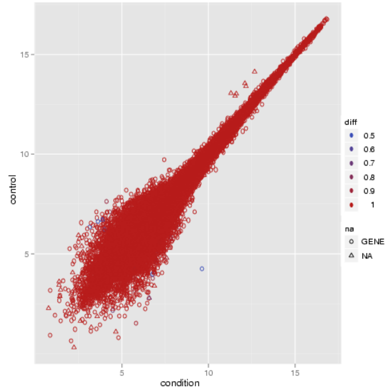 | 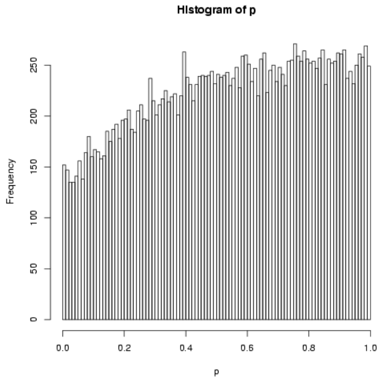 | no | both unreasonable |

| | | | | | |
|---|---|---|---|---|---|
| E-GEOD-3965 | creb | 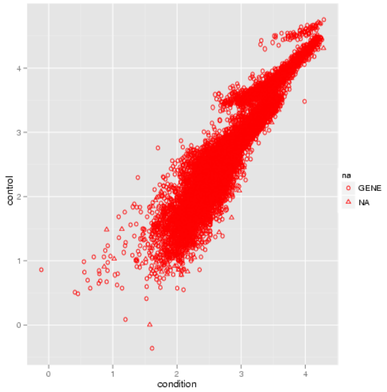 | 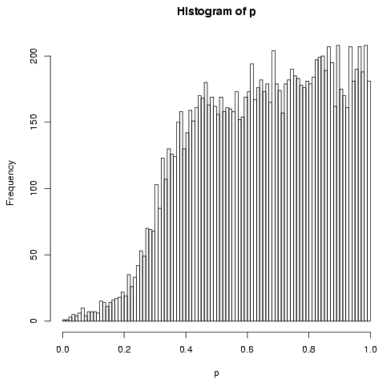 | no | both unreasonable |
| E-GEOD-4225 | sox6 | 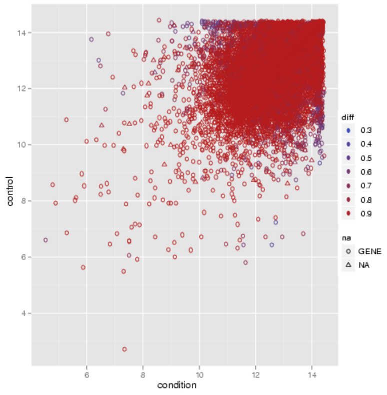 | 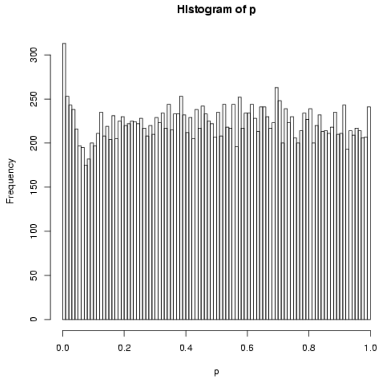 | no | both unreasonable |

<table>
<tr>
<td>E-GEOD-4938</td>
<td>gfi1</td>
<td>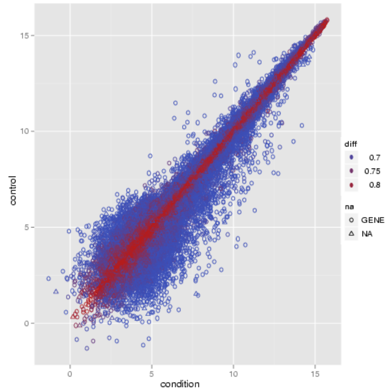
</td>
<td>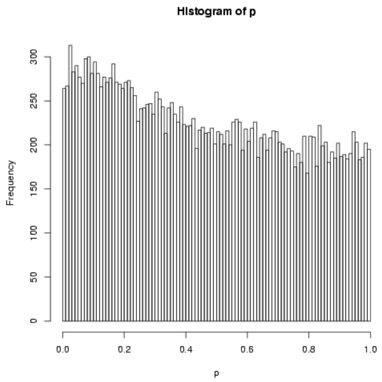
</td>
<td>no</td>
<td>unreasonable p-value distribution</td>
</tr>
<tr>
<td>E-GEOD-495</td>
<td>nrf2</td>
<td>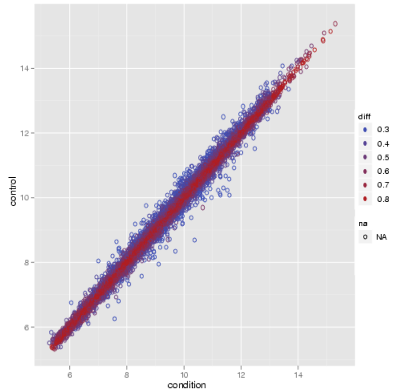
</td>
<td>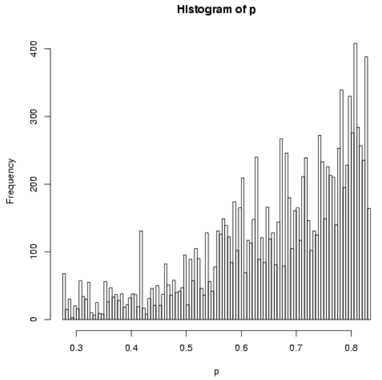
</td>
<td>no</td>
<td>unreasonable p-value distribution</td>
</tr>
</table>

| | | | | | |
|---|---|---|---|---|---|
| E-GEOD-498 | e2f1 | 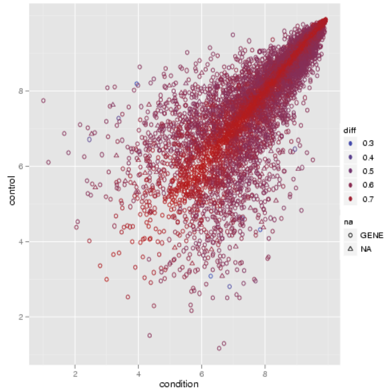  | 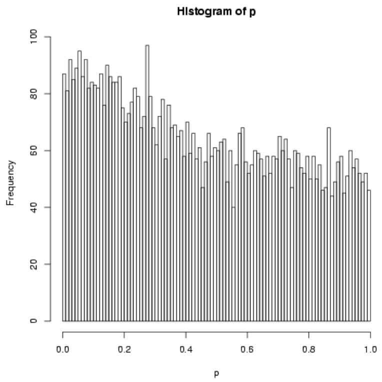  | no | both unreasonable |
| E-GEOD-528 | nkx25 | 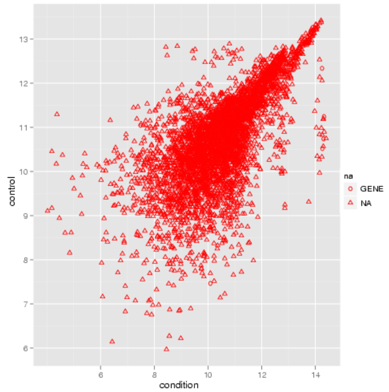  | 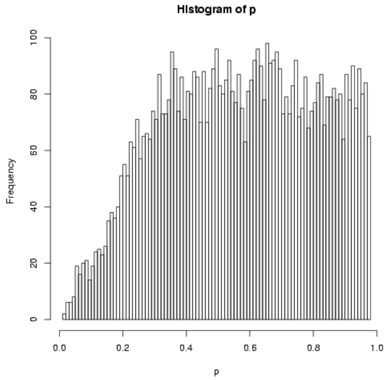  | no | both unreasonable |

| | | | | | |
|---|---|---|---|---|---|
| E-GEOD-5319 | sry | 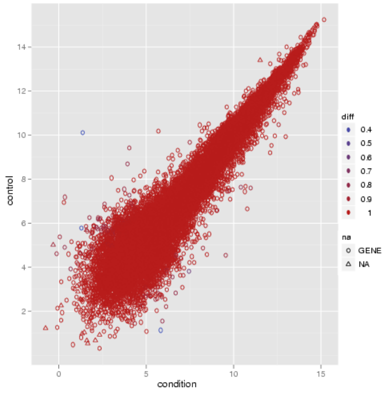 | 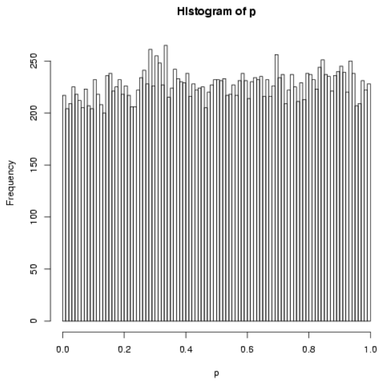 | no | unreasonable p-value distribution |
| E-GEOD-5500 | gata4 | 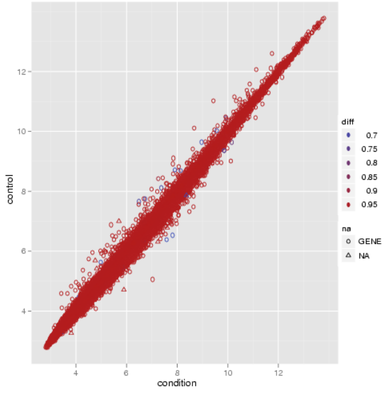 | 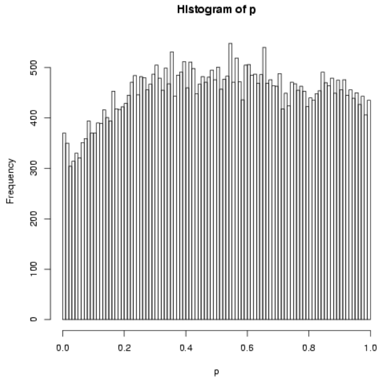 | no | unreasonable p-value distribution |

| | | | | | |
|---|---|---|---|---|---|
| E-GEOD-5679 | pparg | 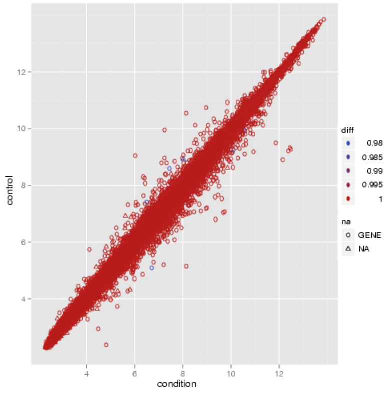 | 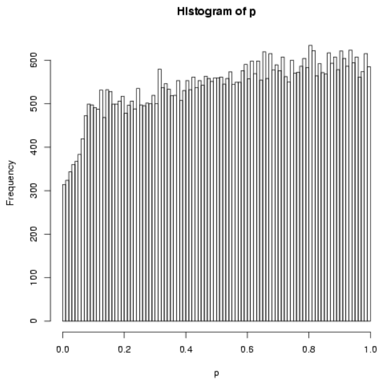 | no | unreasonable p-value distribution |
| E-GEOD-6875 | foxp | 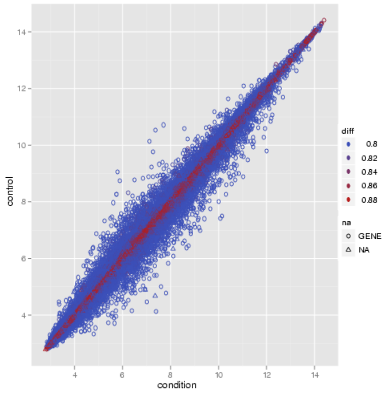 | 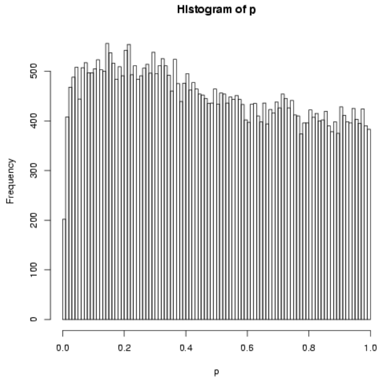 | no | unreasonable p-value distribution |

| | | | | | |
|---|---|---|---|---|---|
| E-GEOD-7101 | p53 | 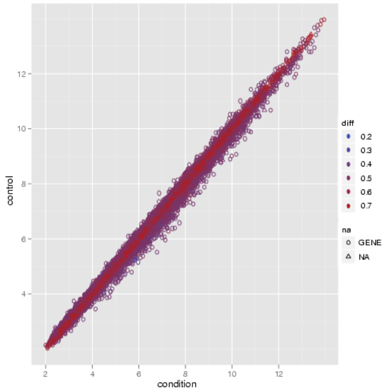  | 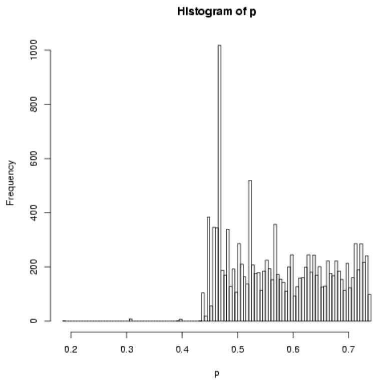  | no | unreasonable p-value distribution |
| E-GEOD-7412 | srf | 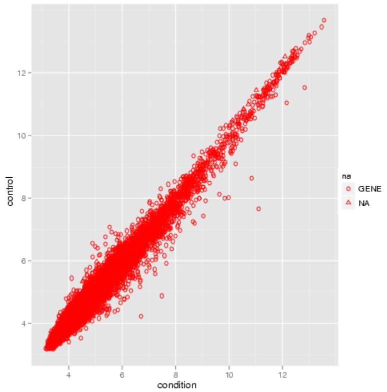  | 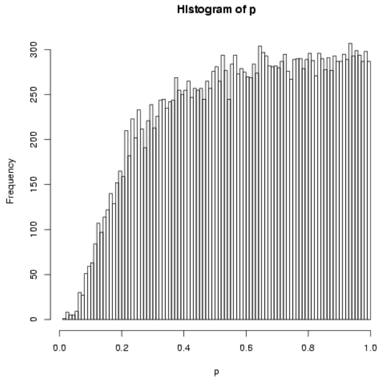  | no | unreasonable p-value distribution |

| | | | | | |
|---|---|---|---|---|---|
| E-GEOD-78 | nkx25 | 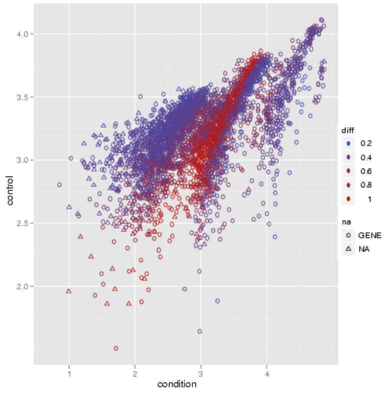  | 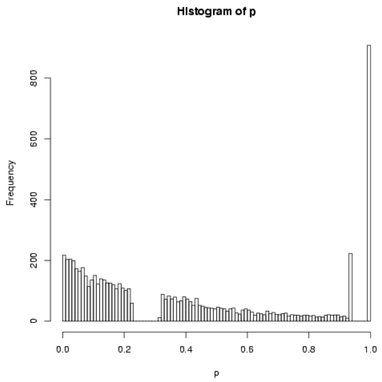  | no | both unreasonable |
| E-GEOD-7810 | nrf2 | 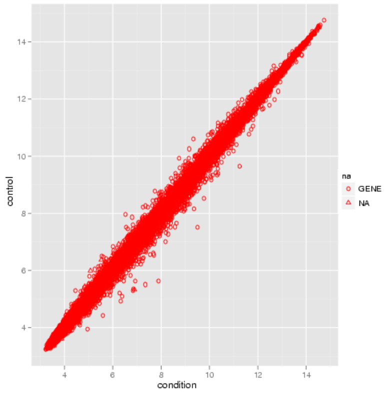  | 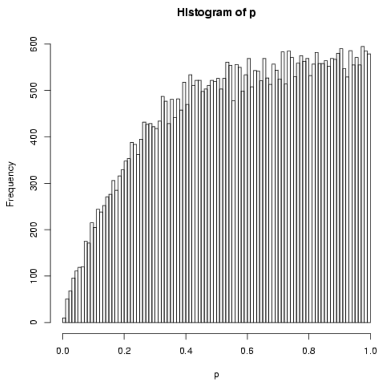  | no | unreasonable p-value distribution |

| | | | | | |
|---|---|---|---|---|---|
| E-GEOD-8289 | hnf4 | 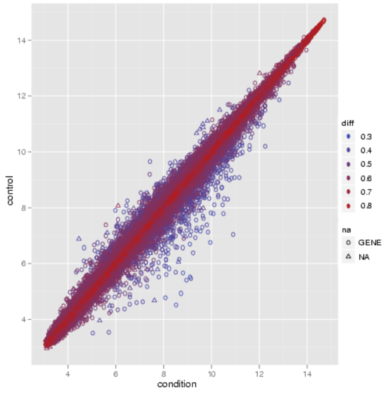  | 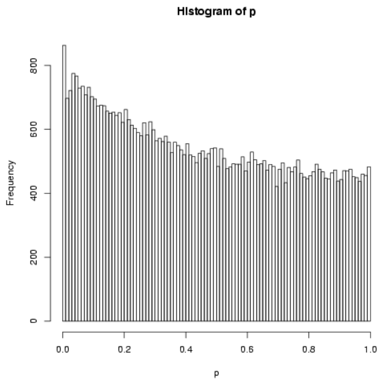  | no | unreasonable p-value distribution |
| E-GEOD-8944 | creb | 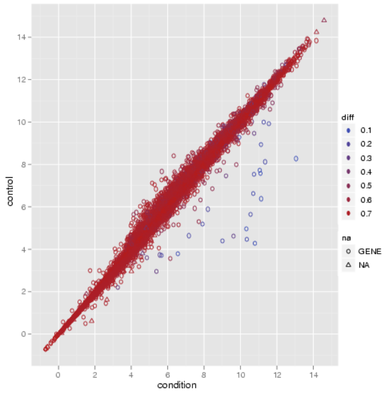  | 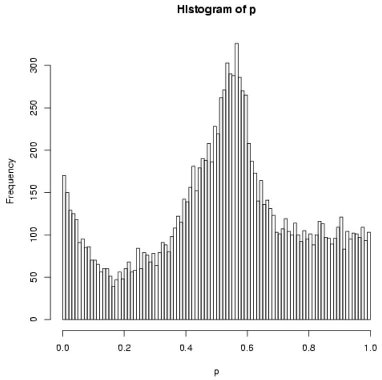  | no | unreasonable p-value distribution |

| | | | | | |
|---|---|---|---|---|---|
| E-GEOD-8946 | creb | 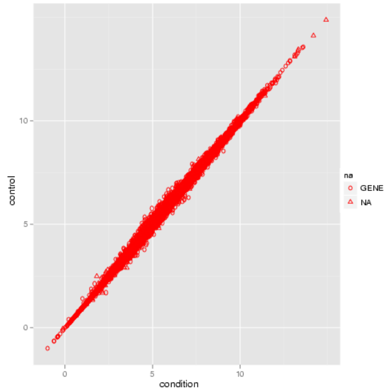 | 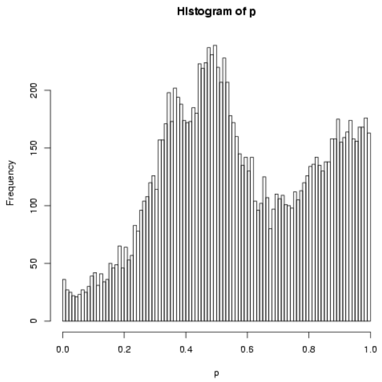 | no | unreasonable p-value distribution |
| E-GEOD-9124 | sp3 | 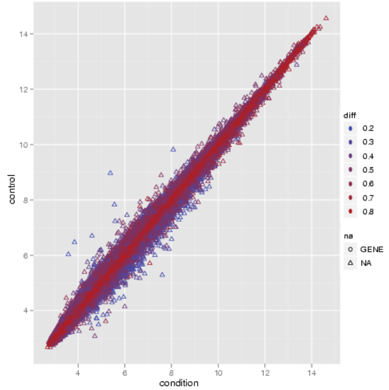 | 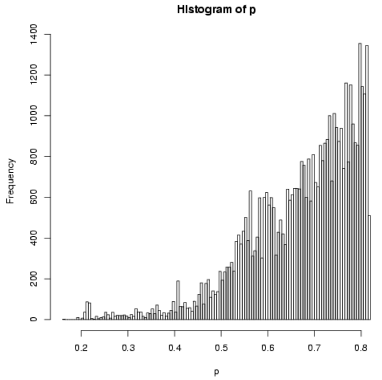 | no | unreasonable p-value distribution |

| | | | | | |
|---|---|---|---|---|---|
| E-GEOD-9188 | pbx | 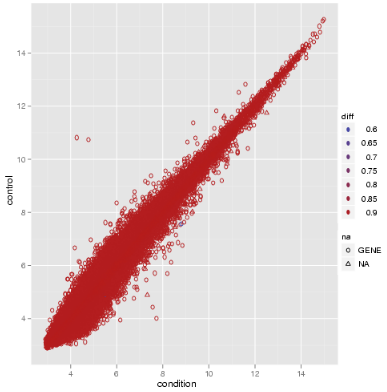 | 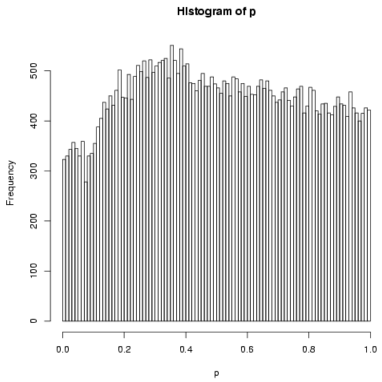 | no | unreasonable p-value distribution |
| E-GEOD-9198 | pbx | 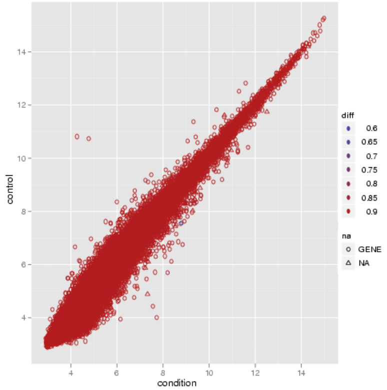 | 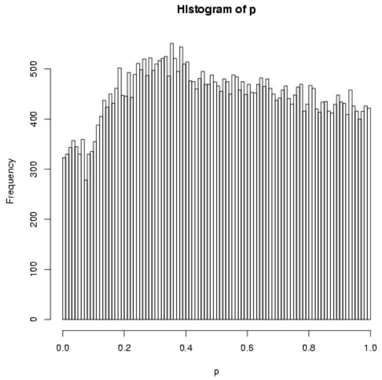 | no | unreasonable p-value distribution |

| | | | | | |
|---|---|---|---|---|---|
| E-MEXP-1040 | foxa | 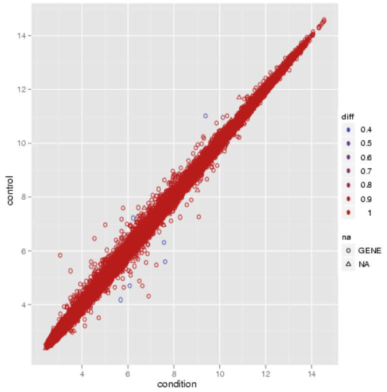 | 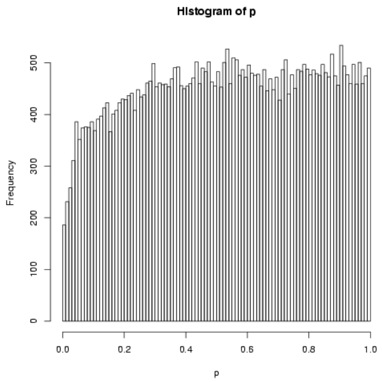 | no | unreasonable p-value distribution |
| E-MEXP-1131 | e2f2 | 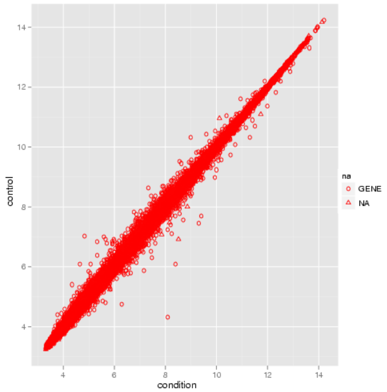 | 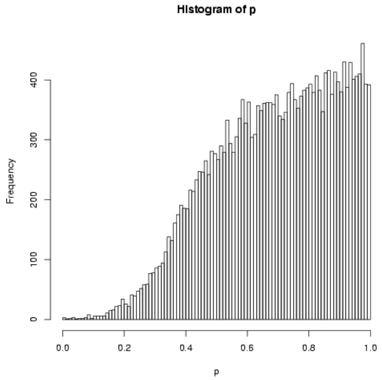 | no | unreasonable p-value distribution |

| | | | | | |
|---|---|---|---|---|---|
| E-MEXP-1511 | MEF2C | 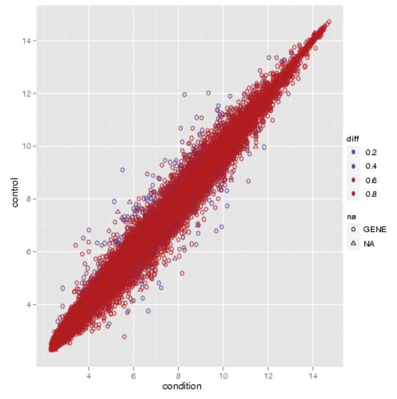 | 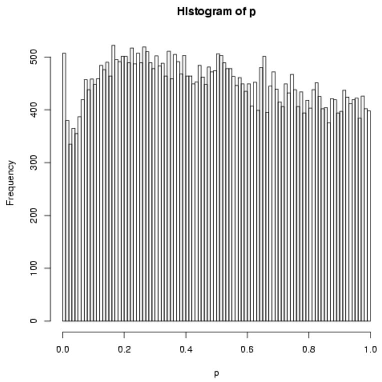 | no | unreasonable p-value distribution |
| E-MEXP-1711 | rarx | 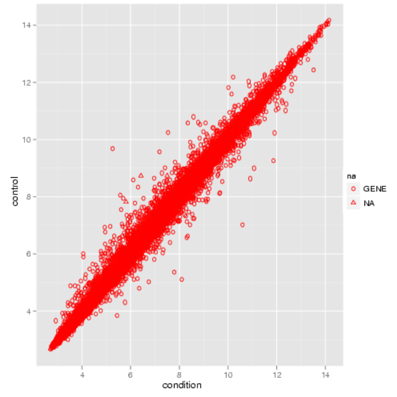 | 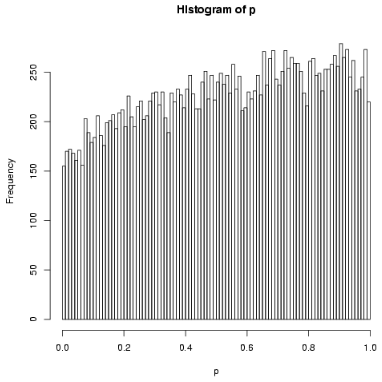 | no | unreasonable p-value distribution |

| | | | | | |
|---|---|---|---|---|---|
| E-MEXP-688 | pbx | 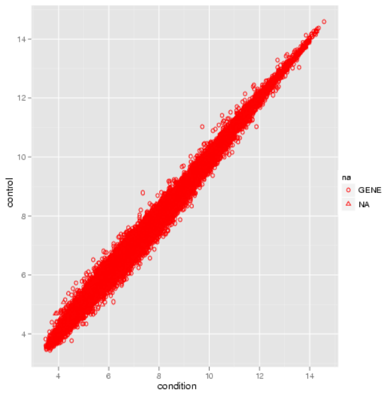 | 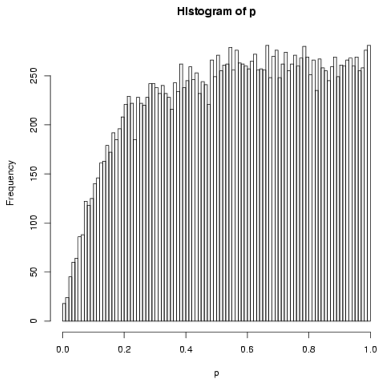 | no | unreasonable p-value distribution |
