# Supplementary figures and images for: A computational evaluation of over-representation of regulatory motifs in the promoter regions of differentially expressed genes
Source: BMC Bioinformatics. 2010 May 20;11:267. doi: 10.1186/1471-2105-11-267 (PMC3098066; doi:10.1186/1471-2105-11-267)

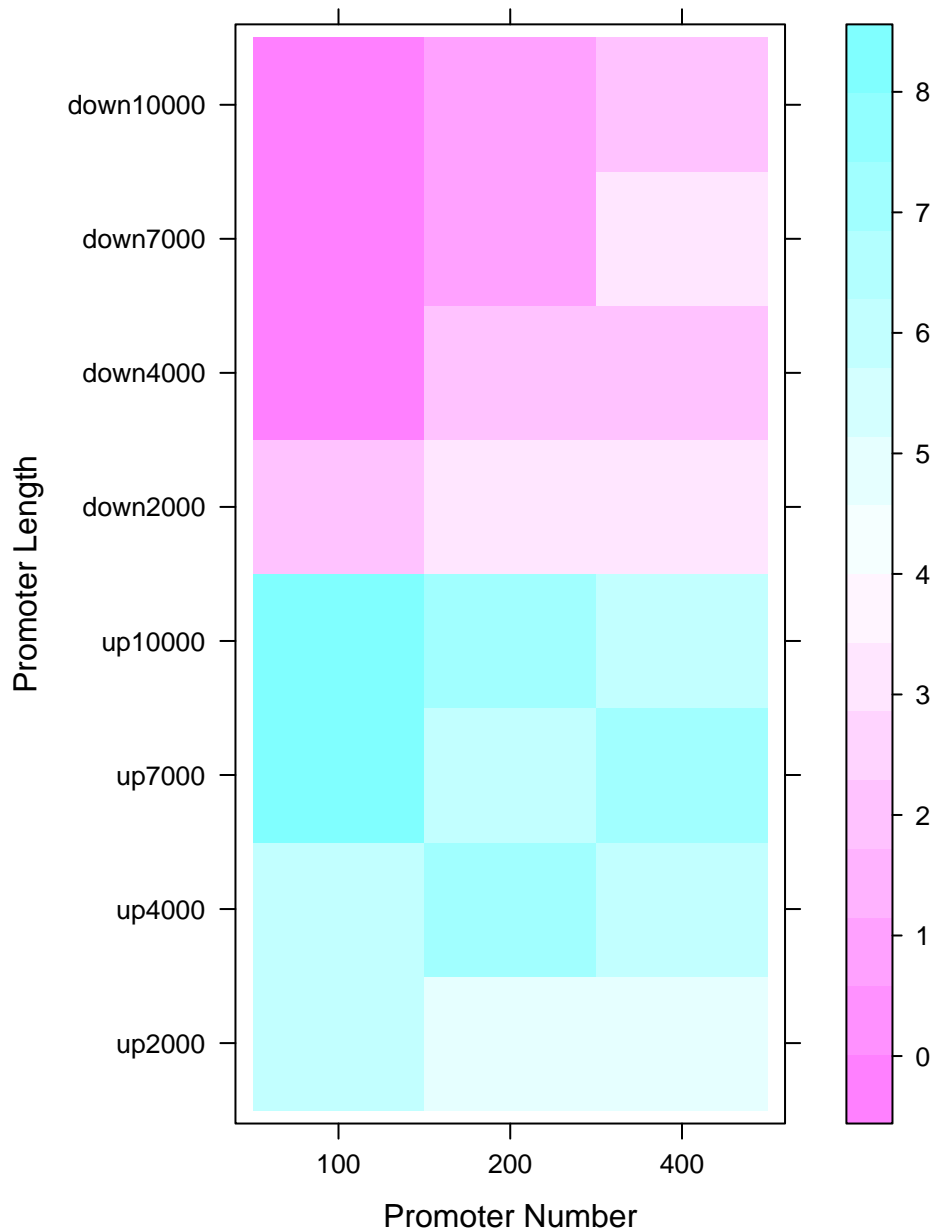

Supplement: Additional file 2 — The parameter preference of oPOSSUM. In this figure, we described number of experiments with significantly over-represented TF binding sites under different promoter number and length. [file 1471-2105-11-267-S2.PDF]
