# Supplementary material for: A computational evaluation of over-representation of regulatory motifs in the promoter regions of differentially expressed genes
Source: BMC Bioinformatics. 2010 May 20;11:267. doi: 10.1186/1471-2105-11-267 (PMC3098066; doi:10.1186/1471-2105-11-267)
Supplement: Additional file 4 — Target matrices mapped to familial binding profiles of Jasparmatrices. Sandelin and Wasserman had classified the Jaspar matrices into the 11 familial binding profiles, which was based on TF structural information as well as binding matrix similarity of Jaspar matrices [41]. We highlighted the 30 target matrices associated with our work to the each of these 11 familial classes. [file 1471-2105-11-267-S4.PDF]

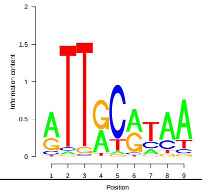	bZIP cEBP-like<br><br>Ddit3-Cebpa<br>NFIL3<br>HLF<br>Cebpa	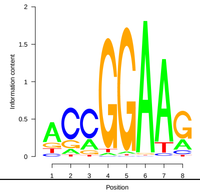	ETS<br><br>Eip74EF ELK1<br>GABPA ELK4<br>SPI1 SPIB<br>ETS1
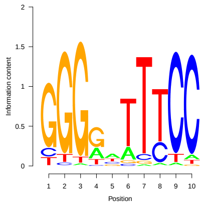	REL<br>dl_1<br>Dl_2<br>NF-kappaB<br>REL<br>NFKB1<br>RELA	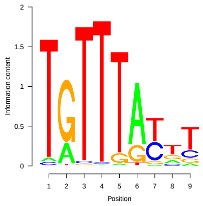	Forkhead<br><br>FOXF2 FOXD1<br>FOXC1 FOXL1<br>Foxq1 Foxd3<br>FOXI1<br>Foxa2
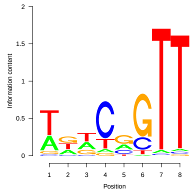	TRP(MYB)<br><br>GAMYB<br>IRF1<br>IRF2<br>MYB.ph3<br>Myb	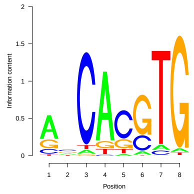	bHLH(zip)<br><br>Arnt Arnt-Ahr<br>NHLH1 Myf<br>MAX MYC-MAX<br>TAL1-TCF3 Hand1-Tcfe2a<br>USF1 Mycn
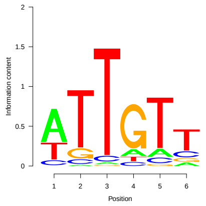	HMG<br>HMG-1<br>HMG-IY<br>SOX9<br>Sox17<br>SRY<br>Sox5	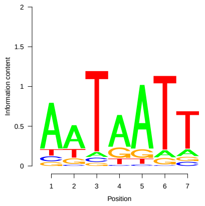	Homeobox<br><br>Athb-1 En1<br>HNF1A Nkx2-5<br>Pax4 PBX1<br>Prrx2<br>Ubx
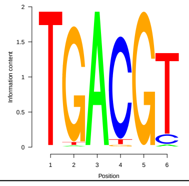	bZIP CREB/G-box-like<br>CREB1<br>TCF11-MafG<br>bZIP910<br>bZIP911	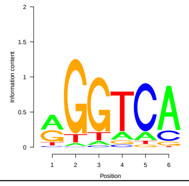	Nuclear Receptor<br>Ar usp<br>NR2F1 PPARG-RXRA<br>PPARG RORA_1<br>RORA_2<br>RXRA-VDR
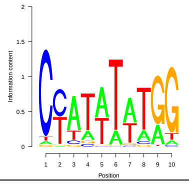	MADS<br>AGL3<br>Agamous<br>MEF2A<br>SQUA<br>SRF	Others Matrices<br><br>TP53, Gata1, GATA2, HNF4A<br>GATA3, Klf4, ESR1, E2F1<br>RREB1, NFYA, Bapx1, Lhx3, Pax6, SRF<br>...	
