# Supplementary material for: A computational evaluation of over-representation of regulatory motifs in the promoter regions of differentially expressed genes
Source: BMC Bioinformatics. 2010 May 20;11:267. doi: 10.1186/1471-2105-11-267 (PMC3098066; doi:10.1186/1471-2105-11-267)
Supplement: Additional file 5 — The output of oPOSSUMfor 33 experiments. The differentially expressed genes from microarray analysis were input into oPOSSUM for over-representation analysis. In this file, we gave outputs of oPOSSUM for each experiment and supplied 33 tables for over-representation status under different promoter number and length. [file 1471-2105-11-267-S5.PDF]

The output of oPOSSUM analysis for 33 experiments

The differentially expressed genes from microarray analysis were input into oPOSSUM for the TF binding sites over-representation analysis. As gene number and promoter length were critical factors for oPOSSUM output, we use promoter length of 4000 bp and promoter number of 200 as default parameter setting. Once under default setting, target matrices were not found to be over-represented, we tried other parameter values.

For the other parameters, they were set as following:

1. Top 30% of conserved regions (min. conservation 60%)
2. Matrix match threshold: 85%

In this supplement, we gave outputs of oPOSSUM for each experiment. The first two were the outputs of 200 up/down-regulated genes with promoter length of -2000~2000 bp (TSS). If either of them could achieve the significantly over-representation, we would not gave further output here; else, we would give the output that targeted matrices were significantly over-represented if possible.

1. The output of oPOSSUM for E-GEOD-10954

TF	TF Class	TF Supergroup	IC	Background gene hits	Background gene non-hits	Target gene hits	Target gene non-hits	Background TFBS hits	Background TFBS rate	Target TFBS hits	Target TFBS rate	Z-score	Fisher score
Nkx2-5	HOMEO	vertebrate	8.270	12616	2534	149	13	96540	0.0241	1771	0.0295	22.66	1.058e-03
Prrx2	HOMEO	vertebrate	9.063	11710	3440	139	23	80966	0.0145	1498	0.0178	18.27	4.820e-03
Lhx3	HOMEO	vertebrate	12.941	7325	7825	109	53	21103	0.0068	416	0.0089	16.78	9.999e-07
Pdx1	HOMEO	vertebrate	9.040	12302	2848	141	21	86351	0.0185	1513	0.0216	14.9	3.239e-02
SRY	HMG	vertebrate	9.193	11001	4149	139	23	53158	0.0171	922	0.0198	13.3	5.133e-05
Nobox	HOMEO	vertebrate	9.573	10484	4666	138	24	42827	0.0122	754	0.0144	12.48	2.341e-06
Sox5	HMG	vertebrate	10.831	11265	3885	144	18	57233	0.0143	995	0.0166	12.36	3.976e-06
NKX3-1	HOMEO	vertebrate	11.127	7537	7613	112	50	19697	0.0049	374	0.0062	12.08	5.129e-07
FOXI1	FORKHEAD	vertebrate	13.183	8038	7112	116	46	22048	0.0095	375	0.0107	8.431	1.207e-06
Cebpa	bZIP	vertebrate	9.187	7775	7375	112	50	18051	0.0077	307	0.0088	7.617	3.505e-06

c-Myc, 200 up-regulated genes, -2000 to 2000 bp, not over-represented

TF	TF Class	TF Supergroup	IC	Background gene hits	Background gene non-hits	Target gene hits	Target gene non-hits	Background TFBS hits	Background TFBS rate	Target TFBS hits	Target TFBS rate	Z-score	Fisher score
MZF1_1-4	ZN-FINGER, C2H2	vertebrate	8.586	10908	4242	116	31	43933	0.0094	677	0.0139	24.87	3.598e-02
ZNF354C	ZN-FINGER, C2H2	vertebrate	8.958	11501	3649	125	22	47022	0.0101	697	0.0143	22.68	4.859e-03
MZF1_5-13	ZN-FINGER, C2H2	vertebrate	9.400	5875	9275	76	71	11280	0.0040	178	0.0061	17.44	1.050e-03
SP1	ZN-FINGER, C2H2	vertebrate	9.719	5559	9591	77	70	10226	0.0037	158	0.0054	15.57	8.192e-05
REL	REL	vertebrate	10.515	3609	11541	46	101	4965	0.0018	80	0.0027	12.27	2.423e-02
ZEB1	ZN-FINGER, C2H2	vertebrate	8.305	11790	3360	119	28	48005	0.0103	609	0.0125	11.65	2.108e-01
Mycn	bHLH-ZIP	vertebrate	10.443	7434	7716	88	59	15024	0.0032	217	0.0044	11.65	5.742e-03
SRF	MADS	vertebrate	17.965	148	15002	5	142	161	0.0001	6	0.0002	11.4	1.611e-02
NFKB1	REL	vertebrate	15.627	819	14331	16	131	967	0.0004	20	0.0008	10.24	6.503e-03
NF-kappaB	REL	vertebrate	13.345	2704	12446	38	109	3518	0.0013	54	0.0018	8.916	1.019e-02

c-Myc, 200 down-regulated genes, -2000 to 2000 bp, significantly over-represented, Mycn

Promoter Length		Gene Number		
		100	200	400
up	2000 bp	N	N	N
	4000 bp	N	N	W
	7000 bp	N	N	N
	10000 bp	N	N	N
down	2000 bp	N	N	N
	4000 bp	W	S	S
	7000 bp	W	W	S
	10000 bp	W	W	S

c−Myc

2. The output of oPOSSUM for E-GEOD-11352

TF	TF Class	TF Supergroup	IC	Background gene hits	Background gene non-hits	Target gene hits	Target gene non-hits	Background TFBS hits	Background TFBS rate	Target TFBS hits	Target TFBS rate	Z-score	Fisher score
ESR1	NUCLEAR RECEPTOR	vertebrate	17.683	349	14801	11	160	365	0.0002	11	0.0007	16.26	2.406e-03
TLX1-NFIC	HOMEO/CAAT	vertebrate	19.665	534	14616	9	162	562	0.0003	10	0.0005	6.855	1.542e-01
MYC-MAX	bHLH-ZIP	vertebrate	14.237	2630	12520	34	137	3253	0.0013	43	0.0017	6.06	2.197e-01
MAX	bHLH-ZIP	vertebrate	12.685	6081	9069	72	99	10424	0.0037	123	0.0044	5.797	3.274e-01
Evi1	ZN-FINGER, C2H2	vertebrate	17.909	843	14307	12	159	949	0.0005	14	0.0007	5.432	2.471e-01
Ar	NUCLEAR RECEPTOR	vertebrate	15.703	376	14774	6	165	395	0.0003	6	0.0005	4.782	2.550e-01
Staf	ZN-FINGER, C2H2	vertebrate	17.541	1282	13868	15	156	1497	0.0011	19	0.0014	4.627	4.823e-01
Arnt-Ahr	bHLH	vertebrate	9.532	11906	3244	139	32	55098	0.0118	587	0.0126	3.758	2.252e-01
ELF5	ETS	vertebrate	8.693	12664	2486	146	25	63365	0.0204	663	0.0213	3.508	3.058e-01
MIZF	ZN-FINGER, C2H2	vertebrate	13.197	1637	13513	20	151	1934	0.0007	24	0.0009	3.312	3.903e-01

ESRapha, 200 up-regulated genes, -2000 to 2000 bp, significantly over-represented, ESR1

TF	TF Class	TF Supergroup	IC	Background gene hits	Background gene non-hits	Target gene hits	Target gene non-hits	Background TFBS hits	Background TFBS rate	Target TFBS hits	Target TFBS rate	Z-score	Fisher score
FOXI1	FORKHEAD	vertebrate	13.183	8038	7112	109	47	22048	0.0095	349	0.0111	10.54	1.510e-05
FOXF2	FORKHEAD	vertebrate	14.824	2629	12521	44	112	3571	0.0018	67	0.0025	10.21	5.680e-04
Sox5	HMG	vertebrate	10.831	11265	3885	132	24	57233	0.0143	875	0.0163	10.04	1.525e-03
PPARG	NUCLEAR RECEPTOR	vertebrate	20.365	40	15110	2	154	40	0.0000	2	0.0001	8.756	6.819e-02
SRY	HMG	vertebrate	9.193	11001	4149	129	27	53158	0.0171	790	0.0189	8.416	2.333e-03
Pdx1	HOMEO	vertebrate	9.040	12302	2848	143	13	86351	0.0185	1278	0.0204	8.381	2.251e-04
Nobox	HOMEO	vertebrate	9.573	10484	4666	123	33	42827	0.0122	645	0.0137	8.114	4.896e-03
Lhx3	HOMEO	vertebrate	16.354	3615	11535	49	107	6013	0.0028	100	0.0035	7.634	1.984e-02
FOXD1	FORKHEAD	vertebrate	11.926	7400	7750	101	55	16369	0.0047	259	0.0055	7.372	4.946e-05
HNF4A	NUCLEAR RECEPTOR	vertebrate	9.617	5180	9970	70	86	7908	0.0037	127	0.0044	7.182	3.827e-03

ESRapha, 200 down-regulated genes, -2000 to 2000 bp, not over-represented

Promoter Length		Gene Number		
		100	200	400
up	2000 bp	S	W	W
	4000 bp	S	S	S
	7000 bp	S	S	N
	10000 bp	S	S	W
down	2000 bp	N	N	N
	4000 bp	N	N	N
	7000 bp	N	N	N
	10000 bp	N	N	N

ESRalpha

3. The output of oPOSSUM for E-GEOD-11809

TF	TF Class	TF Supergroup	IC	Background gene hits	Background gene non-hits	Target gene hits	Target gene non-hits	Background TFBS hits	Background TFBS rate	Target TFBS hits	Target TFBS rate	Z-score	Fisher score
IRF2	TRP-CLUSTER	vertebrate	21.134	74	15076	6	114	74	0.0000	6	0.0006	35	3.885e-05
IRF1	TRP-CLUSTER	vertebrate	16.008	1283	13867	21	99	1449	0.0006	29	0.0021	23.6	1.189e-03
STAT1	Stat	vertebrate	18.431	417	14733	8	112	436	0.0002	10	0.0008	16.86	1.895e-02
RXRA-VDR	NUCLEAR RECEPTOR	vertebrate	20.451	46	15104	2	118	46	0.0000	2	0.0002	12.38	5.465e-02
NF-kappaB	REL	vertebrate	13.345	2704	12446	28	92	3518	0.0013	36	0.0021	10.03	7.793e-02
ELF5	ETS	vertebrate	8.693	9474	5676	82	38	23856	0.0077	184	0.0098	9.874	1.118e-01
RELA	REL	vertebrate	14.757	1972	13178	23	97	2373	0.0008	26	0.0015	9.656	3.674e-02
SPIB	ETS	vertebrate	9.060	10919	4231	94	26	35925	0.0090	257	0.0106	7.076	7.509e-02
REL	REL	vertebrate	10.515	3609	11541	33	87	4965	0.0018	37	0.0022	3.965	2.005e-01
Myf	bHLH	vertebrate	15.914	3002	12148	22	98	4031	0.0017	28	0.0020	2.481	6.927e-01

IRF1, 200 up-regulated genes, -2000 to 2000 bp, significantly over-represented, IRF1, IRF2

TF	TF Class	TF Supergroup	IC	Background gene hits	Background gene non-hits	Target gene hits	Target gene non-hits	Background TFBS hits	Background TFBS rate	Target TFBS hits	Target TFBS rate	Z-score	Fisher score
TEAD1	TEA	vertebrate	15.678	997	14153	29	133	1114	0.0005	37	0.0009	13.08	1.026e-06
SOX9	HMG	vertebrate	9.079	4026	11124	75	87	6306	0.0020	157	0.0028	12.25	6.911e-08
SRY	HMG	vertebrate	9.193	6914	8236	101	61	16164	0.0052	352	0.0063	10.76	1.553e-05
SRF	MADS	vertebrate	17.965	148	15002	6	156	161	0.0001	8	0.0002	10.3	5.938e-03
FOXI1	FORKHEAD	vertebrate	13.183	4019	11131	67	95	6574	0.0028	148	0.0035	9.452	3.338e-05
Foxd3	FORKHEAD	vertebrate	12.945	3468	11682	58	104	5452	0.0023	125	0.0030	9.394	1.491e-04
Nkx2-5	HOMEO	vertebrate	8.270	9631	5519	130	32	36300	0.0091	743	0.0103	9.316	3.376e-06
NKX3-1	HOMEO	vertebrate	11.127	5231	9919	73	89	10063	0.0025	220	0.0031	7.64	3.711e-03
Prrx2	HOMEO	vertebrate	9.063	8566	6584	116	46	28308	0.0051	579	0.0057	6.893	6.020e-05
Sox5	HMG	vertebrate	10.831	5575	9575	83	79	11203	0.0028	237	0.0033	6.585	1.350e-04

IRF1, 200 down-regulated genes, -2000 to 2000 bp, not over-represented

Promoter Length		Gene Number		
		100	200	400
up	2000 bp	S	S	S
	4000 bp	S	S	S
	7000 bp	S	S	S
	10000 bp	S	S	S
down	2000 bp	N	N	W
	4000 bp	N	N	W
	7000 bp	N	W	W
	10000 bp	N	W	W

IRF1

4. The output of oPOSSUM for E-GEOD-2060

TF	TF Class	TF Supergroup	IC	Background gene hits	Background gene non-hits	Target gene hits	Target gene non-hits	Background TFBS hits	Background TFBS rate	Target TFBS hits	Target TFBS rate	Z-score	Fisher score
Hand1-Tcfe2a	bHLH	vertebrate	10.144	5274	9876	55	58	8336	0.0030	99	0.0045	12.76	1.732e-03
MZF1_1-4	ZN-FINGER, C2H2	vertebrate	8.586	10908	4242	90	23	43933	0.0094	437	0.0118	11.61	4.149e-02
MEF2A	MADS	vertebrate	15.709	1740	13410	24	89	2190	0.0008	30	0.0014	9.532	2.195e-03
REST	ZN-FINGER, C2H2	vertebrate	22.958	112	15038	2	111	119	0.0001	3	0.0003	9.099	2.067e-01
NR3C1	NUCLEAR RECEPTOR	vertebrate	14.749	178	14972	4	109	180	0.0001	4	0.0003	9.027	4.628e-02
ZNF354C	ZN-FINGER, C2H2	vertebrate	8.958	11501	3649	92	21	47022	0.0101	441	0.0119	8.615	1.028e-01
REL	REL	vertebrate	10.515	3609	11541	37	76	4965	0.0018	56	0.0025	8.335	2.014e-02
SP1	ZN-FINGER, C2H2	vertebrate	9.719	5559	9591	48	65	10226	0.0037	104	0.0047	8.005	1.210e-01
Roaz	ZN-FINGER, C2H2	vertebrate	17.925	916	14234	13	100	1024	0.0005	14	0.0009	7.931	1.978e-02
CREB1	bZIP	vertebrate	12.605	1777	13373	20	93	2042	0.0009	25	0.0014	7.529	4.027e-02

CREB, 200 up-regulated genes, -2000 to 2000 bp, not over-represented

TF	TF Class	TF Supergroup	IC	Background gene hits	Background gene non-hits	Target gene hits	Target gene non-hits	Background TFBS hits	Background TFBS rate	Target TFBS hits	Target TFBS rate	Z-score	Fisher score
Foxd3	FORKHEAD	vertebrate	12.945	3468	11682	45	87	5452	0.0023	83	0.0039	16.03	2.339e-03
Lhx3	HOMEO	vertebrate	16.354	1024	14126	21	111	1222	0.0006	25	0.0013	14.73	2.376e-04
SRY	HMG	vertebrate	9.193	6914	8236	69	63	16164	0.0052	206	0.0072	14.07	7.572e-02
FOXI1	FORKHEAD	vertebrate	13.183	4019	11131	41	91	6574	0.0028	89	0.0041	12.67	1.416e-01
Foxa2	FORKHEAD	vertebrate	12.433	3625	11525	42	90	5513	0.0024	74	0.0034	11.28	2.463e-02
FOXD1	FORKHEAD	vertebrate	11.926	5408	9742	56	76	9771	0.0028	123	0.0038	9.836	6.613e-02
IRF1	TRP-CLUSTER	vertebrate	16.008	1283	13867	18	114	1449	0.0006	23	0.0011	9.118	3.096e-02
SOX9	HMG	vertebrate	9.079	4026	11124	41	91	6306	0.0020	81	0.0028	9.01	1.444e-01
Foxq1	FORKHEAD	vertebrate	14.070	1761	13389	25	107	2254	0.0009	33	0.0014	8.88	9.844e-03
Lhx3	HOMEO	vertebrate	12.941	4014	11136	44	88	7208	0.0023	88	0.0031	7.946	4.960e-02

CREB, 200 down-regulated genes, -2000 to 2000 bp, not over-represented

TF	TF Class	TF Supergroup	IC	Background gene hits	Background gene non-hits	Target gene hits	Target gene non-hits	Background TFBS hits	Background TFBS rate	Target TFBS hits	Target TFBS rate	Z-score	Fisher score
CREB1	bZIP	vertebrate	12.605	1331	13819	11	44	1496	0.0012	14	0.0031	12.8	7.751e-03
MEF2A	MADS	vertebrate	15.709	1023	14127	7	48	1203	0.0008	10	0.0018	8.54	7.656e-02
MZF1_1-4	ZN-FINGER, C2H2	vertebrate	8.586	8487	6663	36	19	24492	0.0097	111	0.0123	5.957	1.010e-01
Ddit3-Cebpa	bZIP	vertebrate	11.652	469	14681	4	51	494	0.0004	4	0.0009	5.662	9.138e-02
PBX1	HOMEO	vertebrate	14.641	338	14812	3	52	355	0.0003	3	0.0007	5.148	1.256e-01
NHLH1	bHLH	vertebrate	14.132	825	14325	5	50	918	0.0007	6	0.0013	5.056	1.800e-01
Mycn	bHLH-ZIP	vertebrate	10.443	5216	9934	23	32	8565	0.0034	42	0.0046	4.894	1.565e-01
FOXF2	FORKHEAD	vertebrate	14.824	384	14766	3	52	411	0.0004	3	0.0008	4.566	1.643e-01
REL	REL	vertebrate	10.515	2320	12830	12	43	2927	0.0019	15	0.0028	4.3	1.272e-01
TEAD1	TEA	vertebrate	15.678	588	14562	3	52	624	0.0005	4	0.0009	3.956	3.613e-01

CREB, 100 up-regulated genes, -2000 to 0 bp, significantly over-represented, CREB1

Promoter Length		Gene Number 100	200	400
up	2000 bp	S	N	N
	4000 bp	W	N	N
	7000 bp	W	W	S
	10000 bp	W	W	S
down	2000 bp	N	N	N
	4000 bp	N	N	N
	7000 bp	N	N	N
	10000 bp	N	N	N

CREB

5. The output of oPOSSUM for E-GEOD-2192

TF	TF Class	TF Supergroup	IC	Background gene hits	Background gene non-hits	Target gene hits	Target gene non-hits	Background TFBS hits	Background TFBS rate	Target TFBS hits	Target TFBS rate	Z-score	Fisher score
HNF4A	NUCLEAR RECEPTOR	vertebrate	9.617	1637	13513	34	121	1852	0.0009	40	0.0019	18.64	5.162e-05
NFYA	CAAT-BOX	vertebrate	12.925	2552	12598	43	112	3490	0.0020	61	0.0036	18.56	5.049e-04
PPARG-RXRA	NUCLEAR RECEPTOR	vertebrate	23.449	65	15085	3	152	65	0.0000	3	0.0002	13.17	3.164e-02
NR2F1	NUCLEAR RECEPTOR	vertebrate	15.924	696	14454	15	140	731	0.0004	16	0.0008	12.42	5.724e-03
Pax6	PAIRED	vertebrate	13.798	88	15062	2	153	91	0.0000	4	0.0002	12.24	2.314e-01
Evi1	ZN-FINGER, C2H2	vertebrate	17.909	125	15025	4	151	131	0.0001	4	0.0002	8.914	4.216e-02
SP1	ZN-FINGER, C2H2	vertebrate	9.719	5559	9591	58	97	10226	0.0037	118	0.0043	5.858	4.562e-01
HLF	bZIP	vertebrate	11.147	1294	13856	17	138	1495	0.0006	20	0.0009	4.916	1.745e-01
SRF	MADS	vertebrate	17.965	148	15002	3	152	161	0.0001	3	0.0001	3.852	1.973e-01
RORA_1	NUCLEAR RECEPTOR	vertebrate	13.190	2565	12585	30	125	3186	0.0011	37	0.0014	3.38	2.405e-01

PPARgamma2, 200 up-regulated genes, -2000 to 2000 bp, weakly over-represented, PPARG-RXRA

TF	TF Class	TF Supergroup	IC	Background gene hits	Background gene non-hits	Target gene hits	Target gene non-hits	Background TFBS hits	Background TFBS rate	Target TFBS hits	Target TFBS rate	Z-score	Fisher score
SRF	MADS	vertebrate	17.965	148	15002	6	157	161	0.0001	7	0.0002	10.35	6.113e-03
Nobox	HOMEO	vertebrate	9.573	6167	8983	84	79	13638	0.0039	232	0.0045	6.343	3.510e-03
RXRA-VDR	NUCLEAR RECEPTOR	vertebrate	20.451	46	15104	2	161	46	0.0000	2	0.0001	6.085	9.249e-02
MEF2A	MADS	vertebrate	15.709	1740	13410	31	132	2190	0.0008	43	0.0010	6.02	3.535e-03
Lhx3	HOMEO	vertebrate	12.941	4014	11136	63	100	7208	0.0023	126	0.0028	5.868	5.029e-04
Foxd3	FORKHEAD	vertebrate	12.945	3468	11682	49	114	5452	0.0023	95	0.0028	5.776	2.145e-02
Hand1-Tcfe2a	bHLH	vertebrate	10.144	5274	9876	73	90	8336	0.0030	142	0.0035	5.594	5.584e-03
Foxa2	FORKHEAD	vertebrate	12.433	3625	11525	51	112	5513	0.0024	95	0.0028	5.398	2.014e-02
REL	REL	vertebrate	10.515	3609	11541	67	96	4965	0.0018	87	0.0021	5.206	9.306e-07
Cebpa	bZIP	vertebrate	9.187	2882	12268	46	117	3755	0.0016	66	0.0019	5.051	2.971e-03

PPARgamma2, 200 down-regulated genes, -2000 to 2000 bp, not over-represented

Promoter Length		Gene Number		
		100	200	400
up	2000 bp	N	N	N
	4000 bp	N	W	N
	7000 bp	N	W	N
	10000 bp	N	N	N
down	2000 bp	N	N	N
	4000 bp	N	N	N
	7000 bp	N	N	N
	10000 bp	N	N	N

PPARgamma2

6. The output of oPOSSUM for E-GEOD-3126

TF	TF Class	TF Supergroup	IC	Background gene hits	Background gene non-hits	Target gene hits	Target gene non-hits	Background TFBS hits	Background TFBS rate	Target TFBS hits	Target TFBS rate	Z-score	Fisher score
HNF4A	NUCLEAR RECEPTOR	vertebrate	9.617	1637	13513	43	107	1852	0.0009	55	0.0025	29.37	1.903e-09
FOXI1	FORKHEAD	vertebrate	13.183	4019	11131	48	102	6574	0.0028	96	0.0040	11.61	8.013e-02
NR2F1	NUCLEAR RECEPTOR	vertebrate	15.924	696	14454	15	135	731	0.0004	16	0.0008	11.36	4.237e-03
HNF1A	HOMEO	vertebrate	15.548	459	14691	12	138	496	0.0002	12	0.0006	11.23	2.341e-03
RXRA-VDR	NUCLEAR RECEPTOR	vertebrate	20.451	46	15104	2	148	46	0.0000	2	0.0001	8.34	8.040e-02
RORA_1	NUCLEAR RECEPTOR	vertebrate	13.190	2565	12585	34	116	3186	0.0011	48	0.0017	8.165	4.356e-02
Foxd3	FORKHEAD	vertebrate	12.945	3468	11682	43	107	5452	0.0023	74	0.0031	7.976	6.008e-02
TLX1-NFIC	HOMEO/CAAT	vertebrate	19.665	146	15004	4	146	148	0.0001	4	0.0002	7.322	6.017e-02
PPARG-RXRA	NUCLEAR RECEPTOR	vertebrate	23.449	65	15085	2	148	65	0.0000	2	0.0001	7.072	1.401e-01
Foxq1	FORKHEAD	vertebrate	14.070	1761	13389	23	127	2254	0.0009	33	0.0012	6.537	1.031e-01

HNF4a, 200 up-regulated genes, -2000 to 2000 bp, significantly over-represented, HNF4A

TF	TF Class	TF Supergroup	IC	Background gene hits	Background gene non-hits	Target gene hits	Target gene non-hits	Background TFBS hits	Background TFBS rate	Target TFBS hits	Target TFBS rate	Z-score	Fisher score
HNF1A	HOMEO	vertebrate	15.548	459	14691	17	123	496	0.0002	21	0.0013	32.21	1.668e-06
ESR1	NUCLEAR RECEPTOR	vertebrate	17.683	38	15112	3	137	38	0.0000	3	0.0002	20.68	6.211e-03
HLF	bZIP	vertebrate	11.147	1294	13856	16	124	1495	0.0006	23	0.0012	11.23	1.443e-01
SRF	MADS	vertebrate	17.965	148	15002	4	136	161	0.0001	4	0.0002	8.246	5.108e-02
HNF4A	NUCLEAR RECEPTOR	vertebrate	9.617	1637	13513	21	119	1852	0.0009	22	0.0013	6.901	7.767e-02
TLX1-NFIC	HOMEO/CAAT	vertebrate	19.665	146	15004	3	137	148	0.0001	3	0.0002	6.207	1.565e-01
STAT1	Stat	vertebrate	18.431	417	14733	4	136	436	0.0002	5	0.0003	3.059	5.409e-01
Foxa2	FORKHEAD	vertebrate	12.433	3625	11525	34	106	5513	0.0024	48	0.0026	2.299	4.934e-01
Cebpa	bZIP	vertebrate	9.187	2882	12268	28	112	3755	0.0016	33	0.0018	2.083	4.184e-01
NR2F1	NUCLEAR RECEPTOR	vertebrate	15.924	696	14454	7	133	731	0.0004	7	0.0004	1.845	4.654e-01

HNF4a, 200 down-regulated genes, -2000 to 2000 bp, significantly over-represented, HNF1A

Promoter Length		Gene Number 100	200	400
up	2000 bp	S	S	S
	4000 bp	S	S	S
	7000 bp	S	S	S
	10000 bp	S	S	S
down	2000 bp	S	S	S
	4000 bp	N	N	N
	7000 bp	W	N	N
	10000 bp	W	N	N

HNF4a

7. The output of oPOSSUM for E-GEOD-3244

TF	TF Class	TF Supergroup	IC	Background gene hits	Background gene non-hits	Target gene hits	Target gene non-hits	Background TFBS hits	Background TFBS rate	Target TFBS hits	Target TFBS rate	Z-score	Fisher score
Myf	bHLH	vertebrate	15.914	3002	12148	48	122	4031	0.0017	77	0.0028	14.84	5.483e-03
NFKB1	REL	vertebrate	15.627	819	14331	19	151	967	0.0004	25	0.0008	13.34	2.426e-03
Ar	NUCLEAR RECEPTOR	vertebrate	15.703	40	15110	2	168	40	0.0000	2	0.0001	10.3	7.899e-02
TAL1-TCF3	bHLH	vertebrate	14.070	1503	13647	27	143	1783	0.0008	34	0.0012	9.805	1.027e-02
SPIB	ETS	vertebrate	9.060	10919	4231	130	40	35925	0.0090	485	0.0103	8.003	1.167e-01
MZF1_1-4	ZN-FINGER, C2H2	vertebrate	8.586	10908	4242	135	35	43933	0.0094	588	0.0107	7.643	1.771e-02
NHLH1	bHLH	vertebrate	14.132	1272	13878	19	151	1468	0.0006	26	0.0009	7.229	1.251e-01
ZNF354C	ZN-FINGER, C2H2	vertebrate	8.958	11501	3649	144	26	47022	0.0101	620	0.0113	6.932	3.612e-03
SP1	ZN-FINGER, C2H2	vertebrate	9.719	5559	9591	66	104	10226	0.0037	144	0.0044	6.798	3.092e-01
TLX1-NFIC	HOMEO/CAAT	vertebrate	19.665	146	15004	4	166	148	0.0001	4	0.0002	6.297	8.590e-02

p53, 200 up-regulated genes, -2000 to 2000 bp, not over-represented

TF	TF Class	TF Supergroup	IC	Background gene hits	Background gene non-hits	Target gene hits	Target gene non-hits	Background TFBS hits	Background TFBS rate	Target TFBS hits	Target TFBS rate	Z-score	Fisher score
NFYA	CAAT-BOX	vertebrate	12.925	2552	12598	26	99	3490	0.0020	40	0.0037	16.21	1.459e-01
ELK4	ETS	vertebrate	14.123	2095	13055	25	100	2520	0.0008	31	0.0016	11.92	3.626e-02
E2F1	E2F_TDP	vertebrate	13.838	2129	13021	26	99	2672	0.0008	33	0.0015	11.66	2.547e-02
IRF2	TRP-CLUSTER	vertebrate	21.134	74	15076	2	123	74	0.0000	2	0.0002	9.604	1.283e-01
GABPA	ETS	vertebrate	13.890	2256	12894	21	104	2742	0.0010	29	0.0017	9.458	3.110e-01
ELK1	ETS	vertebrate	8.812	6419	8731	52	73	11166	0.0040	87	0.0051	7.235	6.025e-01
Cebpa	bZIP	vertebrate	9.187	2882	12268	25	100	3755	0.0016	30	0.0021	5.111	4.265e-01
TEAD1	TEA	vertebrate	15.678	997	14153	7	118	1114	0.0005	10	0.0007	4.205	7.220e-01
HNF1A	HOMEO	vertebrate	15.548	459	14691	5	120	496	0.0002	5	0.0004	4.172	3.308e-01
Sox17	HMG	vertebrate	10.502	5970	9180	47	78	10974	0.0035	78	0.0041	4.044	6.909e-01

p53, 200 down-regulated genes, -2000 to 2000 bp, not over-represented

TF	TF Class	TF Supergroup	IC	Background gene hits	Background gene non-hits	Target gene hits	Target gene non-hits	Background TFBS hits	Background TFBS rate	Target TFBS hits	Target TFBS rate	Z-score	Fisher score
TP53	P53	vertebrate	26.239	23	15127	2	83	23	0.0000	2	0.0002	22.06	8.491e-03
Ar	NUCLEAR RECEPTOR	vertebrate	15.703	560	14590	8	77	596	0.0003	9	0.0008	13.34	1.383e-02
MZF1_1-4	ZN-FINGER, C2H2	vertebrate	8.586	14244	906	82	3	219015	0.0319	1489	0.0360	11.63	2.457e-01
SP1	ZN-FINGER, C2H2	vertebrate	9.719	10735	4415	72	13	45625	0.0111	331	0.0133	10.76	2.365e-03
Myf	bHLH	vertebrate	15.914	8466	6684	59	26	21452	0.0063	163	0.0079	10.31	7.563e-03
Roaz	ZN-FINGER, C2H2	vertebrate	17.925	5142	10008	37	48	8897	0.0032	73	0.0044	10.27	4.217e-02
ZNF354C	ZN-FINGER, C2H2	vertebrate	8.958	14287	863	84	1	197374	0.0288	1314	0.0318	9.009	4.235e-02
ZEB1	ZN-FINGER, C2H2	vertebrate	8.305	14512	638	84	1	265002	0.0386	1725	0.0417	8.041	1.230e-01
GABPA	ETS	vertebrate	13.890	6393	8757	49	36	11187	0.0027	87	0.0035	7.546	3.017e-03
ELK4	ETS	vertebrate	14.123	5222	9928	41	44	7904	0.0017	65	0.0024	7.542	6.215e-03

p53, 100 up-regulated genes, -5000 to 2000 bp, signifcantly over-represented

Promoter Length		Gene Number		
		100	200	400
up	2000 bp	N	N	N
	4000 bp	N	N	W
	7000 bp	S	W	W
	10000 bp	S	W	W
down	2000 bp	N	N	N
	4000 bp	N	N	N
	7000 bp	N	N	N
	10000 bp	N	N	N

p53

8. The output of oPOSSUM for E-GEOD-6077

TF	TF Class	TF Supergroup	IC	Background gene hits	Background gene non-hits	Target gene hits	Target gene non-hits	Background TFBS hits	Background TFBS rate	Target TFBS hits	Target TFBS rate	Z-score	Fisher score
PPARG-RXRA	NUCLEAR RECEPTOR	vertebrate	23.449	65	15085	3	137	65	0.0000	3	0.0003	17.54	2.444e-02
MYC-MAX	bHLH-ZIP	vertebrate	14.237	828	14322	15	125	886	0.0003	15	0.0009	12.68	1.035e-02
ELK1	ETS	vertebrate	8.812	6419	8731	64	76	11166	0.0040	104	0.0057	11.59	2.379e-01
E2F1	E2F_TDP	vertebrate	13.838	2129	13021	24	116	2672	0.0008	32	0.0014	9.854	1.764e-01
Staf	ZN-FINGER, C2H2	vertebrate	17.541	312	14838	5	135	324	0.0002	5	0.0005	8.817	1.659e-01
NFYA	CAAT-BOX	vertebrate	12.925	2552	12598	25	115	3490	0.0020	33	0.0029	8.592	4.096e-01
Arnt	bHLH	vertebrate	10.992	2673	12477	32	108	3594	0.0008	40	0.0013	8.357	7.038e-02
MAX	bHLH-ZIP	vertebrate	12.685	2474	12676	26	114	3097	0.0011	32	0.0018	8.293	2.692e-01
NR2F1	NUCLEAR RECEPTOR	vertebrate	15.924	696	14454	8	132	731	0.0004	8	0.0006	5.491	3.170e-01
MIZF	ZN-FINGER, C2H2	vertebrate	13.197	278	14872	4	136	296	0.0001	4	0.0002	4.604	2.588e-01

nmyc, 200 up-regulated genes, -2000 to 2000 bp, significantly over-represented, MYC-MAX

TF	TF Class	TF Supergroup	IC	Background gene hits	Background gene non-hits	Target gene hits	Target gene non-hits	Background TFBS hits	Background TFBS rate	Target TFBS hits	Target TFBS rate	Z-score	Fisher score
Pax6	PAIRED	vertebrate	13.798	88	15062	2	148	91	0.0000	4	0.0002	9.665	2.207e-01
SRF	MADS	vertebrate	17.965	148	15002	4	146	161	0.0001	6	0.0002	9.361	6.256e-02
Sox17	HMG	vertebrate	10.502	5970	9180	91	59	10974	0.0035	178	0.0045	9.316	1.365e-07
SRY	HMG	vertebrate	9.193	6914	8236	91	59	16164	0.0052	245	0.0061	7.737	1.650e-04
Evi1	ZN-FINGER, C2H2	vertebrate	17.909	125	15025	4	146	131	0.0001	4	0.0002	6.572	3.815e-02
MEF2A	MADS	vertebrate	15.709	1740	13410	32	118	2190	0.0008	39	0.0011	6.431	4.291e-04
Sox5	HMG	vertebrate	10.831	5575	9575	77	73	11203	0.0028	173	0.0034	6.372	2.118e-04
SOX9	HMG	vertebrate	9.079	4026	11124	61	89	6306	0.0020	100	0.0025	6.287	1.343e-04
SPIB	ETS	vertebrate	9.060	10919	4231	130	20	35925	0.0090	506	0.0098	5.435	1.715e-05
TEAD1	TEA	vertebrate	15.678	997	14153	18	132	1114	0.0005	20	0.0007	5.157	1.050e-02

nmyc, 200 down-regulated genes, -2000 to 2000 bp, not over-represented

Promoter Length		Gene Number		
		100	200	400
up	2000 bp	N	N	N
	4000 bp	S	S	W
	7000 bp	S	W	N
	10000 bp	S	S	W
down	2000 bp	N	N	W
	4000 bp	N	N	W
	7000 bp	N	W	W
	10000 bp	N	W	W

nmyc

9. The output of oPOSSUM for E-GEOD-6487

TF	TF Class	TF Supergroup	IC	Background gene hits	Background gene non-hits	Target gene hits	Target gene non-hits	Background TFBS hits	Background TFBS rate	Target TFBS hits	Target TFBS rate	Z-score	Fisher score
MEF2A	MADS	vertebrate	15.709	1740	13410	41	108	2190	0.0008	59	0.0019	22.08	8.602e-08
Myf	bHLH	vertebrate	15.914	3002	12148	54	95	4031	0.0017	82	0.0032	19.11	2.644e-06
TEAD1	TEA	vertebrate	15.678	997	14153	24	125	1114	0.0005	28	0.0011	15.26	4.909e-05
NHLH1	bHLH	vertebrate	14.132	1272	13878	25	124	1468	0.0006	34	0.0013	15.05	7.305e-04
Myb	TRP-CLUSTER	vertebrate	9.883	5977	9173	79	70	10296	0.0029	166	0.0043	13.48	5.800e-04
SRF	MADS	vertebrate	17.965	148	15002	4	145	161	0.0001	5	0.0002	8.17	6.135e-02
Evi1	ZN-FINGER, C2H2	vertebrate	17.909	125	15025	4	145	131	0.0001	4	0.0002	7.743	3.737e-02
ZNF354C	ZN-FINGER, C2H2	vertebrate	8.958	11501	3649	128	21	47022	0.0101	596	0.0115	7.644	1.970e-03
SP1	ZN-FINGER, C2H2	vertebrate	9.719	5559	9591	57	92	10226	0.0037	131	0.0042	4.989	3.765e-01
TAL1-TCF3	bHLH	vertebrate	14.070	1503	13647	20	129	1783	0.0008	26	0.0010	4.707	1.029e-01

Myod/Myf, 200 up-regulated genes, -2000 to 2000 bp, significantly over-represented, Myf

TF	TF Class	TF Supergroup	IC	Background gene hits	Background gene non-hits	Target gene hits	Target gene non-hits	Background TFBS hits	Background TFBS rate	Target TFBS hits	Target TFBS rate	Z-score	Fisher score
SRY	HMG	vertebrate	9.193	6914	8236	89	67	16164	0.0052	286	0.0068	13.72	2.881e-03
FOXI1	FORKHEAD	vertebrate	13.183	4019	11131	51	105	6574	0.0028	119	0.0038	11.07	5.240e-02
SOX9	HMG	vertebrate	9.079	4026	11124	61	95	6306	0.0020	115	0.0027	9.656	4.658e-04
PBX1	HOMEO	vertebrate	14.641	597	14553	12	144	653	0.0003	17	0.0005	9.48	2.203e-02
RELA	REL	vertebrate	14.757	1972	13178	36	120	2373	0.0008	48	0.0013	8.862	4.396e-04
ZNF354C	ZN-FINGER, C2H2	vertebrate	8.958	11501	3649	134	22	47022	0.0101	720	0.0114	8.22	1.577e-03
TAL1-TCF3	bHLH	vertebrate	14.070	1503	13647	28	128	1783	0.0008	35	0.0011	7.66	1.594e-03
REL	REL	vertebrate	10.515	3609	11541	52	104	4965	0.0018	86	0.0023	7.272	4.738e-03
FOXD1	FORKHEAD	vertebrate	11.926	5408	9742	66	90	9771	0.0028	160	0.0034	6.859	5.273e-02
Sox5	HMG	vertebrate	10.831	5575	9575	78	78	11203	0.0028	183	0.0034	6.774	5.493e-04

Myod/Myf, 200 down-regulated genes, -2000 to 2000 bp, not over-represented

Promoter Length		Gene Number		
		100	200	400
up	2000 bp	S	S	S
	4000 bp	S	S	S
	7000 bp	S	S	S
	10000 bp	S	S	S
down	2000 bp	N	N	N
	4000 bp	N	N	N
	7000 bp	N	N	N
	10000 bp	N	W	N

Myf

10. The output of oPOSSUM for E-GEOD-7219

TF	TF Class	TF Supergroup	IC	Background gene hits	Background gene non-hits	Target gene hits	Target gene non-hits	Background TFBS hits	Background TFBS rate	Target TFBS hits	Target TFBS rate	Z-score	Fisher score
IRF2	TRP-CLUSTER	vertebrate	21.134	74	15076	7	136	74	0.0000	7	0.0004	28.42	1.053e-05
NF-kappaB	REL	vertebrate	13.345	2704	12446	52	91	3518	0.0013	78	0.0025	19.08	1.353e-07
IRF1	TRP-CLUSTER	vertebrate	16.008	1283	13867	29	114	1449	0.0006	37	0.0014	17.56	9.988e-06
REL	REL	vertebrate	10.515	3609	11541	59	84	4965	0.0018	87	0.0027	12.95	3.418e-06
MZF1_1-4	ZN-FINGER, C2H2	vertebrate	8.586	10908	4242	119	24	43933	0.0094	606	0.0115	11.92	1.303e-03
ZNF354C	ZN-FINGER, C2H2	vertebrate	8.958	11501	3649	115	28	47022	0.0101	629	0.0119	10.24	1.224e-01
RELA	REL	vertebrate	14.757	1972	13178	36	107	2373	0.0008	42	0.0013	9.186	7.046e-05
Spz1	bHLH-ZIP	vertebrate	11.907	1013	14137	20	123	1130	0.0004	22	0.0008	8.481	1.504e-03
STAT1	Stat	vertebrate	18.431	417	14733	10	133	436	0.0002	10	0.0004	8.456	6.858e-03
ELF5	ETS	vertebrate	8.693	9474	5676	97	46	23856	0.0077	316	0.0090	8.351	1.112e-01

NF-kappaB2, 200 up-regulated genes, -2000 to 2000 bp, significantly over-represented, NF-kappaB

TF	TF Class	TF Supergroup	IC	Background gene hits	Background gene non-hits	Target gene hits	Target gene non-hits	Background TFBS hits	Background TFBS rate	Target TFBS hits	Target TFBS rate	Z-score	Fisher score
NFYA	CAAT-BOX	vertebrate	12.925	2552	12598	33	101	3490	0.0020	46	0.0029	10.37	1.424e-02
E2F1	E2F_TDP	vertebrate	13.838	2129	13021	30	104	2672	0.0008	39	0.0012	8.57	6.283e-03
ELK4	ETS	vertebrate	14.123	2095	13055	29	105	2520	0.0008	36	0.0013	8.337	9.236e-03
GABPA	ETS	vertebrate	13.890	2256	12894	28	106	2742	0.0010	35	0.0014	6.52	3.873e-02
Myf	bHLH	vertebrate	15.914	3002	12148	36	98	4031	0.0017	47	0.0022	6.119	3.018e-02
Nkx2-5	HOMEO	vertebrate	8.270	9631	5519	85	49	36300	0.0091	368	0.0102	6.002	5.521e-01
FOXF2	FORKHEAD	vertebrate	14.824	701	14449	11	123	777	0.0004	11	0.0006	5.605	4.841e-02
Arnt	bHLH	vertebrate	10.992	2673	12477	34	100	3594	0.0008	45	0.0011	5.397	1.611e-02
ELK1	ETS	vertebrate	8.812	6419	8731	65	69	11166	0.0040	116	0.0046	4.845	9.000e-02
Sox5	HMG	vertebrate	10.831	5575	9575	60	74	11203	0.0028	119	0.0033	4.742	3.584e-02

NF-kappaB2, 200 down-regulated genes, -2000 to 2000 bp, not over-represented

Promoter Length		Gene Number 100	200	400
up	2000 bp	S	S	S
	4000 bp	S	S	S
	7000 bp	S	S	S
	10000 bp	S	S	S
down	2000 bp	N	N	N
	4000 bp	N	N	N
	7000 bp	N	N	N
	10000 bp	N	N	N

NF-kappaB2

11. The output of oPOSSUM for E-GEOD-7223

TF	TF Class	TF Supergroup	IC	Background gene hits	Background gene non-hits	Target gene hits	Target gene non-hits	Background TFBS hits	Background TFBS rate	Target TFBS hits	Target TFBS rate	Z-score	Fisher score
CREB1	bZIP	vertebrate	12.605	1777	13373	36	139	2042	0.0009	48	0.0015	12.49	6.087e-04
FOXF2	FORKHEAD	vertebrate	14.824	701	14449	14	161	777	0.0004	19	0.0007	9.132	3.474e-02
Ar	NUCLEAR RECEPTOR	vertebrate	15.703	40	15110	2	173	40	0.0000	2	0.0001	8.863	8.297e-02
HNF4A	NUCLEAR RECEPTOR	vertebrate	9.617	1637	13513	33	142	1852	0.0009	37	0.0012	7.759	1.163e-03
Ddit3-Cebpa	bZIP	vertebrate	11.652	818	14332	17	158	892	0.0004	19	0.0006	6.304	1.496e-02
Foxd3	FORKHEAD	vertebrate	12.945	3468	11682	49	126	5452	0.0023	92	0.0028	6.119	6.814e-02
STAT1	Stat	vertebrate	18.431	417	14733	10	165	436	0.0002	10	0.0004	5.816	2.493e-02
FOXI1	FORKHEAD	vertebrate	13.183	4019	11131	62	113	6574	0.0028	106	0.0032	4.942	6.188e-03
Fos	bZIP	vertebrate	10.670	5773	9377	80	95	9489	0.0027	152	0.0031	4.602	2.459e-02
GABPA	ETS	vertebrate	13.890	2256	12894	41	134	2742	0.0010	47	0.0012	4.316	1.992e-03

AIbZIP, 200 up-regulated genes, -2000 to 2000 bp, significantly over-represented, CREB1

TF	TF Class	TF Supergroup	IC	Background gene hits	Background gene non-hits	Target gene hits	Target gene non-hits	Background TFBS hits	Background TFBS rate	Target TFBS hits	Target TFBS rate	Z-score	Fisher score
NFYA	CAAT-BOX	vertebrate	12.925	2552	12598	64	99	3490	0.0020	85	0.0055	39.08	1.228e-11
E2F1	E2F_TDP	vertebrate	13.838	2129	13021	41	122	2672	0.0008	51	0.0017	15.94	1.348e-04
Staf	ZN-FINGER, C2H2	vertebrate	17.541	312	14838	7	156	324	0.0002	8	0.0006	13.53	5.460e-02
ELK1	ETS	vertebrate	8.812	6419	8731	63	100	11166	0.0040	117	0.0047	5.872	8.499e-01
Gfi	ZN-FINGER, C2H2	vertebrate	9.470	6037	9113	70	93	10976	0.0039	115	0.0047	5.818	2.342e-01
Nkx2-5	HOMEO	vertebrate	8.270	9631	5519	100	63	36300	0.0091	359	0.0102	5.722	7.491e-01
NKX3-1	HOMEO	vertebrate	11.127	5231	9919	53	110	10063	0.0025	109	0.0031	5.653	7.308e-01
FOXI1	FORKHEAD	vertebrate	13.183	4019	11131	45	118	6574	0.0028	70	0.0034	5.435	4.074e-01
Arnt	bHLH	vertebrate	10.992	2673	12477	30	133	3594	0.0008	44	0.0011	5.306	4.319e-01
CREB1	bZIP	vertebrate	12.605	1777	13373	21	142	2042	0.0009	24	0.0012	4.845	3.595e-01

AIbZIP, 200 down-regulated genes, -2000 to 2000 bp, not over-represented

Promoter Length		Gene Number		
		100	200	400
up	2000 bp	N	S	S
	4000 bp	N	S	S
	7000 bp	N	S	S
	10000 bp	N	S	S
down	2000 bp	N	N	N
	4000 bp	N	N	N
	7000 bp	N	N	N
	10000 bp	N	N	N

ABIZIP

12. The output of oPOSSUM for E-GEOD-7835

TF	TF Class	TF Supergroup	IC	Background gene hits	Background gene non-hits	Target gene hits	Target gene non-hits	Background TFBS hits	Background TFBS rate	Target TFBS hits	Target TFBS rate	Z-score	Fisher score
NFYA	CAAT-BOX	vertebrate	12.925	2552	12598	45	93	3490	0.0020	70	0.0050	31.74	5.172e-06
Staf	ZN-FINGER, C2H2	vertebrate	17.541	312	14838	9	129	324	0.0002	10	0.0009	20.45	2.547e-03
ELK1	ETS	vertebrate	8.812	6419	8731	70	68	11166	0.0040	132	0.0059	14.16	2.990e-02
Gfi	ZN-FINGER, C2H2	vertebrate	9.470	6037	9113	71	67	10976	0.0039	125	0.0056	12.43	3.937e-03
GABPA	ETS	vertebrate	13.890	2256	12894	32	106	2742	0.0010	35	0.0016	8.725	6.694e-03
ELK4	ETS	vertebrate	14.123	2095	13055	29	109	2520	0.0008	32	0.0013	7.817	1.377e-02
Prrx2	HOMEO	vertebrate	9.063	8566	6584	85	53	28308	0.0051	275	0.0061	7.087	1.342e-01
CREB1	bZIP	vertebrate	12.605	1777	13373	21	117	2042	0.0009	24	0.0013	6.476	1.299e-01
E2F1	E2F_TDP	vertebrate	13.838	2129	13021	26	112	2672	0.0008	32	0.0011	6.407	7.256e-02
MYC-MAX	bHLH-ZIP	vertebrate	14.237	828	14322	12	126	886	0.0003	12	0.0006	6.023	7.737e-02

HiF1, 200 up-regulated genes, -2000 to 2000 bp, not over-represented

TF	TF Class	TF Supergroup	IC	Background gene hits	Background gene non-hits	Target gene hits	Target gene non-hits	Background TFBS hits	Background TFBS rate	Target TFBS hits	Target TFBS rate	Z-score	Fisher score
Ar	NUCLEAR RECEPTOR	vertebrate	15.703	40	15110	4	154	40	0.0000	4	0.0002	22.93	1.076e-03
SRF	MADS	vertebrate	17.965	148	15002	7	151	161	0.0001	10	0.0003	19.27	1.135e-03
USF1	bHLH-ZIP	vertebrate	11.290	3565	11585	66	92	4939	0.0012	117	0.0023	18.32	3.534e-07
SP1	ZN-FINGER, C2H2	vertebrate	9.719	5559	9591	85	73	10226	0.0037	191	0.0054	17.29	9.898e-06
Arnt	bHLH	vertebrate	10.992	2673	12477	53	105	3594	0.0008	91	0.0015	16.59	1.300e-06
MZF1_1-4	ZN-FINGER, C2H2	vertebrate	8.586	10908	4242	133	25	43933	0.0094	701	0.0119	15.35	2.545e-04
MAX	bHLH-ZIP	vertebrate	12.685	2474	12676	42	116	3097	0.0011	67	0.0019	14.12	7.837e-04
REL	REL	vertebrate	10.515	3609	11541	62	96	4965	0.0018	94	0.0027	12.53	1.331e-05
HLF	bZIP	vertebrate	11.147	1294	13856	25	133	1495	0.0006	33	0.0011	11.25	2.150e-03
RELA	REL	vertebrate	14.757	1972	13178	37	121	2373	0.0008	49	0.0014	11	2.736e-04

HiF1, 200 down-regulated genes, -2000 to 2000 bp, significantly over-represented, Arnt

Promoter Length		Gene Number 100	200	400
up	2000 bp	N	N	N
	4000 bp	N	N	N
	7000 bp	N	N	N
	10000 bp	N	N	N
down	2000 bp	N	S	S
	4000 bp	W	S	S
	7000 bp	W	S	S
	10000 bp	W	S	S

HiF1

13. The output of oPOSSUM for E-GEOD-9786

TF	TF Class	TF Supergroup	IC	Background gene hits	Background gene non-hits	Target gene hits	Target gene non-hits	Background TFBS hits	Background TFBS rate	Target TFBS hits	Target TFBS rate	Z-score	Fisher score
NR2F1	NUCLEAR RECEPTOR	vertebrate	15.924	696	14454	13	141	731	0.0004	16	0.0009	13.22	2.679e-02
HNF4A	NUCLEAR RECEPTOR	vertebrate	9.617	1637	13513	28	126	1852	0.0009	32	0.0016	12.89	4.408e-03
TLX1-NFIC	HOMEO/CAAT	vertebrate	19.665	146	15004	4	150	148	0.0001	4	0.0002	8.286	6.494e-02
SRF	MADS	vertebrate	17.965	148	15002	3	151	161	0.0001	3	0.0001	4.16	1.948e-01
Hand1-Tcfe2a	bHLH	vertebrate	10.144	5274	9876	50	104	8336	0.0030	88	0.0034	3.862	7.543e-01
NHLH1	bHLH	vertebrate	14.132	1272	13878	15	139	1468	0.0006	17	0.0008	3.155	3.144e-01
Cebpa	bZIP	vertebrate	9.187	2882	12268	35	119	3755	0.0016	39	0.0018	2.45	1.447e-01
MIZF	ZN-FINGER, C2H2	vertebrate	13.197	278	14872	4	150	296	0.0001	4	0.0002	2.306	3.159e-01
RORA_1	NUCLEAR RECEPTOR	vertebrate	13.190	2565	12585	31	123	3186	0.0011	33	0.0013	1.999	1.716e-01
Myb	TRP-CLUSTER	vertebrate	9.883	5977	9173	62	92	10296	0.0029	102	0.0031	1.898	4.496e-01

PPAR-alpha, 200 up-regulated genes, -2000 to 2000 bp, not over-represented

TF	TF Class	TF Supergroup	IC	Background gene hits	Background gene non-hits	Target gene hits	Target gene non-hits	Background TFBS hits	Background TFBS rate	Target TFBS hits	Target TFBS rate	Z-score	Fisher score
HNF1A	HOMEO	vertebrate	15.548	459	14691	12	119	496	0.0002	13	0.0008	17.08	7.346e-04
Gfi	ZN-FINGER, C2H2	vertebrate	9.470	6037	9113	62	69	10976	0.0039	115	0.0052	9.567	5.020e-02
PPARG-RXRA	NUCLEAR RECEPTOR	vertebrate	23.449	65	15085	2	129	65	0.0000	2	0.0002	9.112	1.126e-01
HNF4A	NUCLEAR RECEPTOR	vertebrate	9.617	1637	13513	21	110	1852	0.0009	23	0.0014	7.834	4.355e-02
Foxd3	FORKHEAD	vertebrate	12.945	3468	11682	35	96	5452	0.0023	54	0.0029	5.722	1.747e-01
SPIB	ETS	vertebrate	9.060	10919	4231	97	34	35925	0.0090	320	0.0101	5.64	3.478e-01
RORA_2	NUCLEAR RECEPTOR	vertebrate	17.425	584	14566	8	123	624	0.0003	8	0.0005	5.102	1.363e-01
REL	REL	vertebrate	10.515	3609	11541	33	98	4965	0.0018	47	0.0021	3.878	3.899e-01
Hand1-Tcfe2a	bHLH	vertebrate	10.144	5274	9876	47	84	8336	0.0030	75	0.0034	3.516	4.318e-01
STAT1	Stat	vertebrate	18.431	417	14733	3	128	436	0.0002	5	0.0003	3.054	7.025e-01

PPAR-alpha, 200 down-regulated genes, -2000 to 2000 bp, not over-represented

Promoter Length		Gene Number		
		100	200	400
up	2000 bp	N	N	W
	4000 bp	N	N	N
	7000 bp	N	N	N
	10000 bp	N	N	N
down	2000 bp	S	W	W
	4000 bp	W	N	N
	7000 bp	W	N	S
	10000 bp	W	N	W

PPAR-alpha

14. The output of oPOSSUM for E-MEXP-1444

TF	TF Class	TF Supergroup	IC	Background gene hits	Background gene non-hits	Target gene hits	Target gene non-hits	Background TFBS hits	Background TFBS rate	Target TFBS hits	Target TFBS rate	Z-score	Fisher score
SOX9	HMG	vertebrate	9.079	8524	6626	101	44	22208	0.0071	393	0.0097	18.5	6.822e-04
IRF1	TRP-CLUSTER	vertebrate	16.008	4150	11000	60	85	6213	0.0027	121	0.0040	15.53	2.090e-04
Nkx2-5	HOMEO	vertebrate	8.270	12616	2534	126	19	96540	0.0241	1444	0.0278	14.32	1.454e-01
SRY	HMG	vertebrate	9.193	11001	4149	116	29	53158	0.0171	812	0.0201	13.95	2.647e-02
STAT1	Stat	vertebrate	18.431	1802	13348	35	110	2137	0.0011	46	0.0018	12.93	3.535e-05
Sox5	HMG	vertebrate	10.831	11265	3885	116	29	57233	0.0143	876	0.0169	12.92	7.024e-02
Cebpa	bZIP	vertebrate	9.187	7775	7375	97	48	18051	0.0077	286	0.0094	11.67	1.129e-04
Prrx2	HOMEO	vertebrate	9.063	11710	3440	124	21	80966	0.0145	1213	0.0167	11.16	9.498e-03
HLF	bZIP	vertebrate	11.147	4612	10538	66	79	7195	0.0031	123	0.0041	10.57	1.025e-04
Foxa2	FORKHEAD	vertebrate	12.433	7940	7210	88	57	21593	0.0093	328	0.0108	9.84	2.806e-02

Cepba, 200 up-regulated genes, -2000 to 2000 bp, significantly over-represented, Cebpa

TF	TF Class	TF Supergroup	IC	Background gene hits	Background gene non-hits	Target gene hits	Target gene non-hits	Background TFBS hits	Background TFBS rate	Target TFBS hits	Target TFBS rate	Z-score	Fisher score
Sox5	HMG	vertebrate	10.831	11265	3885	104	24	57233	0.0143	730	0.0166	10.61	4.335e-02
FOXF2	FORKHEAD	vertebrate	14.824	2629	12521	40	88	3571	0.0018	55	0.0025	9.352	9.607e-05
Ddit3-Cebpa	bZIP	vertebrate	11.652	3628	11522	46	82	5245	0.0022	78	0.0030	9.231	1.641e-03
IRF2	TRP-CLUSTER	vertebrate	21.134	552	14598	11	117	588	0.0004	12	0.0007	9.174	7.703e-03
Nkx2-5	HOMEO	vertebrate	8.270	12616	2534	123	5	96540	0.0241	1168	0.0265	8.649	7.632e-06
FOXI1	FORKHEAD	vertebrate	13.183	8038	7112	79	49	22048	0.0095	280	0.0109	8.326	3.043e-02
Lhx3	HOMEO	vertebrate	12.941	7325	7825	78	50	21103	0.0068	274	0.0080	8.227	2.938e-03
Ar	NUCLEAR RECEPTOR	vertebrate	15.703	376	14774	8	120	395	0.0003	8	0.0006	8.165	1.555e-02
Nobox	HOMEO	vertebrate	9.573	10484	4666	95	33	42827	0.0122	531	0.0138	7.802	1.287e-01
NR2F1	NUCLEAR RECEPTOR	vertebrate	15.924	2872	12278	36	92	3587	0.0018	52	0.0024	7.439	7.795e-03

Cepba, 200 down-regulated genes, -2000 to 2000 bp, weakly over-represented, Ddit3-Cebpa

Promoter Length		Gene Number 100	200	400
up	2000 bp	N	S	S
	4000 bp	N	S	S
	7000 bp	S	S	W
	10000 bp	W	W	W
down	2000 bp	N	N	N
	4000 bp	N	N	N
	7000 bp	N	N	W
	10000 bp	W	N	W

Cepba

15. The output of oPOSSUM for E-GEOD-590

TF	TF Class	TF Supergroup	IC	Background gene hits	Background gene non-hits	Target gene hits	Target gene non-hits	Background TFBS hits	Background TFBS rate	Target TFBS hits	Target TFBS rate	Z-score	Fisher score
MZF1_1-4	ZN-FINGER, C2H2	vertebrate	8.586	13837	1313	143	7	154947	0.0332	1896	0.0361	9.035	4.752e-02
ZNF354C	ZN-FINGER, C2H2	vertebrate	8.958	13853	1297	144	6	136176	0.0292	1674	0.0319	8.94	2.417e-02
ZEB1	ZN-FINGER, C2H2	vertebrate	8.305	14221	929	144	6	180543	0.0387	2184	0.0416	8.384	1.821e-01
AGL3	MADS	plant	10.588	7584	7566	89	61	17225	0.0062	228	0.0072	7.75	1.451e-02
sna	ZN-FINGER, C2H2	insect	10.706	13063	2087	141	9	85612	0.0184	1055	0.0201	7.252	2.059e-03
TBP	TATA-box		10.198	7609	7541	85	65	18341	0.0098	231	0.0110	6.63	6.819e-02
Agamous	MADS	plant	9.094	9140	6010	102	48	23245	0.0091	291	0.0102	6.025	3.261e-02
NHLH1	bHLH	vertebrate	14.132	4061	11089	56	94	5936	0.0025	81	0.0031	5.995	3.239e-03
SRF	MADS	vertebrate	17.965	535	14615	11	139	583	0.0003	11	0.0004	5.943	1.869e-02
Roaz	ZN-FINGER, C2H2	vertebrate	17.925	4153	10997	59	91	6324	0.0034	84	0.0040	5.871	1.092e-03

USF1, 200 up-regulated genes, -2000 to 2000 bp, not over-represented

TF	TF Class	TF Supergroup	IC	Background gene hits	Background gene non-hits	Target gene hits	Target gene non-hits	Background TFBS hits	Background TFBS rate	Target TFBS hits	Target TFBS rate	Z-score	Fisher score
NR1H2-RXRA	NUCLEAR RECEPTOR	vertebrate	27.878	71	15079	3	145	72	0.0000	3	0.0002	10.23	3.494e-02
STAT1	Stat	vertebrate	18.431	1802	13348	29	119	2137	0.0011	34	0.0016	8.422	4.900e-03
NR2F1	NUCLEAR RECEPTOR	vertebrate	15.924	2872	12278	40	108	3587	0.0018	52	0.0024	7.884	1.070e-02
IRF1	TRP-CLUSTER	vertebrate	16.008	4150	11000	50	98	6213	0.0027	84	0.0033	7.053	5.250e-02
MIZF	ZN-FINGER, C2H2	vertebrate	13.197	1637	13513	21	127	1934	0.0007	30	0.0010	6.226	1.201e-01
Dof3	ZN-FINGER, DOF	plant	9.002	14050	1100	138	10	172067	0.0369	1961	0.0388	5.643	4.876e-01
HNF4A	NUCLEAR RECEPTOR	vertebrate	9.617	5180	9970	58	90	7908	0.0037	100	0.0043	5.592	1.180e-01
Pax6	PAIRED	vertebrate	13.798	693	14457	10	138	753	0.0004	12	0.0006	4.993	1.439e-01
Pax5	PAIRED	vertebrate	12.432	1352	13798	15	133	1534	0.0011	21	0.0014	4.79	3.436e-01
ESR1	NUCLEAR RECEPTOR	vertebrate	17.683	349	14801	6	142	365	0.0002	6	0.0004	4.31	1.305e-01

USF1, 200 down-regulated genes, -2000 to 2000 bp, not over-represented

Promoter Length		Gene Number 100	200	400
up	7000bp	N	N	W
	4000bp	N	N	N
	2000bp	N	N	N
down	7000bp	N	N	N
	4000bp	N	N	N
	2000bp	N	N	N

USF1

16. The output of oPOSSUM for E-GEOD-11039

TF	TF Class	TF Supergroup	IC	Background gene hits	Background gene non-hits	Target gene hits	Target gene non-hits	Background TFBS hits	Background TFBS rate	Target TFBS hits	Target TFBS rate	Z-score	Fisher score
SRF	MADS	vertebrate	17.965	148	15002	7	147	161	0.0001	8	0.0003	15.31	9.785e-04
NF-kappaB	REL	vertebrate	13.345	2704	12446	38	116	3518	0.0013	62	0.0019	10.21	2.120e-02
NHLH1	bHLH	vertebrate	14.132	1272	13878	24	130	1468	0.0006	29	0.0011	9.82	2.552e-03
REL	REL	vertebrate	10.515	3609	11541	46	108	4965	0.0018	81	0.0025	9.446	5.135e-02
RELA	REL	vertebrate	14.757	1972	13178	28	126	2373	0.0008	41	0.0013	7.883	4.269e-02
TAL1-TCF3	bHLH	vertebrate	14.070	1503	13647	28	126	1783	0.0008	31	0.0011	7.629	1.306e-03
RXRA-VDR	NUCLEAR RECEPTOR	vertebrate	20.451	46	15104	2	152	46	0.0000	2	0.0001	7.531	8.406e-02
NFKB1	REL	vertebrate	15.627	819	14331	15	139	967	0.0004	19	0.0006	7.511	2.096e-02
HNF4A	NUCLEAR RECEPTOR	vertebrate	9.617	1637	13513	28	126	1852	0.0009	31	0.0012	7.168	4.408e-03
SPIB	ETS	vertebrate	9.060	10919	4231	120	34	35925	0.0090	471	0.0101	6.466	6.175e-02

e2f2, 200 up-regulated genes, -2000 to 2000 bp, weakly over-represented

TF	TF Class	TF Supergroup	IC	Background gene hits	Background gene non-hits	Target gene hits	Target gene non-hits	Background TFBS hits	Background TFBS rate	Target TFBS hits	Target TFBS rate	Z-score	Fisher score
NFYA	CAAT-BOX	vertebrate	12.925	2552	12598	43	102	3490	0.0020	67	0.0040	23.06	1.029e-04
NR1H2-RXRA	NUCLEAR RECEPTOR	vertebrate	27.878	15	15135	2	143	15	0.0000	2	0.0001	19.82	1.106e-02
Cebpa	bZIP	vertebrate	9.187	2882	12268	34	111	3755	0.0016	53	0.0024	9.695	1.086e-01
Pax6	PAIRED	vertebrate	13.798	88	15062	3	142	91	0.0000	3	0.0002	8.349	5.547e-02
Arnt	bHLH	vertebrate	10.992	2673	12477	38	107	3594	0.0008	54	0.0012	8.049	6.720e-03
PPARG-RXRA	NUCLEAR RECEPTOR	vertebrate	23.449	65	15085	2	143	65	0.0000	2	0.0001	7.629	1.327e-01
Ddit3-Cebpa	bZIP	vertebrate	11.652	818	14332	14	131	892	0.0004	15	0.0007	7.534	2.651e-02
Gfi	ZN-FINGER, C2H2	vertebrate	9.470	6037	9113	76	69	10976	0.0039	129	0.0048	7.181	1.543e-03
USF1	bHLH-ZIP	vertebrate	11.290	3565	11585	46	99	4939	0.0012	66	0.0017	7.059	1.547e-02
Mycn	bHLH-ZIP	vertebrate	10.443	7434	7716	78	67	15024	0.0032	178	0.0040	6.793	1.471e-01

e2f2, 200 down-regulated genes, -2000 to 2000 bp, not over-represented

TF	TF Class	TF Supergroup	IC	Background gene hits	Background gene non-hits	Target gene hits	Target gene non-hits	Background TFBS hits	Background TFBS rate	Target TFBS hits	Target TFBS rate	Z-score	Fisher score
NF-kappaB	REL	vertebrate	13.345	5874	9276	44	36	10146	0.0036	88	0.0057	13.41	2.424e-03
RELA	REL	vertebrate	14.757	4606	10544	31	49	6841	0.0024	60	0.0039	11.34	6.942e-02
HNF4A	NUCLEAR RECEPTOR	vertebrate	9.617	5180	9970	41	39	7908	0.0037	63	0.0053	10.45	1.283e-03
CREB1	bZIP	vertebrate	12.605	4951	10199	39	41	7418	0.0032	60	0.0046	10.21	2.117e-03
NFKB1	REL	vertebrate	15.627	2687	12463	22	58	3691	0.0015	34	0.0024	9.914	2.048e-02
NHLH1	bHLH	vertebrate	14.132	4061	11089	34	46	5936	0.0025	49	0.0038	9.723	1.788e-03
NR3C1	NUCLEAR RECEPTOR	vertebrate	14.749	1835	13315	17	63	2162	0.0014	19	0.0022	8.582	1.459e-02
ELF5	ETS	vertebrate	8.693	12664	2486	74	6	63365	0.0204	403	0.0234	8.399	1.640e-02
E2F1	E2F_TDP	vertebrate	13.838	3294	11856	27	53	4654	0.0013	40	0.0021	7.89	9.174e-03
SRF	MADS	vertebrate	17.965	535	14615	7	73	583	0.0003	7	0.0005	7.188	2.363e-02

e2f2, 100 up-regulated genes, -2000 to 2000 bp, weakly over-represented, E2F1

Promoter Length		Gene Number 100	200	400
up	2000 bp	N	W	W
	4000 bp	W	W	W
	7000 bp	W	W	W
	10000 bp	W	W	W
down	2000 bp	N	N	N
	4000 bp	N	N	N
	7000 bp	N	N	N
	10000 bp	N	N	N

e2f2

17. The output of oPOSSUM for E-GEOD-2527

TF	TF Class	TF Supergroup	IC	Background gene hits	Background gene non-hits	Target gene hits	Target gene non-hits	Background TFBS hits	Background TFBS rate	Target TFBS hits	Target TFBS rate	Z-score	Fisher score
MYC-MAX	bHLH-ZIP	vertebrate	14.237	828	14322	20	124	886	0.0003	20	0.0006	8.868	1.339e-04
REL	REL	vertebrate	10.515	3609	11541	49	95	4965	0.0018	82	0.0023	8.021	3.806e-03
STAT1	Stat	vertebrate	18.431	417	14733	10	134	436	0.0002	10	0.0004	7.249	7.190e-03
HNF1A	HOMEO	vertebrate	15.548	459	14691	7	137	496	0.0002	11	0.0004	7.174	1.508e-01
Fos	bZIP	vertebrate	10.670	5773	9377	69	75	9489	0.0027	141	0.0032	5.842	1.065e-02
NR3C1	NUCLEAR RECEPTOR	vertebrate	14.749	178	14972	4	140	180	0.0001	4	0.0002	4.881	9.314e-02
Gfi	ZN-FINGER, C2H2	vertebrate	9.470	6037	9113	73	71	10976	0.0039	154	0.0044	4.573	5.594e-03
Foxa2	FORKHEAD	vertebrate	12.433	3625	11525	43	101	5513	0.0024	79	0.0027	4.23	6.168e-02
TAL1-TCF3	bHLH	vertebrate	14.070	1503	13647	24	120	1783	0.0008	28	0.0010	4.183	8.375e-03
MAX	bHLH-ZIP	vertebrate	12.685	2474	12676	38	106	3097	0.0011	46	0.0013	3.704	1.548e-03

gata-1, 200 up-regulated genes, -2000 to 2000 bp, not over-represented

TF	TF Class	TF Supergroup	IC	Background gene hits	Background gene non-hits	Target gene hits	Target gene non-hits	Background TFBS hits	Background TFBS rate	Target TFBS hits	Target TFBS rate	Z-score	Fisher score
T	T-BOX	vertebrate	17.863	195	14955	6	133	199	0.0001	6	0.0002	8.758	1.016e-02
IRF1	TRP-CLUSTER	vertebrate	16.008	1283	13867	22	117	1449	0.0006	25	0.0010	8.443	3.417e-03
STAT1	Stat	vertebrate	18.431	417	14733	9	130	436	0.0002	9	0.0004	7.53	1.586e-02
MEF2A	MADS	vertebrate	15.709	1740	13410	22	117	2190	0.0008	34	0.0011	7.003	7.618e-02
PPARG-RXRA	NUCLEAR RECEPTOR	vertebrate	23.449	65	15085	2	137	65	0.0000	2	0.0001	6.911	1.240e-01
Hand1-Tcfe2a	bHLH	vertebrate	10.144	5274	9876	58	81	8336	0.0030	104	0.0035	5.172	5.466e-02
ELK1	ETS	vertebrate	8.812	6419	8731	59	80	11166	0.0040	134	0.0045	4.451	5.254e-01
MZF1_1-4	ZN-FINGER, C2H2	vertebrate	8.586	10908	4242	112	27	43933	0.0094	504	0.0102	4.224	1.360e-02
TLX1-NFIC	HOMEO/CAAT	vertebrate	19.665	146	15004	3	136	148	0.0001	3	0.0001	4.152	1.542e-01
TEAD1	TEA	vertebrate	15.678	997	14153	13	126	1114	0.0005	16	0.0006	4.15	1.294e-01

gata-1, 200 down-regulated genes, -2000 to 2000 bp, not over-represented

Promoter Length		Gene Number		
		100	200	400
up	2000 bp	N	N	N
	4000 bp	N	N	N
	7000 bp	N	N	N
	10000 bp	N	N	N
down	2000 bp	N	N	N
	4000 bp	N	N	N
	7000 bp	N	N	N
	10000 bp	N	N	N

gata-1

18. The output of oPOSSUM for E-MEXP-634

TF	TF Class	TF Supergroup	IC	Background gene hits	Background gene non-hits	Target gene hits	Target gene non-hits	Background TFBS hits	Background TFBS rate	Target TFBS hits	Target TFBS rate	Z-score	Fisher score
IRF1	TRP-CLUSTER	vertebrate	16.008	1283	13867	15	115	1449	0.0006	18	0.0009	5.553	1.384e-01
NFYA	CAAT-BOX	vertebrate	12.925	2552	12598	26	104	3490	0.0020	36	0.0024	4.605	1.986e-01
MAX	bHLH-ZIP	vertebrate	12.685	2474	12676	24	106	3097	0.0011	32	0.0013	3.448	2.898e-01
TAL1-TCF3	bHLH	vertebrate	14.070	1503	13647	14	116	1783	0.0008	19	0.0010	3.367	4.159e-01
Staf	ZN-FINGER, C2H2	vertebrate	17.541	312	14838	4	126	324	0.0002	4	0.0003	3.28	2.823e-01
NKX3-1	HOMEO	vertebrate	11.127	5231	9919	44	86	10063	0.0025	97	0.0029	3.232	5.973e-01
Prrx2	HOMEO	vertebrate	9.063	8566	6584	76	54	28308	0.0051	262	0.0055	3.032	3.641e-01
STAT1	Stat	vertebrate	18.431	417	14733	5	125	436	0.0002	5	0.0003	2.436	2.902e-01
FOXF2	FORKHEAD	vertebrate	14.824	701	14449	6	124	777	0.0004	8	0.0005	1.967	5.613e-01
ELF5	ETS	vertebrate	8.693	9474	5676	76	54	23856	0.0077	212	0.0080	1.886	8.521e-01

gata-3, 200 up-regulated genes, -2000 to 2000 bp, not over-represented

TF	TF Class	TF Supergroup	IC	Background gene hits	Background gene non-hits	Target gene hits	Target gene non-hits	Background TFBS hits	Background TFBS rate	Target TFBS hits	Target TFBS rate	Z-score	Fisher score
MAX	bHLH-ZIP	vertebrate	12.685	2474	12676	43	89	3097	0.0011	62	0.0019	13.38	3.805e-06
NHLH1	bHLH	vertebrate	14.132	1272	13878	26	106	1468	0.0006	32	0.0012	12.25	4.033e-05
STAT1	Stat	vertebrate	18.431	417	14733	12	120	436	0.0002	12	0.0005	11.29	3.431e-04
NR3C1	NUCLEAR RECEPTOR	vertebrate	14.749	178	14972	5	127	180	0.0001	6	0.0003	11.24	2.131e-02
CREB1	bZIP	vertebrate	12.605	1777	13373	34	98	2042	0.0009	39	0.0014	10.56	7.886e-06
Ar	NUCLEAR RECEPTOR	vertebrate	15.703	40	15110	2	130	40	0.0000	2	0.0001	10.3	5.093e-02
IRF2	TRP-CLUSTER	vertebrate	21.134	74	15076	3	129	74	0.0000	3	0.0002	9.558	2.899e-02
SPIB	ETS	vertebrate	9.060	10919	4231	110	22	35925	0.0090	496	0.0105	9.481	1.868e-03
ELF5	ETS	vertebrate	8.693	9474	5676	105	27	23856	0.0077	330	0.0090	8.867	2.094e-05
Foxq1	FORKHEAD	vertebrate	14.070	1761	13389	28	104	2254	0.0009	39	0.0013	8.016	1.230e-03

gata-3, 200 down-regulated genes, -2000 to 2000 bp, not over-represented

		Gene Number		
Promoter Length		100	200	400
up	2000 bp	N	N	N
	4000 bp	N	N	N
	7000 bp	N	N	N
	10000 bp	N	N	N
down	2000 bp	N	N	N
	4000 bp	N	N	N
	7000 bp	N	N	N
	10000 bp	N	N	N

gata-3

19. The output of oPOSSUM for E-GEOD-5823

TF	TF Class	TF Supergroup	IC	Background gene hits	Background gene non-hits	Target gene hits	Target gene non-hits	Background TFBS hits	Background TFBS rate	Target TFBS hits	Target TFBS rate	Z-score	Fisher score
SP1	ZN-FINGER, C2H2	vertebrate	9.719	9932	5218	111	48	35108	0.0125	429	0.0163	17.48	1.491e-01
NFYA	CAAT-BOX	vertebrate	12.925	4645	10505	52	107	7556	0.0043	104	0.0063	15.77	3.163e-01
Arnt-Ahr	bHLH	vertebrate	9.532	11906	3244	123	36	55098	0.0118	651	0.0149	14.55	6.868e-01
Staf	ZN-FINGER, C2H2	vertebrate	17.541	1282	13868	22	137	1497	0.0011	25	0.0019	13.06	1.593e-02
E2F1	E2F_TDP	vertebrate	13.838	3294	11856	47	112	4654	0.0013	73	0.0022	12.55	1.339e-02
NR3C1	NUCLEAR RECEPTOR	vertebrate	14.749	1835	13315	26	133	2162	0.0014	33	0.0023	11.97	7.027e-02
MYC-MAX	bHLH-ZIP	vertebrate	14.237	2630	12520	34	125	3253	0.0013	50	0.0021	11.68	1.118e-01
MAX	bHLH-ZIP	vertebrate	12.685	6081	9069	66	93	10424	0.0037	133	0.0051	11.28	3.920e-01
ELK1	ETS	vertebrate	8.812	11197	3953	116	43	36793	0.0131	406	0.0155	10.43	6.461e-01
MZF1_5-13	ZN-FINGER, C2H2	vertebrate	9.400	10713	4437	108	51	40747	0.0146	446	0.0170	10.4	8.049e-01

cMyc, 200 up-regulated genes, -2000 to 2000 bp, weakly over-represented

TF	TF Class	TF Supergroup	IC	Background gene hits	Background gene non-hits	Target gene hits	Target gene non-hits	Background TFBS hits	Background TFBS rate	Target TFBS hits	Target TFBS rate	Z-score	Fisher score
IRF2	TRP-CLUSTER	vertebrate	21.134	552	14598	15	118	588	0.0004	15	0.0010	15.89	1.234e-04
CREB1	bZIP	vertebrate	12.605	4951	10199	57	76	7418	0.0032	102	0.0044	11.15	9.223e-03
REL	REL	vertebrate	10.515	7832	7318	85	48	16270	0.0058	204	0.0073	10.2	3.098e-03
FOXF2	FORKHEAD	vertebrate	14.824	2629	12521	39	94	3571	0.0018	52	0.0026	10.15	4.925e-04
RELA	REL	vertebrate	14.757	4606	10544	52	81	6841	0.0024	94	0.0034	9.738	2.064e-02
NF-kappaB	REL	vertebrate	13.345	5874	9276	64	69	10146	0.0036	128	0.0046	8.286	1.807e-02
Ar	NUCLEAR RECEPTOR	vertebrate	15.703	376	14774	6	127	395	0.0003	7	0.0005	7.129	1.166e-01
NFKB1	REL	vertebrate	15.627	2687	12463	32	101	3691	0.0015	50	0.0020	7.097	4.088e-02
IRF1	TRP-CLUSTER	vertebrate	16.008	4150	11000	59	74	6213	0.0027	78	0.0033	6.927	2.218e-05
STAT1	Stat	vertebrate	18.431	1802	13348	25	108	2137	0.0011	29	0.0014	6.125	1.413e-02

cMyc, 200 down-regulated genes, -5000 to 2000 bp, weakly over-represented

Promoter Length		Gene Number 100	200	400
up	7000bp	W	W	W
	4000bp	W	W	W
	2000bp	N	N	W
down	7000bp	N	N	W
	4000bp	N	N	W
	2000bp	W	W	W

cMyc

20. The output of oPOSSUM for E-GEOD-7137

TF	TF Class	TF Supergroup	IC	Background gene hits	Background gene non-hits	Target gene hits	Target gene non-hits	Background TFBS hits	Background TFBS rate	Target TFBS hits	Target TFBS rate	Z-score	Fisher score
NFYA	CAAT-BOX	vertebrate	12.925	2552	12598	43	95	3490	0.0020	65	0.0046	27.36	2.865e-05
HNF4A	NUCLEAR RECEPTOR	vertebrate	9.617	1637	13513	23	115	1852	0.0009	27	0.0015	10.98	2.422e-02
HNF1A	HOMEO	vertebrate	15.548	459	14691	9	129	496	0.0002	9	0.0006	9.14	2.609e-02
SP1	ZN-FINGER, C2H2	vertebrate	9.719	5559	9591	56	82	10226	0.0037	108	0.0047	8.482	1.960e-01
MYC-MAX	bHLH-ZIP	vertebrate	14.237	828	14322	12	126	886	0.0003	13	0.0006	7.056	7.737e-02
USF1	bHLH-ZIP	vertebrate	11.290	3565	11585	37	101	4939	0.0012	56	0.0017	6.506	2.092e-01
TLX1-NFIC	HOMEO/CAAT	vertebrate	19.665	146	15004	3	135	148	0.0001	3	0.0002	5.977	1.519e-01
Arnt	bHLH	vertebrate	10.992	2673	12477	28	110	3594	0.0008	41	0.0011	5.236	2.384e-01
TAL1-TCF3	bHLH	vertebrate	14.070	1503	13647	15	123	1783	0.0008	20	0.0011	4.906	3.961e-01
MAX	bHLH-ZIP	vertebrate	12.685	2474	12676	29	109	3097	0.0011	33	0.0014	4.824	8.907e-02

KLF15, 200 up-regulated genes, -2000 to 2000 bp, not over-represented

TF	TF Class	TF Supergroup	IC	Background gene hits	Background gene non-hits	Target gene hits	Target gene non-hits	Background TFBS hits	Background TFBS rate	Target TFBS hits	Target TFBS rate	Z-score	Fisher score
NR2F1	NUCLEAR RECEPTOR	vertebrate	15.924	696	14454	12	139	731	0.0004	13	0.0007	9.207	4.769e-02
HNF4A	NUCLEAR RECEPTOR	vertebrate	9.617	1637	13513	24	127	1852	0.0009	26	0.0013	8.067	3.590e-02
MAX	bHLH-ZIP	vertebrate	12.685	2474	12676	34	117	3097	0.0011	41	0.0016	7.679	3.026e-02
STAT1	Stat	vertebrate	18.431	417	14733	7	144	436	0.0002	8	0.0004	7.546	1.268e-01
Mycn	bHLH-ZIP	vertebrate	10.443	7434	7716	80	71	15024	0.0032	162	0.0038	5.393	1.909e-01
TLX1-NFIC	HOMEO/CAAT	vertebrate	19.665	146	15004	3	148	148	0.0001	3	0.0002	5.239	1.824e-01
GABPA	ETS	vertebrate	13.890	2256	12894	29	122	2742	0.0010	33	0.0013	5.127	8.911e-02
HLF	bZIP	vertebrate	11.147	1294	13856	16	135	1495	0.0006	17	0.0008	3.199	2.208e-01
T	T-BOX	vertebrate	17.863	195	14955	3	148	199	0.0001	3	0.0001	2.839	3.107e-01
Arnt	bHLH	vertebrate	10.992	2673	12477	30	121	3594	0.0008	39	0.0009	2.713	2.676e-01

KLF15, 200 down-regulated genes, -2000 to 2000 bp, not over-represented

Promoter Length		Gene Number		
		100	200	400
up	2000 bp	N	N	N
	4000 bp	N	N	N
	7000 bp	N	N	N
	10000 bp	N	N	N
down	2000 bp	N	N	N
	4000 bp	N	N	N
	7000 bp	N	N	N
	10000 bp	N	N	N

KLF15

21. The output of oPOSSUM for E-GEOD-5800

TF	TF Class	TF Supergroup	IC	Background gene hits	Background gene non-hits	Target gene hits	Target gene non-hits	Background TFBS hits	Background TFBS rate	Target TFBS hits	Target TFBS rate	Z-score	Fisher score
Ar	NUCLEAR RECEPTOR	vertebrate	15.703	376	14774	7	110	395	0.0003	8	0.0007	10.24	2.799e-02
Fos	bZIP	vertebrate	10.670	9453	5697	92	25	24913	0.0071	272	0.0082	6.549	1.307e-04
NR2F1	NUCLEAR RECEPTOR	vertebrate	15.924	2872	12278	33	84	3587	0.0018	43	0.0023	5.724	1.002e-02
TEAD1	TEA	vertebrate	15.678	3102	12048	38	79	4137	0.0018	49	0.0022	5.363	1.655e-03
HLF	bZIP	vertebrate	11.147	4612	10538	53	64	7195	0.0031	81	0.0037	5.319	5.301e-04
Cebpa	bZIP	vertebrate	9.187	7775	7375	79	38	18051	0.0077	191	0.0086	5.215	2.951e-04
NR3C1	NUCLEAR RECEPTOR	vertebrate	14.749	1835	13315	18	99	2162	0.0014	26	0.0018	5.11	1.728e-01
RORA_1	NUCLEAR RECEPTOR	vertebrate	13.190	6302	8848	59	58	11069	0.0040	120	0.0045	4.603	3.387e-02
RORA_2	NUCLEAR RECEPTOR	vertebrate	17.425	1858	13292	22	95	2200	0.0011	26	0.0014	4.165	2.750e-02
REST	ZN-FINGER, C2H2	vertebrate	22.958	190	14960	3	114	202	0.0001	3	0.0002	3.326	1.846e-01

IRF6, 200 up-regulated genes, -2000 to 2000 bp, not over-represented

TF	TF Class	TF Supergroup	IC	Background gene hits	Background gene non-hits	Target gene hits	Target gene non-hits	Background TFBS hits	Background TFBS rate	Target TFBS hits	Target TFBS rate	Z-score	Fisher score
NF-kappaB	REL	vertebrate	13.345	5874	9276	62	55	10146	0.0036	131	0.0048	10.45	1.310e-03
RELA	REL	vertebrate	14.757	4606	10544	50	67	6841	0.0024	90	0.0033	9.216	3.296e-03
Myf	bHLH	vertebrate	15.914	7082	8068	62	55	14436	0.0062	167	0.0074	7.976	1.047e-01
REL	REL	vertebrate	10.515	7832	7318	74	43	16270	0.0058	189	0.0070	7.926	7.942e-03
Pax6	PAIRED	vertebrate	13.798	693	14457	11	106	753	0.0004	13	0.0007	7.859	1.920e-02
Pax4	PAIRED-HOMEO	vertebrate	11.004	145	15005	3	114	150	0.0002	3	0.0003	6.956	1.050e-01
Spz1	bHLH-ZIP	vertebrate	11.907	4976	10174	48	69	7849	0.0031	93	0.0038	6.452	3.945e-02
SPIB	ETS	vertebrate	9.060	13831	1319	114	3	118041	0.0295	1214	0.0314	5.614	7.206e-03
sna	ZN-FINGER, C2H2	insect	10.706	13063	2087	108	9	85612	0.0184	889	0.0197	5.13	3.159e-02
SRF	MADS	vertebrate	17.965	535	14615	7	110	583	0.0003	9	0.0004	4.829	1.227e-01

IRF6, 200 down-regulated genes, -2000 to 2000 bp, not over-represented

Promoter Length		Gene Number		
		100	200	400
up	7000bp	W	N	N
	4000bp	W	N	W
	2000bp	N	N	N
down	7000bp	N	N	N
	4000bp	N	N	N
	2000bp	N	N	N

IRF6

22. The output of oPOSSUM for E-GEOD-2624

TF	TF Class	TF Supergroup	IC	Background gene hits	Background gene non-hits	Target gene hits	Target gene non-hits	Background TFBS hits	Background TFBS rate	Target TFBS hits	Target TFBS rate	Z-score	Fisher score
Nkx2-5	HOMEO	vertebrate	8.270	12616	2534	141	19	96540	0.0241	1627	0.0292	20.6	5.844e-02
Prrx2	HOMEO	vertebrate	9.063	11710	3440	130	30	80966	0.0145	1394	0.0179	17.84	1.361e-01
Sox5	HMG	vertebrate	10.831	11265	3885	129	31	57233	0.0143	986	0.0177	17.8	4.009e-02
SRY	HMG	vertebrate	9.193	11001	4149	131	29	53158	0.0171	897	0.0207	17.4	4.467e-03
Pdx1	HOMEO	vertebrate	9.040	12302	2848	135	25	86351	0.0185	1398	0.0215	13.9	1.793e-01
Foxa2	FORKHEAD	vertebrate	12.433	7940	7210	102	58	21593	0.0093	370	0.0114	13.88	2.580e-03
Lhx3	HOMEO	vertebrate	12.941	7325	7825	104	56	21103	0.0068	370	0.0085	13.34	1.780e-05
FOXI1	FORKHEAD	vertebrate	13.183	8038	7112	104	56	22048	0.0095	370	0.0114	12.48	1.554e-03
SOX9	HMG	vertebrate	9.079	8524	6626	109	51	22208	0.0071	380	0.0088	12.08	1.500e-03
FOXD1	FORKHEAD	vertebrate	11.926	7400	7750	95	65	16369	0.0047	288	0.0059	11.25	5.006e-03

NF-kb, 200 up-regulated genes, -2000 to 2000 bp, not over-represented

TF	TF Class	TF Supergroup	IC	Background gene hits	Background gene non-hits	Target gene hits	Target gene non-hits	Background TFBS hits	Background TFBS rate	Target TFBS hits	Target TFBS rate	Z-score	Fisher score
MZF1_1-4	ZN-FINGER, C2H2	vertebrate	8.586	13837	1313	147	5	154947	0.0332	1974	0.0376	13.85	7.697e-03
IRF2	TRP-CLUSTER	vertebrate	21.134	552	14598	14	138	588	0.0004	15	0.0009	13.8	1.536e-03
SP1	ZN-FINGER, C2H2	vertebrate	9.719	9932	5218	108	44	35108	0.0125	478	0.0152	13.34	8.995e-02
MZF1_5-13	ZN-FINGER, C2H2	vertebrate	9.400	10713	4437	121	31	40747	0.0146	541	0.0172	12.33	8.803e-03
NFKB1	REL	vertebrate	15.627	2687	12463	41	111	3691	0.0015	64	0.0022	11.57	3.214e-03
ZEB1	ZN-FINGER, C2H2	vertebrate	8.305	14221	929	146	6	180543	0.0387	2201	0.0420	9.495	1.720e-01
NF-kappaB	REL	vertebrate	13.345	5874	9276	72	80	10146	0.0036	143	0.0045	8.569	1.952e-02
T	T-BOX	vertebrate	17.863	1006	14144	19	133	1111	0.0004	21	0.0007	7.945	6.274e-03
REL	REL	vertebrate	10.515	7832	7318	95	57	16270	0.0058	214	0.0068	7.279	4.880e-03
ZNF354C	ZN-FINGER, C2H2	vertebrate	8.958	13853	1297	144	8	136176	0.0292	1643	0.0313	7.117	9.036e-02

NF-kb, 200 down-regulated genes, -2000 to 2000 bp, significantly over-represented

Promoter Length		Gene Number		
		100	200	400
up	7000bp	N	N	N
	4000bp	N	N	N
	2000bp	N	N	N
down	7000bp	N	S	S
	4000bp	N	S	S
	2000bp	W	S	S

NF-kb

23. The output of oPOSSUM for E-GEOD-3116

TF	TF Class	TF Supergroup	IC	Background gene hits	Background gene non-hits	Target gene hits	Target gene non-hits	Background TFBS hits	Background TFBS rate	Target TFBS hits	Target TFBS rate	Z-score	Fisher score
HNF4A	NUCLEAR RECEPTOR	vertebrate	9.617	5180	9970	69	70	7908	0.0037	112	0.0058	17.59	1.345e-04
NR2F1	NUCLEAR RECEPTOR	vertebrate	15.924	2872	12278	36	103	3587	0.0018	53	0.0030	13.72	2.780e-02
HNF1A	HOMEO	vertebrate	15.548	1921	13229	25	114	2495	0.0012	32	0.0018	7.581	4.568e-02
RORA_2	NUCLEAR RECEPTOR	vertebrate	17.425	1858	13292	26	113	2200	0.0011	26	0.0014	5.247	1.902e-02
TLX1-NFIC	HOMEO/CAAT	vertebrate	19.665	534	14616	8	131	562	0.0003	8	0.0004	4.87	1.211e-01
NR3C1	NUCLEAR RECEPTOR	vertebrate	14.749	1835	13315	20	119	2162	0.0014	22	0.0016	2.483	2.405e-01
ESR1	NUCLEAR RECEPTOR	vertebrate	17.683	349	14801	3	136	365	0.0002	4	0.0003	1.636	6.242e-01
PPARG-RXRA	NUCLEAR RECEPTOR	vertebrate	23.449	272	14878	3	136	279	0.0002	3	0.0002	1.334	4.580e-01
SRF	MADS	vertebrate	17.965	535	14615	5	134	583	0.0003	6	0.0003	1.103	5.471e-01
T	T-BOX	vertebrate	17.863	1006	14144	11	128	1111	0.0004	11	0.0005	1.037	3.198e-01

HNF4a, 200 up-regulated genes, -2000 to 2000 bp, significantly over-represented

TF	TF Class	TF Supergroup	IC	Background gene hits	Background gene non-hits	Target gene hits	Target gene non-hits	Background TFBS hits	Background TFBS rate	Target TFBS hits	Target TFBS rate	Z-score	Fisher score
Foxd3	FORKHEAD	vertebrate	12.945	8235	6915	72	43	25203	0.0108	309	0.0144	17.76	4.614e-02
SRY	HMG	vertebrate	9.193	11001	4149	89	26	53158	0.0171	614	0.0215	17.24	1.488e-01
Gfi	ZN-FINGER, C2H2	vertebrate	9.470	9993	5157	87	28	31256	0.0112	376	0.0146	16.7	1.656e-02
Pdx1	HOMEO	vertebrate	9.040	12302	2848	98	17	86351	0.0185	967	0.0226	15.28	1.639e-01
Foxa2	FORKHEAD	vertebrate	12.433	7940	7210	67	48	21593	0.0093	260	0.0121	15.25	1.233e-01
Sox5	HMG	vertebrate	10.831	11265	3885	90	25	57233	0.0143	652	0.0178	14.7	1.995e-01
Sox17	HMG	vertebrate	10.502	11132	4018	90	25	41756	0.0134	475	0.0166	14.12	1.459e-01
FOXI1	FORKHEAD	vertebrate	13.183	8038	7112	71	44	22048	0.0095	257	0.0120	13.34	3.820e-02
Nkx2-5	HOMEO	vertebrate	8.270	12616	2534	101	14	96540	0.0241	1034	0.0282	13.27	1.166e-01
Nobox	HOMEO	vertebrate	9.573	10484	4666	90	25	42827	0.0122	482	0.0150	12.73	2.052e-02

HNF4a, 200 down-regulated genes, -2000 to 2000 bp, not over-represented

Promoter Length		Gene Number 100	200	400
up	7000bp	S	S	S
	4000bp	S	S	S
	2000bp	S	S	S
down	7000bp	N	N	N
	4000bp	N	N	N
	2000bp	N	N	N

HNF4a

24. The output of oPOSSUM for E-GEOD-5424

TF	TF Class	TF Supergroup	IC	Background gene hits	Background gene non-hits	Target gene hits	Target gene non-hits	Background TFBS hits	Background TFBS rate	Target TFBS hits	Target TFBS rate	Z-score	Fisher score
Pax5	PAIRED	vertebrate	12.432	1352	13798	23	107	1534	0.0011	31	0.0024	20.11	1.239e-03
NHLH1	bHLH	vertebrate	14.132	4061	11089	50	80	5936	0.0025	74	0.0035	9.106	2.582e-03
NR3C1	NUCLEAR RECEPTOR	vertebrate	14.749	1835	13315	21	109	2162	0.0014	28	0.0020	7.713	1.050e-01
MZF1_1-4	ZN-FINGER, C2H2	vertebrate	8.586	13837	1313	124	6	154947	0.0332	1537	0.0359	7.434	6.075e-02
REST	ZN-FINGER, C2H2	vertebrate	22.958	190	14960	4	126	202	0.0001	4	0.0003	6.772	8.385e-02
Roaz	ZN-FINGER, C2H2	vertebrate	17.925	4153	10997	47	83	6324	0.0034	70	0.0041	6.022	1.870e-02
Prrx2	HOMEO	vertebrate	9.063	11710	3440	108	22	80966	0.0145	812	0.0158	5.585	6.831e-02
ELK1	ETS	vertebrate	8.812	11197	3953	107	23	36793	0.0131	370	0.0144	5.494	1.632e-02
SOX9	HMG	vertebrate	9.079	8524	6626	88	42	22208	0.0071	228	0.0080	5.016	5.276e-03
IRF2	TRP-CLUSTER	vertebrate	21.134	552	14598	8	122	588	0.0004	8	0.0006	4.686	1.051e-01

Foxa, 200 up-regulated genes, -2000 to 2000 bp, weakly over-represented

TF	TF Class	TF Supergroup	IC	Background gene hits	Background gene non-hits	Target gene hits	Target gene non-hits	Background TFBS hits	Background TFBS rate	Target TFBS hits	Target TFBS rate	Z-score	Fisher score
SRY	HMG	vertebrate	9.193	11001	4149	88	26	53158	0.0171	472	0.0202	11.02	1.618e-01
Nkx2-5	HOMEO	vertebrate	8.270	12616	2534	102	12	96540	0.0241	833	0.0278	10.75	4.446e-02
CREB1	bZIP	vertebrate	12.605	4951	10199	41	73	7418	0.0032	77	0.0044	9.882	2.575e-01
Evi1	ZN-FINGER, C2H2	vertebrate	17.909	843	14307	13	101	949	0.0005	14	0.0009	9.587	1.140e-02
Sox5	HMG	vertebrate	10.831	11265	3885	91	23	57233	0.0143	498	0.0166	8.764	1.084e-01
NFYA	CAAT-BOX	vertebrate	12.925	4645	10505	37	77	7556	0.0043	72	0.0055	8.106	3.730e-01
Pdx1	HOMEO	vertebrate	9.040	12302	2848	92	22	86351	0.0185	731	0.0209	8.012	6.099e-01
HNF1A	HOMEO	vertebrate	15.548	1921	13229	20	94	2495	0.0012	28	0.0019	7.984	8.314e-02
SRF	MADS	vertebrate	17.965	535	14615	5	109	583	0.0003	9	0.0005	7.585	3.777e-01
Prrx2	HOMEO	vertebrate	9.063	11710	3440	89	25	80966	0.0145	691	0.0164	7.576	4.742e-01

Foxa, 200 up-regulated genes, -2000 to 2000 bp, not over-represented

Promoter Length		Gene Number 100	200	400
up	7000bp	N	W	S
	4000bp	W	W	W
	2000bp	N	N	W
down	7000bp	W	W	W
	4000bp	N	N	N
	2000bp	N	W	N

Foxa

25. The output of oPOSSUM for E-GEOD-8943

TF	TF Class	TF Supergroup	IC	Background gene hits	Background gene non-hits	Target gene hits	Target gene non-hits	Background TFBS hits	Background TFBS rate	Target TFBS hits	Target TFBS rate	Z-score	Fisher score
Foxd3	FORKHEAD	vertebrate	12.945	3468	11682	40	101	5452	0.0023	72	0.0033	10.17	7.741e-02
STAT1	Stat	vertebrate	18.431	417	14733	8	133	436	0.0002	8	0.0004	7.196	4.326e-02
MEF2A	MADS	vertebrate	15.709	1740	13410	21	120	2190	0.0008	30	0.0011	6.608	1.307e-01
Lhx3	HOMEO	vertebrate	16.354	1024	14126	13	128	1222	0.0006	17	0.0008	5.893	1.604e-01
NFIL3	bZIP	vertebrate	14.139	1615	13535	18	123	1996	0.0008	26	0.0011	5.586	2.456e-01
Fos	bZIP	vertebrate	10.670	5773	9377	59	82	9489	0.0027	107	0.0033	5.452	2.048e-01
TEAD1	TEA	vertebrate	15.678	997	14153	14	127	1114	0.0005	15	0.0007	4.865	8.306e-02
NR2F1	NUCLEAR RECEPTOR	vertebrate	15.924	696	14454	8	133	731	0.0004	10	0.0005	4.469	3.238e-01
Lhx3	HOMEO	vertebrate	12.941	4014	11136	40	101	7208	0.0023	79	0.0027	4.202	3.380e-01
Foxa2	FORKHEAD	vertebrate	12.433	3625	11525	38	103	5513	0.0024	60	0.0027	4.04	2.279e-01

Foxq, 200 up-regulated genes, -2000 to 2000 bp, weakly over-represented

TF	TF Class	TF Supergroup	IC	Background gene hits	Background gene non-hits	Target gene hits	Target gene non-hits	Background TFBS hits	Background TFBS rate	Target TFBS hits	Target TFBS rate	Z-score	Fisher score
Pax4	PAIRED-HOMEO	vertebrate	11.004	145	15005	3	148	150	0.0002	3	0.0003	6.72	1.800e-01
MZF1_5-13	ZN-FINGER, C2H2	vertebrate	9.400	10713	4437	116	35	40747	0.0146	446	0.0161	6.648	5.796e-02
GABPA	ETS	vertebrate	13.890	5358	9792	61	90	8422	0.0030	101	0.0036	6.047	1.155e-01
SRF	MADS	vertebrate	17.965	535	14615	6	145	583	0.0003	10	0.0004	6.02	4.446e-01
MYC-MAX	bHLH-ZIP	vertebrate	14.237	2630	12520	32	119	3253	0.0013	42	0.0017	5.669	1.306e-01
ELK1	ETS	vertebrate	8.812	11197	3953	120	31	36793	0.0131	396	0.0143	5.192	7.003e-02
SPIB	ETS	vertebrate	9.060	13831	1319	141	10	118041	0.0295	1221	0.0308	3.97	2.288e-01
STAT1	Stat	vertebrate	18.431	1802	13348	22	129	2137	0.0011	26	0.0013	3.884	1.865e-01
ELF5	ETS	vertebrate	8.693	12664	2486	132	19	63365	0.0204	660	0.0214	3.832	1.222e-01
IRF2	TRP-CLUSTER	vertebrate	21.134	552	14598	8	143	588	0.0004	8	0.0005	3.765	1.894e-01

Foxq, 200 down-regulated genes, -2000 to 2000 bp, not over-represented

Promoter Length		Gene Number		
		100	200	400
up	7000bp	N	W	N
	4000bp	N	W	N
	2000bp	N	N	N
down	7000bp	N	N	W
	4000bp	N	N	N
	2000bp	N	N	N

Foxq

26. The output of oPOSSUM for E-GEOD-11557

TF	TF Class	TF Supergroup	IC	Background gene hits	Background gene non-hits	Target gene hits	Target gene non-hits	Background TFBS hits	Background TFBS rate	Target TFBS hits	Target TFBS rate	Z-score	Fisher score
Foxd3	FORKHEAD	vertebrate	12.945	3468	11682	40	101	5452	0.0023	72	0.0033	10.17	7.741e-02
STAT1	Stat	vertebrate	18.431	417	14733	8	133	436	0.0002	8	0.0004	7.196	4.326e-02
MEF2A	MADS	vertebrate	15.709	1740	13410	21	120	2190	0.0008	30	0.0011	6.608	1.307e-01
Lhx3	HOMEO	vertebrate	16.354	1024	14126	13	128	1222	0.0006	17	0.0008	5.893	1.604e-01
NFIL3	bZIP	vertebrate	14.139	1615	13535	18	123	1996	0.0008	26	0.0011	5.586	2.456e-01
Fos	bZIP	vertebrate	10.670	5773	9377	59	82	9489	0.0027	107	0.0033	5.452	2.048e-01
TEAD1	TEA	vertebrate	15.678	997	14153	14	127	1114	0.0005	15	0.0007	4.865	8.306e-02
NR2F1	NUCLEAR RECEPTOR	vertebrate	15.924	696	14454	8	133	731	0.0004	10	0.0005	4.469	3.238e-01
Lhx3	HOMEO	vertebrate	12.941	4014	11136	40	101	7208	0.0023	79	0.0027	4.202	3.380e-01
Foxa2	FORKHEAD	vertebrate	12.433	3625	11525	38	103	5513	0.0024	60	0.0027	4.04	2.279e-01

evi1, 200 up-regulated genes, -2000 to 2000 bp, not over-represented

TF	TF Class	TF Supergroup	IC	Background gene hits	Background gene non-hits	Target gene hits	Target gene non-hits	Background TFBS hits	Background TFBS rate	Target TFBS hits	Target TFBS rate	Z-score	Fisher score
Nkx2-5	HOMEO	vertebrate	8.270	12616	2534	105	7	96540	0.0241	1433	0.0313	26.36	8.938e-04
SRY	HMG	vertebrate	9.193	11001	4149	94	18	53158	0.0171	817	0.0229	25.52	3.605e-03
Pdx1	HOMEO	vertebrate	9.040	12302	2848	106	6	86351	0.0185	1281	0.0240	22.95	3.740e-05
Foxd3	FORKHEAD	vertebrate	12.945	8235	6915	87	25	25203	0.0108	395	0.0148	21.79	2.799e-07
Foxa2	FORKHEAD	vertebrate	12.433	7940	7210	90	22	21593	0.0093	345	0.0129	21.61	8.199e-10
Prrx2	HOMEO	vertebrate	9.063	11710	3440	105	7	80966	0.0145	1217	0.0190	21.42	2.762e-06
Sox5	HMG	vertebrate	10.831	11265	3885	97	15	57233	0.0143	860	0.0188	21.28	1.262e-03
Lhx3	HOMEO	vertebrate	12.941	7325	7825	83	29	21103	0.0068	344	0.0097	19.8	2.825e-08
FOXF2	FORKHEAD	vertebrate	14.824	2629	12521	44	68	3571	0.0018	74	0.0032	19.36	4.035e-08
Foxq1	FORKHEAD	vertebrate	14.070	4804	10346	59	53	8730	0.0034	150	0.0051	16.6	3.756e-06

evi1, 200 down-regulated genes, -2000 to 2000 bp, significantly over-represented

Promoter Length		Gene Number 100	200	400
up	7000bp	N	N	N
	4000bp	N	N	N
	2000bp	N	N	N
down	7000bp	N	W	W
	4000bp	W	S	W
	2000bp	W	S	W

evi1

27. The output of oPOSSUM for E-TABM-43

TF	TF Class	TF Supergroup	IC	Background gene hits	Background gene non-hits	Target gene hits	Target gene non-hits	Background TFBS hits	Background TFBS rate	Target TFBS hits	Target TFBS rate	Z-score	Fisher score
TP53	P53	vertebrate	26.239	18	15132	2	141	18	0.0000	2	0.0001	16.85	1.477e-02
SP1	ZN-FINGER, C2H2	vertebrate	9.719	9932	5218	107	36	35108	0.0125	505	0.0149	12.46	1.135e-02
IRF2	TRP-CLUSTER	vertebrate	21.134	552	14598	13	130	588	0.0004	14	0.0007	10.93	2.479e-03
STAT1	Stat	vertebrate	18.431	1802	13348	28	115	2137	0.0011	40	0.0017	10.41	5.671e-03
Foxa2	FORKHEAD	vertebrate	12.433	7940	7210	91	52	21593	0.0093	305	0.0108	9.475	4.545e-03
Foxd3	FORKHEAD	vertebrate	12.945	8235	6915	93	50	25203	0.0108	351	0.0125	9.249	6.421e-03
SRY	HMG	vertebrate	9.193	11001	4149	119	24	53158	0.0171	716	0.0191	8.77	2.153e-03
NFKB1	REL	vertebrate	15.627	2687	12463	39	104	3691	0.0015	61	0.0020	8.122	3.231e-03
HNF1A	HOMEO	vertebrate	15.548	1921	13229	30	113	2495	0.0012	42	0.0017	8.051	3.835e-03
FOXI1	FORKHEAD	vertebrate	13.183	8038	7112	96	47	22048	0.0095	302	0.0107	7.565	4.660e-04

TP53, 200 up-regulated genes, -2000 to 2000 bp, weakly over-represented

TF	TF Class	TF Supergroup	IC	Background gene hits	Background gene non-hits	Target gene hits	Target gene non-hits	Background TFBS hits	Background TFBS rate	Target TFBS hits	Target TFBS rate	Z-score	Fisher score
MZF1_1-4	ZN-FINGER, C2H2	vertebrate	8.586	13837	1313	153	9	154947	0.0332	1779	0.0426	26.11	9.836e-02
SP1	ZN-FINGER, C2H2	vertebrate	9.719	9932	5218	118	44	35108	0.0125	435	0.0173	21.59	2.989e-02
Arnt-Ahr	bHLH	vertebrate	9.532	11906	3244	142	20	55098	0.0118	663	0.0159	18.76	2.173e-03
SRF	MADS	vertebrate	17.965	535	14615	13	149	583	0.0003	16	0.0008	16.27	5.521e-03
ZNF354C	ZN-FINGER, C2H2	vertebrate	8.958	13853	1297	152	10	136176	0.0292	1441	0.0345	15.7	1.742e-01
Roaz	ZN-FINGER, C2H2	vertebrate	17.925	4153	10997	56	106	6324	0.0034	85	0.0051	14.58	2.809e-02
CREB1	bZIP	vertebrate	12.605	4951	10199	63	99	7418	0.0032	95	0.0045	12.12	5.725e-02
Staf	ZN-FINGER, C2H2	vertebrate	17.541	1282	13868	20	142	1497	0.0011	23	0.0018	11.68	5.811e-02
ZEB1	ZN-FINGER, C2H2	vertebrate	8.305	14221	929	155	7	180543	0.0387	1804	0.0432	11.56	2.192e-01
GABPA	ETS	vertebrate	13.890	5358	9792	66	96	8422	0.0030	107	0.0043	11.48	9.100e-02

TP53, 200 down-regulated genes, -2000 to 2000 bp, not over-represented

Promoter Length		Gene Number 100	200	400
up	7000bp	N	S	S
	4000bp	N	W	W
	2000bp	N	S	W
down	7000bp	N	N	N
	4000bp	N	N	N
	2000bp	N	N	N

TP53

28. The output of oPOSSUM for E-GEOD-2815

TF	TF Class	TF Supergroup	IC	Background gene hits	Background gene non-hits	Target gene hits	Target gene non-hits	Background TFBS hits	Background TFBS rate	Target TFBS hits	Target TFBS rate	Z-score	Fisher score
NFYA	[illegible]-BOX	vertebrate	12.925	4645	10505	67	94	7556	0.0043	122	0.0063	16.53	2.221e-03
CREB1	bZIP	vertebrate	12.605	4951	10199	73	88	7418	0.0032	116	0.0045	12.73	5.853e-04
SP1	ZN-FINGER, C2H2	vertebrate	9.719	9932	5218	117	44	35108	0.0125	463	0.0149	11.59	3.366e-02
MAX	bHLH-ZIP	vertebrate	12.685	6081	9069	77	84	10424	0.0037	154	0.0049	11.14	2.956e-02
GABPA	ETS	vertebrate	13.890	5358	9792	76	85	8422	0.0030	127	0.0041	10.85	1.393e-03
Arnt	bHLH	vertebrate	10.992	9176	5974	111	50	23233	0.0050	329	0.0063	10.72	1.758e-02
USF1	bHLH-ZIP	vertebrate	11.290	8718	6432	106	55	20331	0.0051	282	0.0063	9.791	1.995e-02
MYC-MAX	bHLH-ZIP	vertebrate	14.237	2630	12520	40	121	3253	0.0013	54	0.0019	9.778	1.076e-02
Mycn	bHLH-ZIP	vertebrate	10.443	9036	6114	107	54	22243	0.0048	309	0.0059	9.554	4.605e-02
Myb	TRP-CLUSTER	vertebrate	9.883	10129	5021	114	47	30388	0.0087	386	0.0099	7.345	1.648e-01

Myb, 200 up-regulated genes, -2000 to 2000 bp, not over-represented

TF	TF Class	TF Supergroup	IC	Background gene hits	Background gene non-hits	Target gene hits	Target gene non-hits	Background TFBS hits	Background TFBS rate	Target TFBS hits	Target TFBS rate	Z-score	Fisher score
Foxd3	FORKHEAD	vertebrate	12.945	8235	6915	96	62	25203	0.0108	390	0.0132	13.68	6.281e-02
Nkx2-5	HOMEO	vertebrate	8.270	12616	2534	138	20	96540	0.0241	1390	0.0274	12.65	1.015e-01
Lhx3	HOMEO	vertebrate	12.941	7325	7825	85	73	21103	0.0068	336	0.0085	12.55	9.975e-02
Pdx1	HOMEO	vertebrate	9.040	12302	2848	134	24	86351	0.0185	1254	0.0212	11.84	1.453e-01
Nobox	HOMEO	vertebrate	9.573	10484	4666	118	40	42827	0.0122	635	0.0143	11.19	7.913e-02
Prrx2	HOMEO	vertebrate	9.063	11710	3440	132	26	80966	0.0145	1180	0.0166	10.74	3.476e-02
Pax6	PAIRED	vertebrate	13.798	693	14457	18	140	753	0.0004	18	0.0007	10.19	3.954e-04
Gfi	ZN-FINGER, C2H2	vertebrate	9.470	9993	5157	119	39	31256	0.0112	457	0.0129	9.653	7.397e-03
FOXF2	FORKHEAD	vertebrate	14.824	2629	12521	40	118	3571	0.0018	62	0.0024	9.272	7.751e-03
Ddit3-Cebpa	bZIP	vertebrate	11.652	3628	11522	54	104	5245	0.0022	88	0.0030	9.107	2.528e-03

Myb, 200 down-regulated genes, -2000 to 2000 bp, not over-represented

Promoter Length		Gene Number 100	200	400
up	7000bp	N	N	N
	4000bp	N	N	N
	2000bp	N	N	N
down	7000bp	N	N	N
	4000bp	N	N	N
	2000bp	N	N	N

Myb

29. The output of oPOSSUM for E-GEOD-5475

TF	TF Class	TF Supergroup	IC	Background gene hits	Background gene non-hits	Target gene hits	Target gene non-hits	Background TFBS hits	Background TFBS rate	Target TFBS hits	Target TFBS rate	Z-score	Fisher score
NR1H2-RXRA	NUCLEAR RECEPTOR	vertebrate	27.878	71	15079	3	140	72	0.0000	4	0.0003	17.14	3.206e-02
HNF4A	NUCLEAR RECEPTOR	vertebrate	9.617	5180	9970	62	81	7908	0.0037	94	0.0049	10.11	1.460e-02
Ar	NUCLEAR RECEPTOR	vertebrate	15.703	376	14774	7	136	395	0.0003	7	0.0006	8.647	6.832e-02
HNF1A	HOMEO	vertebrate	15.548	1921	13229	23	120	2495	0.0012	28	0.0016	4.554	1.386e-01
Evi1	ZN-FINGER, C2H2	vertebrate	17.909	843	14307	11	132	949	0.0005	11	0.0006	3.229	1.759e-01
RORA_2	NUCLEAR RECEPTOR	vertebrate	17.425	1858	13292	20	123	2200	0.0011	23	0.0013	2.844	3.015e-01
NR2F1	NUCLEAR RECEPTOR	vertebrate	15.924	2872	12278	30	113	3587	0.0018	36	0.0020	2.653	3.006e-01
TLX1-NFIC	HOMEO/CAAT	vertebrate	19.665	534	14616	6	137	562	0.0003	6	0.0003	1.601	3.929e-01
MAX	bHLH-ZIP	vertebrate	12.685	6081	9069	51	92	10424	0.0037	97	0.0039	1.34	8.800e-01
RREB1	ZN-FINGER, C2H2	vertebrate	22.278	721	14429	8	135	808	0.0006	8	0.0006	1.294	3.731e-01

PPARI, 200 up-regulated genes, -2000 to 2000 bp, not over-represented

TF	TF Class	TF Supergroup	IC	Background gene hits	Background gene non-hits	Target gene hits	Target gene non-hits	Background TFBS hits	Background TFBS rate	Target TFBS hits	Target TFBS rate	Z-score	Fisher score
SP1	ZN-FINGER, C2H2	vertebrate	9.719	9932	5218	114	30	35108	0.0125	476	0.0165	19.09	2.703e-04
NFYA	CAAT-BOX	vertebrate	12.925	4645	10505	57	87	7556	0.0043	104	0.0058	11.85	1.458e-02
HNF4A	NUCLEAR RECEPTOR	vertebrate	9.617	5180	9970	68	76	7908	0.0037	110	0.0050	11.39	8.829e-04
NR1H2-RXRA	NUCLEAR RECEPTOR	vertebrate	27.878	71	15079	3	141	72	0.0000	3	0.0002	10.67	3.262e-02
MZF1_1-4	ZN-FINGER, C2H2	vertebrate	8.586	13837	1313	135	9	154947	0.0332	1744	0.0363	9.158	1.922e-01
REST	ZN-FINGER, C2H2	vertebrate	22.958	190	14960	5	139	202	0.0001	5	0.0003	8.736	3.726e-02
IRF2	TRP-CLUSTER	vertebrate	21.134	552	14598	10	134	588	0.0004	11	0.0007	8.467	3.981e-02
IRF1	TRP-CLUSTER	vertebrate	16.008	4150	11000	51	93	6213	0.0027	81	0.0034	7.335	2.193e-02
RXRA-VDR	NUCLEAR RECEPTOR	vertebrate	20.451	269	14881	6	138	276	0.0001	6	0.0003	7.17	4.584e-02
Pax5	PAIRED	vertebrate	12.432	1352	13798	19	125	1534	0.0011	22	0.0015	6.937	5.640e-02

PPARI, 200 down-regulated genes, -2000 to 2000 bp, not over-represented

Promoter Length		Gene Number 100	200	400
up	7000bp	N	N	N
	4000bp	N	N	N
	2000bp	N	N	W
down	7000bp	S	N	N
	4000bp	N	N	N
	2000bp	W	N	S

PPARI

30. The output of oPOSSUM for E-GEOD-6846

TF	TF Class	TF Supergroup	IC	Background gene hits	Background gene non-hits	Target gene hits	Target gene non-hits	Background TFBS hits	Background TFBS rate	Target TFBS hits	Target TFBS rate	Z-score	Fisher score
SRY	HMG	vertebrate	9.193	11001	4149	101	28	53158	0.0171	592	0.0196	10.22	8.755e-02
SRF	MADS	vertebrate	17.965	535	14615	6	123	583	0.0003	12	0.0005	9.191	3.069e-01
Foxd3	FORKHEAD	vertebrate	12.945	8235	6915	78	51	25203	0.0108	282	0.0125	8.389	9.667e-02
Sox5	HMG	vertebrate	10.831	11265	3885	96	33	57233	0.0143	628	0.0162	8.274	5.403e-01
SOX9	HMG	vertebrate	9.079	8524	6626	83	46	22208	0.0071	251	0.0083	7.319	3.896e-02
FOXF2	FORKHEAD	vertebrate	14.824	2629	12521	30	99	3571	0.0018	46	0.0024	7.223	5.388e-02
MIZF	ZN-FINGER, C2H2	vertebrate	13.197	1637	13513	24	105	1934	0.0007	28	0.0010	6.723	5.911e-03
Nkx2-5	HOMEO	vertebrate	8.270	12616	2534	114	15	96540	0.0241	1010	0.0261	6.488	7.207e-02
Nobox	HOMEO	vertebrate	9.573	10484	4666	92	37	42827	0.0122	461	0.0136	6.395	3.400e-01
NFKB1	REL	vertebrate	15.627	2687	12463	32	97	3691	0.0015	47	0.0019	6.201	2.768e-02

STAT2, 200 up-regulated genes, -2000 to 2000 bp, not over-represented

TF	TF Class	TF Supergroup	IC	Background gene hits	Background gene non-hits	Target gene hits	Target gene non-hits	Background TFBS hits	Background TFBS rate	Target TFBS hits	Target TFBS rate	Z-score	Fisher score
Foxd3	FORKHEAD	vertebrate	12.945	8235	6915	70	40	25203	0.0108	261	0.0135	12.44	3.134e-02
Nkx2-5	HOMEO	vertebrate	8.270	12616	2534	95	15	96540	0.0241	932	0.0281	12.31	2.345e-01
Lhx3	HOMEO	vertebrate	16.354	3615	11535	39	71	6013	0.0028	72	0.0040	11.25	4.291e-03
REST	ZN-FINGER, C2H2	vertebrate	22.958	190	14960	4	106	202	0.0001	5	0.0004	11.09	5.174e-02
Sox5	HMG	vertebrate	10.831	11265	3885	86	24	57233	0.0143	559	0.0168	10.22	2.119e-01
SRY	HMG	vertebrate	9.193	11001	4149	85	25	53158	0.0171	512	0.0198	10.15	1.624e-01
FOXF2	FORKHEAD	vertebrate	14.824	2629	12521	29	81	3571	0.0018	44	0.0027	9.839	1.198e-02
Prrx2	HOMEO	vertebrate	9.063	11710	3440	97	13	80966	0.0145	779	0.0168	9.25	2.862e-03
Nobox	HOMEO	vertebrate	9.573	10484	4666	87	23	42827	0.0122	417	0.0144	9.245	1.410e-02
RREB1	ZN-FINGER, C2H2	vertebrate	22.278	721	14429	11	99	808	0.0006	12	0.0010	9.092	1.643e-02

STAT2, 200 down-regulated genes, -2000 to 2000 bp, not over-represented

Promoter Length		Gene Number 100	200	400
up	7000bp	N	N	N
	4000bp	N	N	N
	2000bp	N	N	N
down	7000bp	N	N	N
	4000bp	N	N	N
	2000bp	N	N	N

STAT2

31. The output of oPOSSUM for E-GEOD-11836

TF	TF Class	TF Supergroup	IC	Background gene hits	Background gene non-hits	Target gene hits	Target gene non-hits	Background TFBS hits	Background TFBS rate	Target TFBS hits	Target TFBS rate	Z-score	Fisher score
NFYA	CAAT-BOX	vertebrate	12.925	4645	10505	57	86	7556	0.0043	106	0.0062	14.66	1.249e-02
TLX1-NFIC	HOMEO/CAAT	vertebrate	19.665	534	14616	10	133	562	0.0003	11	0.0006	8.635	3.169e-02
NR2F1	NUCLEAR RECEPTOR	vertebrate	15.924	2872	12278	39	104	3587	0.0018	49	0.0025	8.59	9.965e-03
ZNF354C	ZN-FINGER, C2H2	vertebrate	8.958	13853	1297	135	8	136176	0.0292	1461	0.0318	8.122	1.297e-01
SP1	ZN-FINGER, C2H2	vertebrate	9.719	9932	5218	107	36	35108	0.0125	391	0.0142	7.727	1.135e-02
MYC-MAX	bHLH-ZIP	vertebrate	14.237	2630	12520	35	108	3253	0.0013	45	0.0018	7.572	2.004e-02
Pax4	PAIRED-HOMEO	vertebrate	11.004	145	15005	3	140	150	0.0002	3	0.0003	6.786	1.612e-01
RORA_1	NUCLEAR RECEPTOR	vertebrate	13.190	6302	8848	67	76	11069	0.0040	131	0.0048	6.653	1.186e-01
USF1	bHLH-ZIP	vertebrate	11.290	8718	6432	97	46	20331	0.0051	235	0.0060	6.5	7.745e-03
Arnt	bHLH	vertebrate	10.992	9176	5974	104	39	23233	0.0050	265	0.0058	5.857	1.659e-03

NKX31, 200 up-regulated genes, -2000 to 2000 bp, not over-represented

TF	TF Class	TF Supergroup	IC	Background gene hits	Background gene non-hits	Target gene hits	Target gene non-hits	Background TFBS hits	Background TFBS rate	Target TFBS hits	Target TFBS rate	Z-score	Fisher score
ZEB1	ZN-FINGER, C2H2	vertebrate	8.305	14221	929	139	9	180543	0.0387	1988	0.0416	7.88	5.773e-01
Myf	bHLH	vertebrate	15.914	7082	8068	81	67	14436	0.0062	175	0.0073	7.67	3.197e-02
NR2F1	NUCLEAR RECEPTOR	vertebrate	15.924	2872	12278	32	116	3587	0.0018	49	0.0024	7.511	2.334e-01
HLF	bZIP	vertebrate	11.147	4612	10538	51	97	7195	0.0031	92	0.0038	7.328	1.666e-01
NFIL3	bZIP	vertebrate	14.139	3293	11857	36	112	4969	0.0020	66	0.0025	6.965	2.515e-01
Mycn	bHLH-ZIP	vertebrate	10.443	9036	6114	100	48	22243	0.0048	269	0.0056	6.62	2.949e-02
REL	REL	vertebrate	10.515	7832	7318	86	62	16270	0.0058	193	0.0067	6.392	7.040e-02
NHLH1	bHLH	vertebrate	14.132	4061	11089	51	97	5936	0.0025	75	0.0031	6.25	2.494e-02
Arnt	bHLH	vertebrate	10.992	9176	5974	101	47	23233	0.0050	277	0.0058	6.135	3.332e-02
USF1	bHLH-ZIP	vertebrate	11.290	8718	6432	96	52	20331	0.0051	241	0.0059	5.942	4.272e-02

NKX31, 200 down-regulated genes, -2000 to 2000 bp, not over-represented

Promoter Length		Gene Number		
		100	200	400
up	7000bp	N	N	N
	4000bp	N	N	N
	2000bp	N	N	N
down	7000bp	N	N	N
	4000bp	N	N	N
	2000bp	N	N	N

NKX31

32. The output of oPOSSUM for E-MEXP-871

TF	TF Class	TF Supergroup	IC	Background gene hits	Background gene non-hits	Target gene hits	Target gene non-hits	Background TFBS hits	Background TFBS rate	Target TFBS hits	Target TFBS rate	Z-score	Fisher score
PPARG	NUCLEAR RECEPTOR	vertebrate	20.365	40	15110	2	119	40	0.0000	2	0.0002	12.86	4.364e-02
STAT1	Stat	vertebrate	18.431	1802	13348	27	94	2137	0.0011	29	0.0018	10.23	9.433e-04
ELF5	ETS	vertebrate	8.693	12664	2486	114	7	63365	0.0204	592	0.0232	9.604	3.981e-04
NFKB1	REL	vertebrate	15.627	2687	12463	27	94	3691	0.0015	46	0.0022	9.46	1.182e-01
GABPA	ETS	vertebrate	13.890	5358	9792	57	64	8422	0.0030	89	0.0039	7.573	5.332e-03
IRF1	TRP-CLUSTER	vertebrate	16.008	4150	11000	49	72	6213	0.0027	65	0.0034	6.803	1.304e-03
ELK4	ETS	vertebrate	14.123	4318	10832	49	72	6134	0.0020	66	0.0026	6.619	3.172e-03
IRF2	TRP-CLUSTER	vertebrate	21.134	552	14598	7	114	588	0.0004	8	0.0006	6.086	1.555e-01
RELA	REL	vertebrate	14.757	4606	10544	43	78	6841	0.0024	70	0.0030	5.85	1.312e-01
RREB1	ZN-FINGER, C2H2	vertebrate	22.278	721	14429	10	111	808	0.0006	10	0.0009	5.818	6.475e-02

HMGA2, 200 up-regulated genes, -2000 to 2000 bp, not over-represented

TF	TF Class	TF Supergroup	IC	Background gene hits	Background gene non-hits	Target gene hits	Target gene non-hits	Background TFBS hits	Background TFBS rate	Target TFBS hits	Target TFBS rate	Z-score	Fisher score
RORA_2	NUCLEAR RECEPTOR	vertebrate	17.425	1858	13292	27	110	2200	0.0011	37	0.0016	8.419	8.821e-03
Pax5	PAIRED	vertebrate	12.432	1352	13798	19	118	1534	0.0011	23	0.0014	5.437	3.698e-02
Nkx2-5	HOMEO	vertebrate	8.270	12616	2534	118	19	96540	0.0241	1186	0.0255	5.038	2.214e-01
TLX1-NFIC	HOMEO/CAAT	vertebrate	19.665	534	14616	10	127	562	0.0003	10	0.0004	5.017	2.455e-02
NFIL3	bZIP	vertebrate	14.139	3293	11857	39	98	4969	0.0020	68	0.0023	4.436	3.927e-02
SOX9	HMG	vertebrate	9.079	8524	6626	91	46	22208	0.0071	282	0.0078	4.428	1.010e-02
SRY	HMG	vertebrate	9.193	11001	4149	112	25	53158	0.0171	651	0.0180	3.973	8.928e-03
Sox5	HMG	vertebrate	10.831	11265	3885	112	25	57233	0.0143	700	0.0151	3.536	2.718e-02
HLF	bZIP	vertebrate	11.147	4612	10538	51	86	7195	0.0031	93	0.0034	3.516	5.405e-02
IRF2	TRP-CLUSTER	vertebrate	21.134	552	14598	8	129	588	0.0004	9	0.0005	3.461	1.305e-01

HMGA2, 200 down-regulated genes, -2000 to 2000 bp, not over-represented

Promoter Length		Gene Number 100	200	400
up	7000bp	N	N	N
	4000bp	N	N	N
	2000bp	N	N	N
down	7000bp	N	N	N
	4000bp	N	N	N
	2000bp	N	N	N

HMGA2

33. The output of oPOSSUM for E-MEXP-1413

TF	TF Class	TF Supergroup	IC	Background gene hits	Background gene non-hits	Target gene hits	Target gene non-hits	Background TFBS hits	Background TFBS rate	Target TFBS hits	Target TFBS rate	Z-score	Fisher score
ZEB1	ZN-FINGER, C2H2	vertebrate	8.305	14221	929	109	4	180543	0.0387	1737	0.0423	9.167	1.720e-01
Hand1-Tcfe2a	bHLH	vertebrate	10.144	10729	4421	88	25	35554	0.0127	360	0.0146	8.402	5.888e-02
ZNF354C	ZN-FINGER, C2H2	vertebrate	8.958	13853	1297	104	9	136176	0.0292	1313	0.0320	8.126	4.958e-01
Myf	bHLH	vertebrate	15.914	7082	8068	66	47	14436	0.0062	153	0.0074	7.943	8.674e-03
NR2F1	NUCLEAR RECEPTOR	vertebrate	15.924	2872	12278	30	83	3587	0.0018	42	0.0024	6.904	3.052e-02
Bapx1	HOMEO	vertebrate	8.542	11579	3571	91	22	44134	0.0142	428	0.0156	5.995	1.815e-01
MYC-MAX	bHLH-ZIP	vertebrate	14.237	2630	12520	29	84	3253	0.0013	38	0.0017	5.763	1.737e-02
MAX	bHLH-ZIP	vertebrate	12.685	6081	9069	52	61	10424	0.0037	109	0.0044	5.658	1.206e-01
Roaz	ZN-FINGER, C2H2	vertebrate	17.925	4153	10997	41	72	6324	0.0034	66	0.0040	5.329	2.503e-02
NFYA	CAAT-BOX	vertebrate	12.925	4645	10505	41	72	7556	0.0043	77	0.0050	5.11	1.182e-01

E2F2, 200 up-regulated genes, -2000 to 2000 bp, not over-represented

TF	TF Class	TF Supergroup	IC	Background gene hits	Background gene non-hits	Target gene hits	Target gene non-hits	Background TFBS hits	Background TFBS rate	Target TFBS hits	Target TFBS rate	Z-score	Fisher score
MZF1_1-4	ZN-FINGER, C2H2	vertebrate	8.586	13837	1313	150	4	154947	0.0332	2121	0.0376	14.1	2.208e-03
CREB1	bZIP	vertebrate	12.605	4951	10199	69	85	7418	0.0032	126	0.0045	13.24	1.194e-03
Pax4	PAIRED-HOMEO	vertebrate	11.004	145	15005	4	150	150	0.0002	5	0.0004	12.88	6.369e-02
MZF1_5-13	ZN-FINGER, C2H2	vertebrate	9.400	10713	4437	121	33	40747	0.0146	564	0.0166	10.14	1.828e-02
SP1	ZN-FINGER, C2H2	vertebrate	9.719	9932	5218	121	33	35108	0.0125	484	0.0143	9.097	3.190e-04
Roaz	ZN-FINGER, C2H2	vertebrate	17.925	4153	10997	58	96	6324	0.0034	97	0.0043	9.049	3.773e-03
RORA_2	NUCLEAR RECEPTOR	vertebrate	17.425	1858	13292	34	120	2200	0.0011	39	0.0016	8.945	4.953e-04
TEAD1	TEA	vertebrate	15.678	3102	12048	41	113	4137	0.0018	67	0.0024	8.268	4.067e-02
ZNF354C	ZN-FINGER, C2H2	vertebrate	8.958	13853	1297	149	5	136176	0.0292	1769	0.0313	7.371	7.668e-03
Arnt-Ahr	bHLH	vertebrate	9.532	11906	3244	126	28	55098	0.0118	741	0.0131	7.051	1.922e-01

E2F2, 200 down-regulated genes, -2000 to 2000 bp, not over-represented

Promoter Length		Gene Number 100	200	400
up	7000bp	N	N	N
	4000bp	N	N	N
	2000bp	N	N	N
down	7000bp	N	N	N
	4000bp	N	N	N
	2000bp	N	N	N

E2F2
